# Lysosomes Signal through the Epigenome to Regulate Longevity across Generations

**DOI:** 10.1126/science.adn8754

**Published:** 2025-09-25

**Authors:** Qinghao Zhang, Weiwei Dang, Meng C. Wang

**Affiliations:** 1 Huffington Center on Aging, Baylor College of Medicine; Houston TX, USA 77030.; 2 Janelia Research Campus, Howard Hughes Medical Institute; Ashburn VA, USA 20147.

## Abstract

The epigenome is sensitive to metabolic inputs and crucial for aging. Lysosomes act as a signaling hub to sense metabolic cues and regulate longevity. We find that lysosomal metabolic pathways signal through the epigenome to regulate transgenerational longevity in *Caenorhabditis elegans*. Activation of lysosomal lipid signaling and lysosomal adenosine monophosphate-activated protein kinase (AMPK) or reduction of lysosomal mechanistic-target-of-rapamycin (mTOR) signaling increased expression of a histone H3.3 variant and increased its methylation on K79, leading to lifespan extension across multiple generations. This transgenerational pro-longevity effect required intestine-to-germline transportation of histone H3.3 and a germline-specific H3K79 methyltransferase, and was recapitulated by overexpressing H3.3 or the H3K79 methyltransferase. Thus, signals from a lysosome affect the epigenome and link the soma and germline to mediate transgenerational inheritance of longevity.

Lysosomes are pivotal in sensing nutrient availability. Starvation increases transcription of lysosomal lipase-like 4 (LIPL-4), recruits adenosine monophosphate-activated protein kinase (AMPK) to lysosomes, but suppresses lysosomal activation of mechanistic-target-of-rapamycin complex I (mTORC1) (1–3). LIPL-4, AMPK, mTORC1 and starvation have been linked to longevity regulation (4). Starvation also induces epigenetic inheritance across generations in diverse organisms (5, 6). However, it remains unclear whether and how lysosomal signaling pathways modulate epigenetic memories and transgenerational longevity.

## Lysosomal lipolysis elevates H3K79 methylation and promotes transgenerational longevity

**Fig. 1.**
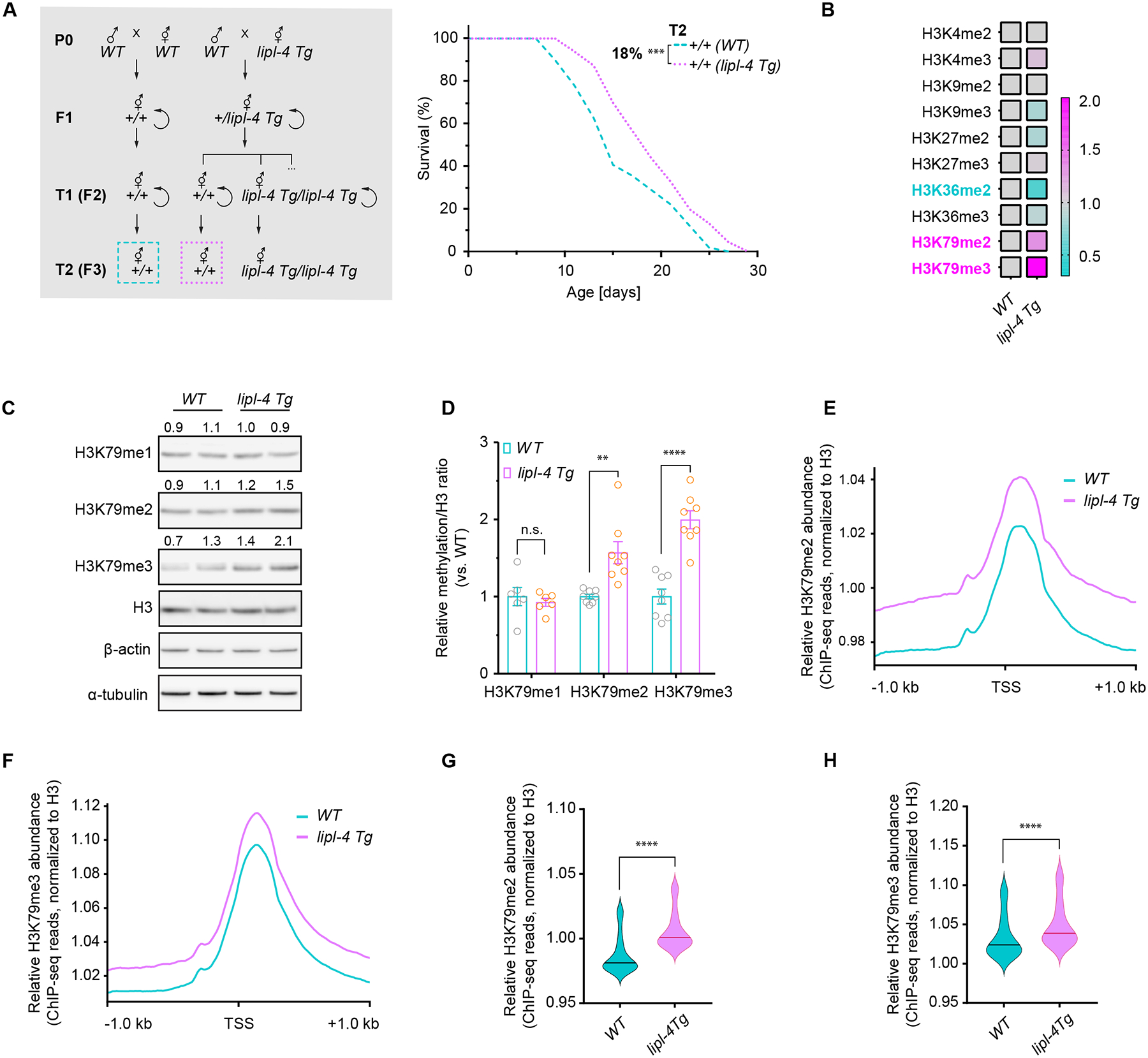
Lysosomal lipolysis induces transgenerational inheritance of longevity and H3K79 methylation. (**A**) T2 WT descendants (+/+ *(lipl-4 Tg)*), which are second transgenerational WT from P0 *lipl-4 Tg* worms, exhibit extended lifespan compared to WT controls (+/+ *(WT)*) from P0 WT worms. With n = 90/replicate, 5 biological replicates; log-rank test followed by Fisher’s method: ***p < 0.001. Percentage of lifespan extension (lower vs. upper) is labeled. Summary of lifespan replicates shown in table S1. Scheme describing genetic crosses and transgenerational groups (framed with dotted boxes) used in lifespan analyses. (**B**) The heatmap reveals the ratio of histone H3 PTM levels in *lipl-4 Tg* vs. WT worms from Western blot screens. (**C**) Western blot images show levels of H3K79 mono- (me1), di- (me2) and tri-methylation (me3), histone H3, β-actin and ⍺-tubulin in WT and *lipl-4 Tg* worms. (**D**) The bar chart summarizes all biologically independent replicates from Western blots. Error bars represent mean ± standard error of the mean (s.e.m.), n.s. *p* > 0.05, **p < 0.01, ****p < 0.0001 (unpaired t-test, Welch’s correction). (**E**-**H**) Enhanced deposition (±1 kb centered over TSS) of both H3K79me2 and H3K79me3 was observed in *lipl-4 Tg* vs. WT worms, which is quantified in violin plots (central lines denote median values). ****p < 0.0001 (Wilcoxon test).

As a lysosomal acid lipase, LIPL-4 overexpression in the intestine, the major metabolic tissue of worms, induces lysosomal lipolysis and promotes longevity (7, 8). When back crossing long-lived *lipl-4* transgenic (*lipl-4 Tg*) worms with wild-type (WT) worms, we noticed that WT descendants from the cross showed extended lifespan, indicating that LIPL-4-induced lysosomal lipolysis might promote longevity across generations. To confirm this, we crossed *lipl-4 Tg* hermaphrodites with WT males and measured lifespan of F3 WT descendants [designated as second transgenerational WT (T2 WT)] (Fig. 1A). In parallel, WT hermaphrodites were crossed with WT males and F3 progeny were used as WT controls (Fig. 1A). T2 WT descendants from the *lipl-4 Tg* cross lived longer than WT controls (Fig. 1A and table S1).

Epigenetic changes mediate transgenerational inheritance. In *C. elegans,* histone H3 lysine 4 trimethylation (H3K4me3) and histone H3 lysine 9 dimethylation (H3K9me2) are associated with transgenerational longevity (9, 10). However, the abundance of neither H3K4me3 nor H3K9me2 was altered in *lipl-4 Tg* worms (Fig. 1B and fig. S1A). We compared amounts of other histone H3 methylations, including H3K4me2, H3K9me3, H3K27me2, H3K27me3, H3K36me2, H3K36me3, H3K79me2 and H3K79me3, between day-1 adult *lipl-4 Tg* and WT worms (Fig. 1B and fig. S1A). Amounts of H3K79me2 and H3K79me3 were both increased in *lipl-4 Tg* worms (Fig. 1B–D, and fig. S1).

We also conducted chromatin immunoprecipitation with antibodies to H3K79me2, H3K79me3, and H3 from extracts from day-1 adult WT and *lipl-4 Tg* worms followed by next-generation sequencing (ChIP-seq). The signal for H3K79me2 or H3K79me3 was normalized to total H3. In comparison with the WT genome, the *lipl-4 Tg* genome exhibited increased abundance of H3K79me2 and H3K79me3, spanning 1 kb downstream and upstream of transcription start sites (TSS) (Fig. 1E–H). These results support that H3K79me2 and H3K79me3 abundances were increased in *lipl-4 Tg* worms.

## Lysosomal lipolysis transcriptionally upregulates H3.3 variant HIS-71

**Fig. 2.**
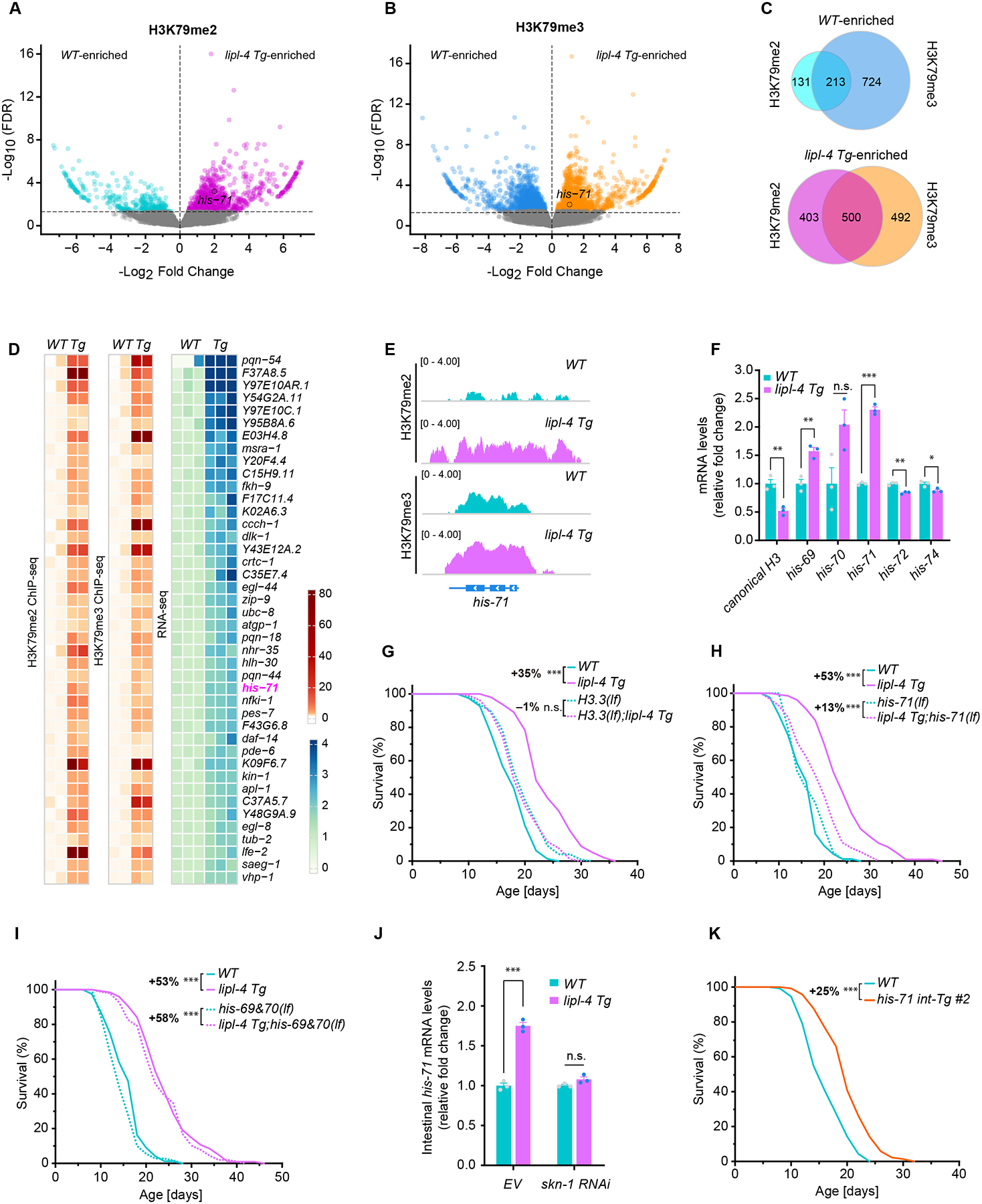
Lysosomal lipolysis transcriptionally upregulates H3.3 variant HIS-71 to promote longevity. (**A**, **B**) Volcano plots reveal gene loci with differential H3K79me2 or H3K79me3 deposition between WT and *lipl-4 Tg* worms. Cyan/blue, higher occupancy in WT; magenta/orange, higher in *lipl-4 Tg*; gray, no significant difference; encircled, *his-71*. (**C**) Venn diagrams show the numbers of genes enriched with H3K79me2 and/or me3 marks specifically in WT or *lipl-4 Tg* worms. (**D**) Heatmaps reveal gene candidates that were enriched with both H3K79me2 and H3K79me3 marks and were transcriptionally upregulated in *lipl-4 Tg* vs. WT worms. (**E**) Both H3K79me2 and H3K79me3 marks are increased on the promoter region and gene body of *his-71* in *lipl-4 Tg* vs. WT worms. (**F**) Relative expression changes of canonical H3 and H3.3 variants in *lipl-4 Tg* vs. WT worms. (**G**-**I**) The null mutant of all H3.3 variants or the loss-of-function (lf) mutant of *his-71*, but not the *his-60&70(lf)* mutant, suppresses lifespan extension caused by *lipl-4 Tg*. (**J**) Intestinal *his-71* expression is upregulated in *lipl-4 Tg* vs. WT worms, which is suppressed by *skn-1* RNAi inactivation. (**K**) Intestine-specific overexpression of *his-71* (*his-71 int-Tg*) extends lifespan. In (G-I and K), n = ~90/replicate, 3 biological replicates; log-rank test followed by Fisher’s method: n.s., *p* > 0.05, ***p < 0.001. Percentage of lifespan extension (lower vs. upper) is labeled. Summary of lifespan replicates shown in table S2. In (F and J), error bars represent mean ± s.e.m., n.s., *p* > 0.05, *p < 0.05, **p < 0.01, ***p < 0.001 (unpaired t-test, Welch’s correction).

H3K79 methylation serves as a mark for transcriptional activation (11, 12). To further decipher its molecular targets in transgenerational longevity regulation, we integrated the H3K79me2/me3 ChIP-seq data with the RNA-seq data from *lipl-4 Tg* worms, focusing on transcriptionally upregulated genes. From the ChIP-seq data, we found 1469 H3 sites showing statistically differential abundance of H3K79me2 between *lipl-4 Tg* and WT worms (Fig. 2A), and 2335 sites showing statistically differential abundance of H3K79me3 (Fig. 2B) (*p* < 0.01, FDR < 0.05). After mapping all these differential sites into genes, 500 genes showed enrichments of both H3K79me2 and H3K79me3 in *lipl-4 Tg* worms (Fig. 2C), with 42 of them transcriptionally upregulated in the RNA-seq analysis (fold change > 1.5, *p* < 0.01, FDR < 0.05) (Fig. 2D).

One of these targets was *his-71* (Fig. 2, D and E), encoding a histone variant H3.3. As a key epigenetic regulator of gene expression, H3.3 is incorporated into chromatin independently of replication in differentiated cells (13–15), and displays a greater propensity for K79 methylation than canonical H3 (16, 17). In *C. elegans*, five genes (*his-69*, *his-70*, *his-71*, *his-72,* and *his-74*) encode H3.3 (18, 19), and 15 genes encode canonical H3 (20). The presence of H3K79me2 or H3K79me3 on the other H3.3 variant genes or any canonical H3 genes was not increased in *lipl-4 Tg* worms (fig. S2A). Transcription of *his-71* and *his-69* was increased and that of canonical H3 was decreased in *lipl-4 Tg* worms (Fig. 2F). A null mutation of H3.3 reduced mean lifespan extension (from +35% to −1%) caused by *lipl-4 Tg* (Fig. 2G and table S2), as did *his-71* deletion (*his-71(lf)*) (from +53% to +13%) (Fig. 2H and table S2). On the other hand, deletion of *his-69* and *his-70* did not suppress the lifespan extension in *lipl-4 Tg* worms (Fig. 2I and table S2).

Transcription of *his-71* was increased in the intestine of *lipl-4 Tg* worms, where lysosomal lipolysis is induced (Fig. 2J, fig. S2B). Several transcription factors mediate the pro-longevity effect by *lipl-4 Tg*, including nuclear hormone receptor 49 (NHR-49), nuclear hormone receptor 80 (NHR-80), jun transcription factor homolog 1 (JUN-1), and skinhead family transcription factor 1 (SKN-1)—the homolog of nuclear factor erythroid-related factor 2 (Nrf2) (8, 21). A bioinformatic analysis of the promoter regions of 42 candidate genes identified through the integrated ChIP-seq and RNA-seq analysis revealed that 36 had promoter regions longer than 1 kb, and the SKN-1 binding motif was enriched in the promoter region of 18 genes, including *his-71* (fig. S2C). Depletion of *skn-1* by RNAi suppressed *his-71* transcriptional upregulation in the intestine of *lipl-4 Tg* worms (Fig. 2J). Furthermore, intestine-specific overexpression of *his-71* (*his-71 int-Tg*) prolonged lifespan (Fig. 2K, fig. S2D, table S2). These results indicate that LIPL-4-induced lysosomal lipolysis increases expression of the H3.3 variant *his-71* in the intestine to promote longevity.

## HIS-71 transports to germline and mediates transgenerational longevity

**Fig. 3.**
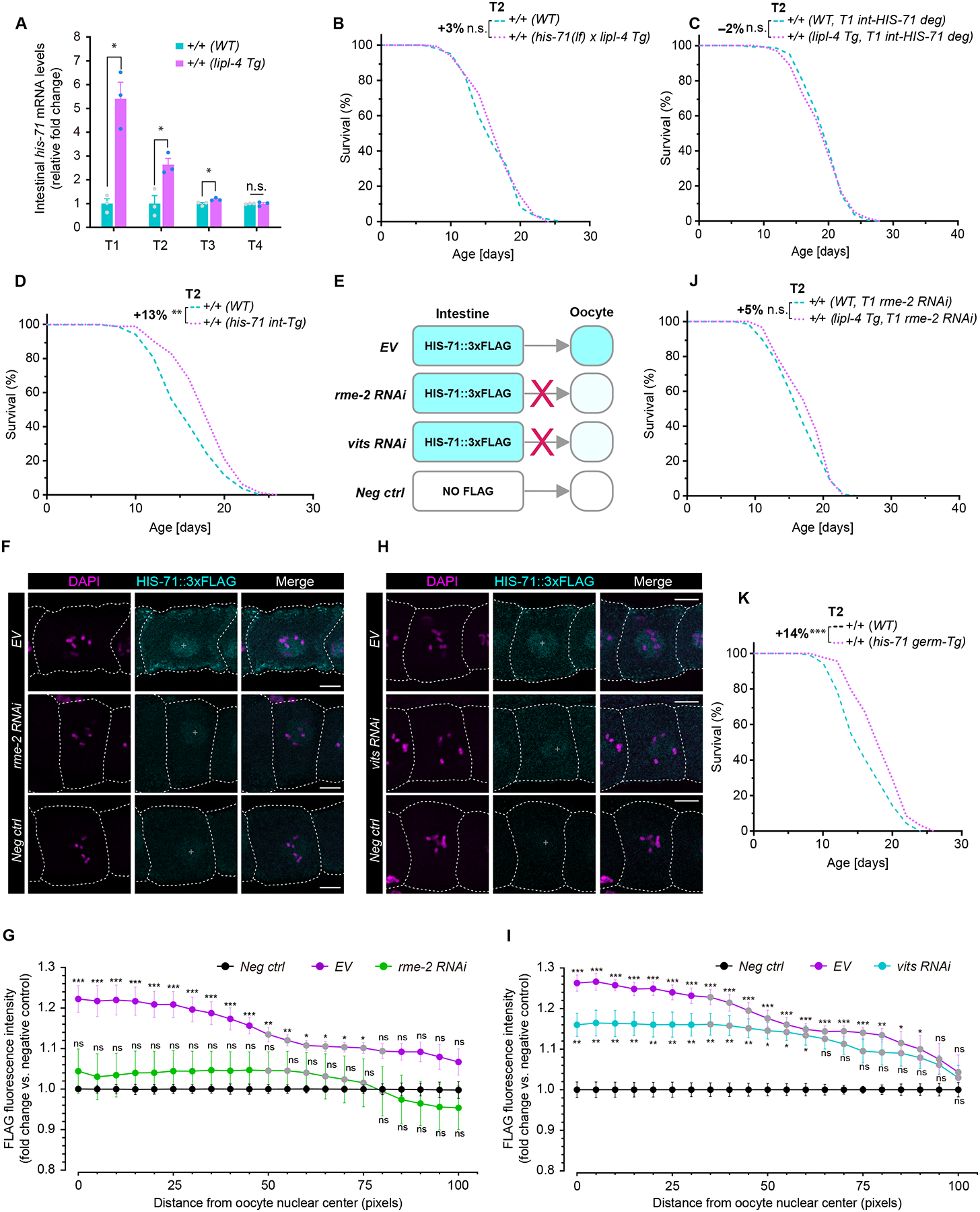
Intestinal HIS-71 and its transportation to germline contributes to transgenerational longevity. (**A**) Intestinal *his-71* transcription is elevated in WT descendants of *lipl-4 Tg* worms, with declines across generations. Error bars, mean ± s.e.m., n.s., *p* > 0.05, *p < 0.05 (unpaired t-test, Welch’s correction). (**B**) *his-71(lf)* mutant abrogates transgenerational longevity in T2 WT from *lipl-4 Tg*. (**C**) Intestine-specific HIS-71 degradation in T1 generation (T1 int-HIS-71 deg) abolishes transgenerational longevity caused by *lipl-4 Tg* in T2 generation. (**D**) Intestinal *his-71* overexpression promotes transgenerational longevity in T2 WT progeny. (**E**) Schematic for (F-I). Worms with intestine-specific *his-71::3×flag* overexpression were subjected to empty vector control (EV), *rme-2* or *vits* RNAi. Neg ctrl, intestine-specific overexpression of untagged *his-71*. (**F**, **H**) Images represent oocytes (outlined with dashed lines) at the proximal −2 position from germline immunostaining using antibody to FLAG (intestine-expressed HIS-71) and DAPI (nuclei). Scale bar = 10 μm (97.5 pixels). (**G**, **I**) Quantification of FLAG fluorescence intensity in −2 oocytes from the nuclear center (white crosses) to the periphery, with relative changes normalized to negative controls. n = 7–15 per replicate, 4 biological replicates. Error bars, mean ± s.e.m. Two-way ANOVA with Bonferroni’s test: n.s., *p* > 0.05, *p < 0.05, **p < 0.01, ***p < 0.001 (EV, *rme-2* or *vits* RNAi vs. Neg ctrl); gray dots, *p* > 0.05, otherwise, *p* < 0.05 (*rme-2* or *vits* RNAi vs. EV). (**J**) RNAi inactivation of *rme-2* in the T1 generation (T1 *rme-2* RNAi) abolishes the transmission of LIPL-4-induced transgenerational longevity to T2. (**K**) Germline *his-71* overexpression (*his-71 germ-Tg*) promoted transgenerational longevity in T2 WT progeny. In (B-D, J, K), n = 90/replicate, 3 biological replicates; log-rank test followed by Fisher’s method: n.s., *p* > 0.05, **p < 0.01, ***p < 0.001. Percentage of lifespan extension (lower vs. upper) is labeled. Summary of lifespan replicates shown in table S1.

Intestinal *his-71* transcription exhibited a progressive decline across generations in WT descendants from *lipl-4 Tg* worms, with the increase completely lost by T4 generation (Fig. 3A). This is consistent with the progressive loss of the longevity-promoting effect from T1 to T4 (fig. S3A–C, Fig. 1A and table S1). Furthermore, *his-71* deletion fully suppressed lifespan extension in T2 WT descendants from *lipl-4 Tg* cross (Fig. 3B, fig. S3D, and table S1). We also generated a CRISPR-modified line with degron-tagged endogenous HIS-71, and crossed it with *lipl-4 Tg* worms and a transgenic strain expressing the auxin-inducible F-box protein TIR1 selectively in the intestine. Using the crossed strain, we induced TIR1-mediated degradation of the degron-tagged HIS-71 protein selectively in the intestine by auxin treatment (fig. S3E). We administered auxin to T1 WT worms from *lipl-4 Tg* cross and found that intestine-specific degradation of HIS-71 suppressed lifespan extension in T2 WT descendants (Fig. 3C, fig. S3F, and table S1). We also confirmed that selectively overexpressing *his-71* in the intestine promoted transgenerational longevity (Fig. 3D and table S1). Thus, intestinal HIS-71 appears to contribute to the transgenerational pro-longevity effect conferred by lysosomal lipolysis.

Transgenerational inheritance requires the transmission of epigenetic information through the germline across generations. Given HIS-71’s impact on transgenerational longevity, we questioned whether this H3.3 variant could be transferred from the intestine to the germline. To investigate, we generated a transgenic strain that overexpressed HIS-71 tagged with three FLAG tags only in the intestine, without ectopic germline expression (table S3). When conducting immunostaining with antibodies to FLAG using dissected germline from this strain (Fig. 3E), we detected intestine-derived HIS-71::3×FLAG in oocytes within the germline (Fig. 3F–I). Vitellogenins are large lipid transfer proteins synthesized in the intestine and transported into oocytes by RME-2, a lipoprotein-like endocytic receptor. The observed HIS-71::3×FLAG signal in the germline was abolished by RNAi-mediated depletion of *rme-2* and reduced by RNAi-mediated depletion of vitellogenin genes (Fig. 3E–I). The depletion of *rme-2* did not affect increased transcription of *his-71* in the intestine of *lipl-4 Tg* worms (fig. S3G). These results suggest the transportation of HIS-71 from the intestine to the germline in a manner probably dependent on vitellogenin secretion and uptake.

When depleting *rme-2* to block the germline uptake of HIS-71 in T1 WT from *lipl-4 Tg* worms, we found it was sufficient to abrogate lifespan extension in T2 WT descendants (Fig. 3J and table S1). Thus, transportation of HIS-71 from the intestine to the germline appears to be required for the transgenerational pro-longevity effect. Furthermore, we induced selective degradation of HIS-71 in the germline through auxin treatment (fig. S3H), and this degradation in T1 WT descendants from the *lipl-4 Tg* cross abolished lifespan extension in T2 WT descendants (fig. S3I and table S1). Conversely, germline-specific overexpression of *his-71* (*his-71 germ-Tg*) promoted longevity across generations (Fig. 3K, fig. S3J, S3K, and tables S1, S2). The transcription of *his-71* was also upregulated in the germline of *lipl-4 Tg* worms (fig. S3L). Additionally, the intestine-specific overexpression of FLAG-tagged *his-71* led to increased expression of the endogenous *his-71* gene in the germline, which was abolished by hampering HIS-71 intestine-to-germline transportation through RNAi-mediated depletion of *rme-2* or vitellogenin genes (fig. S3M). These results indicate that in *lipl-4 Tg* worms, intestinal up-regulation of *his-71* transcription may increase HIS-71/H3.3 abundance in the germline, leading to transgenerational lifespan extension.

## Germline H3K79 methyltransferase promotes longevity

**Fig. 4.**
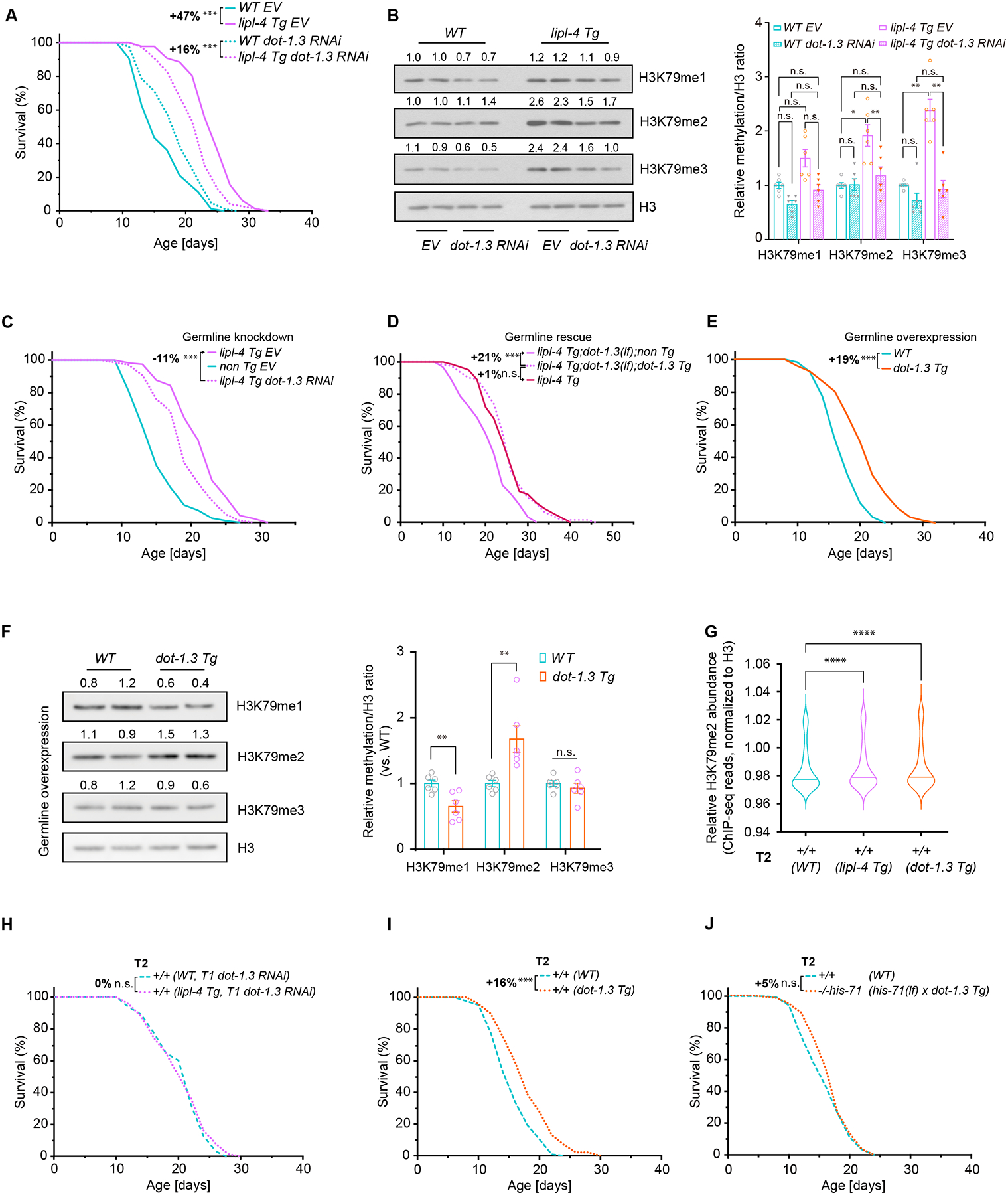
Germline H3K79 methyltransferase DOT-1.3 modulates longevity. (**A**) RNAi inactivation of *dot-1.3* reduced mean lifespan extension caused by *lipl-4 Tg* from 47% to 16%. (**B**) RNAi inactivation of *dot-1.3* suppressed the increased abundance of H3K79me2/3 in the *lipl-4 Tg* worms. Error bars represent mean ± s.e.m., 6 biological replicates, n.s. *p* > 0.05, *p < 0.05, **p < 0.01 (one-way ANOVA, Tukey test). (**C**) Germline-specific depletion of *dot-1.3* reduces lifespan extension caused by *lipl-4 Tg*. (**D**) Rescuing *dot-1.3* expression in the germline restored lifespan extension in *lipl-4 Tg;dot-1.3(lf)* worms. (**E**) Germline overexpression of *dot-1.3* results in lifespan extension. (**F**) Western blots show that germline-specific overexpression of *dot-1.3* elevates the H3K79me2 level. Error bars represent mean ± s.e.m., 6 biological replicates, n.s. *p* > 0.05, **p < 0.01 (unpaired t-test, Welch’s correction). (**G**) T2 WT descendants from *lipl-4 Tg* or *dot-1.3 Tg* exhibit elevated H3K79me2 deposition (±1 kb, TSS) compared to WT descendants from WT. ****p < 0.0001 (Wilcoxon test). Central lines denote median values of the data. (**H**) RNAi inactivation of *dot-1.3* in the T1 generation (T1 *dot-1.3* RNAi) abrogated the transgenerational longevity in T2 WT progeny from *lipl-4 Tg*. (**I**) T2 WT descendants from *dot-1.3 Tg* show extended lifespan. (**J**) Transgenerational longevity induced by *dot-1.3 Tg* is abolished by *his-71(lf)* mutant. In (A, C-E and H-J), n = ~90/replicate, 3 biological replicates; log-rank test followed by Fisher’s method: n.s., *p* > 0.05, ***p < 0.001. Percentage of lifespan extension (indicated by arrows, or lower vs. upper) is shown. Summary of lifespan replicates shown in tables S1, S2 and S4.

We also examined whether increased H3K79 methylation contributes to the transgenerational pro-longevity effect. H3K79 methylation is catalyzed by evolutionarily conserved H3K79 specific N-methyltransferase, disruptor of telomeric silencing 1 (Dot1) or DOT1-Like (DOT1L) (22). *C. elegans* has six putative H3K79 methyltransferases: DOT-1.1 to −1.5, and D1053.2 (23). We examined whether depleting DOT1 encoding genes suppressed lifespan extension in *lipl-4 Tg* worms. RNAi-mediated depletion of *dot-1.3* or *dot-1.1* decreased the mean lifespan extension by ~30% and ~20%, respectively, whereas depletion of *dot-1.2, dot-1.4, dot-1.5* or *D1053.2* did not (Fig. 4A, fig. S4A–E and table S4). The *dot-1.3(lf)* deletion reduced the mean lifespan extension by ~20%, while *dot-1.1(lf)* had no effect (fig. S4F, S4G and table S2). Furthermore, *dot-1.3* depletion suppressed increased abundance of H3K79me2 and H3K79me3 in *lipl-4 Tg* worms (Fig. 4B, fig. S4H and S4I).

To explore tissue-specificity of DOT-1.3, we generated a CRISPR-modified line with mNeonGreen-tagged endogenous DOT-1.3, revealing expression only in the germline, not in the intestine or other somatic tissues (fig. S5A). We depleted *dot-1.3* by RNAi specifically in the germline using a germline-specific *rde-1* rescuing strain (fig. S5B, S5C, table S4) and found that the germline-only depletion of *dot-1.3* decreased lifespan extension in *lipl-4 Tg* worms (Fig. 4C and table S4). Moreover, rescuing *dot-1.3* expression in the germline restored lifespan extension in *lipl-4 Tg* worms with *dot-1.3(lf)* deletion (Fig. 4D and table S2). Although no intestinal DOT-1.3 was detected in the CRISPR-modified line, we still examined its potential involvement in regulating longevity. RNAi-mediated depletion of *dot-1.3* specifically in the intestine did not suppress lifespan extension in *lipl-4 Tg* worms (fig. S5D, S5E and table S4). Restoring *dot-1.3* expression only in the intestine of *lipl-4 Tg* worms with *dot-1.3(lf)* deletion did not rescue lifespan extension (fig. S5F and table S2). Thus, germline DOT-1.3 appears to mediate the pro-longevity effect conferred by LIPL-4-induced lysosomal lipolysis.

We generated transgenic worms overexpressing *dot-1.3* selectively in the germline (*dot-1.3 Tg*), which exhibited extended lifespan (Fig. 4E and table S2). Although *dot-1.3* depletion suppressed increased abundance of both H3K79me2 and H3K79me3, overexpression in *dot-1.3 Tg* induced only H3K79me2 (Fig. 4F, fig. S5G and S5H), likely related to the limited methyl donor availability. These results suggest that the induction of H3K79me2 alone is sufficient to promote longevity and support the role of germline DOT-1.3 in longevity regulation.

## H3K79me2 transmission mediates transgenerational longevity

To examine whether H3K79me2 induction is transmitted across generations and mediates transgenerational longevity, we conducted ChIP-seq analysis with antibodies to H3K79me2 and total H3 using T2 WT descendants from the *lipl-4 Tg* cross and the *dot-1.3 Tg* cross with WT. Abundance of H3K79me2 was increased in both T2 WT descendants compared to WT controls (Fig. 4G). The *dot-1.3(lf)* deletion suppressed lifespan extension in T2 WT descendants from *lipl-4 Tg* cross (fig. S5I, S5J and table S1). RNAi-mediated depletion of *dot-1.3* only during the larval stage of T1 WT descendants from *lipl-4 Tg* worms abolished lifespan extension in T2 WT progeny (Fig. 4H and table S1). Moreover, T2 WT descendants from *dot-1.3 Tg* worms showed 16% mean lifespan extension compared to WT controls (Fig. 4I). Together, these results demonstrate the importance of DOT-1.3-mediated H3K79 methylation in regulating transgenerational longevity.

We also found that the pro-longevity effect of germline HIS-71 is augmented by K79 methylation. In the *dot-1.3(lf)* mutant, germline-specific overexpression of *his-71* failed to extend lifespan (fig. S5K and table S2). To further test the role of methylation, we generated transgenic worms overexpressing a non-methylatable mutant form of HIS-71 (K79A), in which lysine 79 is replaced with alanine (24). Germline-specific overexpression of HIS-71(K79A) led to only ~9% mean lifespan extension (fig. S5L and table S2), which is weaker than the >20% extension seen with WT HIS-71 overexpression (fig. S3J and table S2). Lifespan extension in *dot-1.3 Tg* worms was fully abrogated by complete deletion of H3.3 (fig. S5M and table S2), whereas *his-71(lf)* deletion alone was insufficient to suppress the extension (fig. S5N and table S2), suggesting that germline DOT-1.3 may target other H3.3 variants in the absence of HIS-71. Nevertheless, the lifespan extension observed in T2 WT from *dot-1.3 Tg* required HIS-71 (Fig. 4J, table S1). These findings reveal that methylation of HIS-71 by DOT-1.3 contributes to transgenerational longevity.

## Lysosome-related metabolic signaling regulates transgenerational longevity

**Fig. 5.**
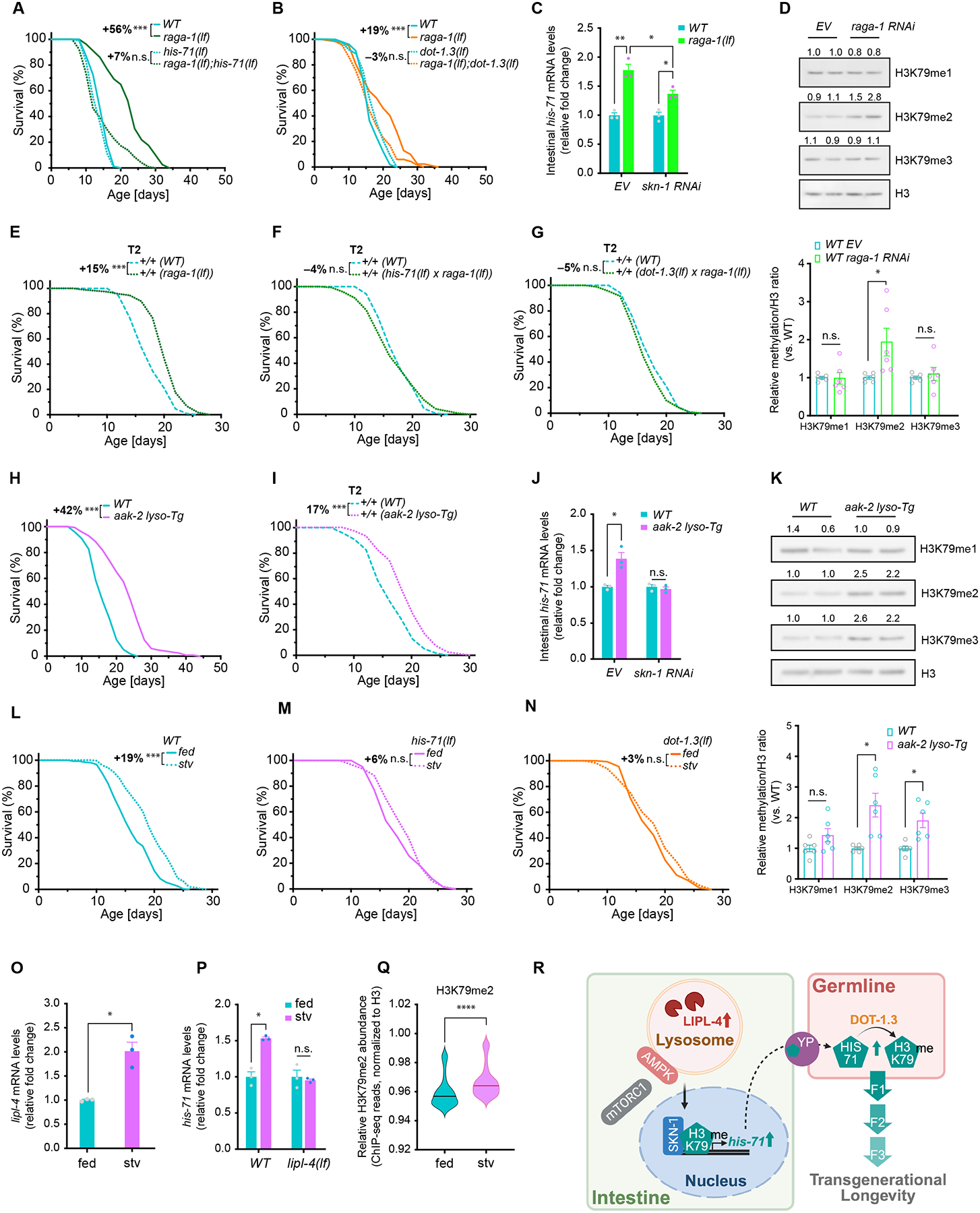
Lysosome-related metabolic signaling pathways regulate transgenerational longevity through DOT-1.3-mediated HIS-71 methylation. (**A**, **B**) Either *his-71(lf)* or *dot-1.3(lf)* mutant reduced lifespan extension caused by *raga-1(lf)* mutant. (**C**) Intestinal upregulation of *his-71* transcription in *raga-1(lf)* vs. WT is decreased by RNAi knockdown of *skn-1*. (**D**) RNAi knockdown of *raga-1* increased H3K79me2 level. (**E**-**G**) T2 WT from *raga-1(lf)* mutant exhibit extended lifespan (E), which was suppressed by *his-71(lf)* mutant (F) or *dot-1.3(lf)* mutant (G). (**H**) Transgenic worms with intestinal lysosome-tethered AAK-2 overexpression (*aak-2 lyso-Tg*) showed extended lifespan. (**I**) Transgenerational longevity was detected in T2 WT from *aak-2 lyso-Tg*. (**J**) Intestinal upregulation of *his-71* transcription is suppressed by RNAi knockdown of *skn-1* in *aak-2 lyso-Tg*. (**K**) Western blots show that abundance of H3K79me2 and me3 were increased in *aak-2 lyso-Tg* vs. WT. (**L**-**N**) Starvation (stv)-induced lifespan extension (L) is abolished in the *his-71(lf)* (M) and the *dot-1.3(lf)* (N) mutants. (**O**, **P**) Starvation increased *lipl-4* and *his-71* transcription in WT worms but failed to increase *his-71* transcription in *lipl-4(lf)* mutants. (**Q**) Starvation in WT worms elevates H3K79me2 deposition (±1 kb, TSS) when compared to fed condition. ****p < 0.0001 (Wilcoxon test). Central lines, median values. (**R**) A model schematic illustrating transgenerational longevity driven by lysosome-related metabolic signaling pathways through DOT-1.3-mediated HIS-71 methylation across intestine and germline. In Kaplan-Meier survival curves, n = 90/replicate; 3 biological replicates for (A, B, E-I, M, N) or 6 for (L); log-rank test followed by Fisher’s method: n.s., *p* > 0.05, ***p < 0.001. Percentage of lifespan extension (lower vs. upper) is shown. Summary of lifespan replicates shown in tables S1, S2 and S4. Error bars represent mean ± s.e.m.; 3 biological replicates for (C, J, O, P), or 6 for (D, K); n.s., *p* > 0.05, *p < 0.05 (unpaired t-test, Welch’s correction).

We next tested whether HIS-71 and DOT-1.3 contribute to other longevity regulatory mechanisms, including reduction in insulin and IGF-1 signaling (IIS), mTORC1 or mitochondrial electron transport chain (ETC) complexes, germline deficiency and caloric restriction (25). We crossed the *his-71(lf)* or *dot-1.3(lf)* mutant with the *daf-2(lf)* mutant (IIS reduction), the *raga-1(lf)* mutant (mTORC1 reduction), the *glp-1(lf)* mutant (germline deficiency) and the *eat-2(lf)* mutant (caloric restriction), or treated the *his-71(lf)* or *dot-1.3(lf)* mutant with *cco-1* RNAi (ETC reduction). The *dot-1.3(lf)* or *his-71(lf)* mutant only suppressed lifespan extension in the *raga-1(lf)* mutant, but did not affect the pro-longevity effect conferred by the other mutants or by ETC inhibition (Fig. 5A, 5B, fig. S6A–H, tables S2 and S4). We detected transcriptional upregulation of *his-71* in the intestine of the *raga-1(lf)* mutant, which was reduced by RNAi-mediated depletion of the SKN-1 transcription factor (Fig. 5C). Abundance of H3K79me2 was also increased with *raga-1* depletion (Fig. 5D, fig. S6I and S6J).

*raga-1* encodes the *C. elegans* homolog of RagA and RagB, ras-related GTP binding (Rag) proteins, that facilitate the lysosomal activation of mTORC1 by anchoring it to the lysosome surface (4, 26). Depletion of *raga-1* by RNAi did not further enhance the lifespan extension caused by *lipl-4 Tg* (fig. S6K and table S4), suggesting possible interaction between these two lysosomal pro-longevity mechanisms. To examine whether lysosomal mTORC1 signaling could modulate transgenerational longevity, we crossed the *raga-1(lf)* mutant hermaphrodites with WT males. T2 WT descendants from the cross showed statistically significant lifespan extension compared to WT controls (Fig. 5E and table S1). Furthermore, this lifespan extension was suppressed by either the *his-71(lf)* or *dot-1.3(lf)* mutant (Fig. 5F, 5G and table S1). These results support the role of lysosomal mTORC1 signaling in regulating transgenerational longevity through HIS-71 and DOT-1.3-mediated methylation.

Lysosomal docking of AMPK competes with mTORC1, preventing its lysosomal activation (3). AAK-2, a catalytic subunit of AMPK, is recruited to lysosomes in *lipl-4 Tg* worms, contributing to their lifespan extension (27). Thus, lysosomal recruitment of AMPK in the intestine might result in reduced mTORC1 signaling, thereby promoting transgenerational longevity as observed in WT descendants from the *lipl-4 Tg* and *raga-1 (lf)* worms. To test this, we generated a transgenic worm strain (*aak-2 lyso-Tg*) in which AAK-2 is tethered to lysosomes by fusion with lysosome-associated membrane protein 1 (LMP-1), and specifically overexpressed in the intestine. In parallel, a transgenic strain overexpressing a fluorescence protein mScarlet fused with LMP-1 in the intestine (*mScarlet lyso-Tg*) was generated as a control. The *aak-2 Tg* worms (28), in which *aak-2* is widely expressed without lysosomal tethering, were also included for analyses. We observed comparable lifespan extensions in *aak-2 lyso-Tg* (42%) and *aak-2 Tg* (31%) worms, but no lifespan extension in *mScarlet lyso-Tg* controls (Fig. 5H, fig. S6L, S6M, table S2).

Furthermore, we detected 17% mean lifespan extension in T2 WT progeny from *aak-2 lyso-Tg* worms (Fig. 5I), but not in T2 WT from *aak-2 Tg* worms (0%, fig. S6N and table S1). Similar to the findings in *lipl-4 Tg* and *raga-1(lf)* worms, *aak-2 lyso-Tg* worms exhibited upregulated intestinal *his-71* transcription in a SKN-1 dependent manner (Fig. 5J), as well as increased H3K79 methylation (Fig. 5K, fig. S6O, S6P). RNAi-mediated depletion of *dot-1.3* in T1 WT descendants from *aak-2 lyso-Tg* abrogated their transgenerational pro-longevity effect (fig. S6Q and table S1). Thus, lysosomal activation of AMPK appears to promote transgenerational longevity through DOT-1.3-mediated methylation of HIS-71.

Both lysosomal mTORC1 and AMPK signaling have been implicated in the metabolic response to starvation, an environmental change that induces epigenetic reprogramming and lifespan extension (4, 6, 29–31). Additionally, LIPL-4 is transcriptionally up-regulated upon fasting (1, 2). We subjected L1-stage larval worms to six days of starvation and assessed their adult lifespan after refeeding, with worms fed *Ad libitum* as the control. This starvation regimen (31) extended WT mean lifespan by 19% (Fig. 5L and table S2); however, this effect was abolished in the *his-71(lf)* and *dot-1.3(lf)* mutants (Fig. 5M, 5N and table S2), and was reduced to 10% in the *lipl-4(lf)* mutant (fig. S6R and table S2). Furthermore, we observed increased transcription of *lipl-4* and *his-71* and increased abundance of H3K79me2 in day-1 adult WT worms experiencing L1 larval starvation (Fig. 5O–Q). Starvation-induced transcription of *his-71* was fully suppressed in the *lipl-4(lf)* mutant (Fig. 5O). These results support that upregulation of LIPL-4 induces DOT-1.3-mediated HIS-71 methylation, contributing to longevity under physiological starvation conditions.

## Discussion

Based on our data, we propose a model (Fig. 5R): Induced lysosomal lipolysis triggers lysosomal AMPK activation and mTORC1 suppression, upregulating the transcription of H3.3, which is transported to the germline, modified by H3K79 methyltransferase, and transmitted across generations to promote longevity. H3.3-enriched epigenomes exhibit distinct transcriptional responses from canonical H3-enriched ones (13, 14). The H3.3-to-H3 transition restricts embryonic pluripotency during *C. elegans* development (20). H3.3 can help maintain genome stability (32–34) and repair DNA damage (35, 36). Increased H3.3 incorporation may preserve a youthful epigenomic state during aging.

Our findings reveal cross-tissue epigenetic coordination in transgenerational longevity, mediated by the yolk-based transport of histones. In *Drosophila*, maternal histones are transported from nurse cells to oocytes with yolk lipids (37). Yolk proteins resemble mammalian very-low-density lipoproteins (38), however, whether lipoproteins facilitate histone trafficking in mammals remains unknown.

## Supplementary Material

Table S1_20250204

Table S2_20250204

Table S4_20250204

Table S5_primers_20250204

Table S6_strains_20250204

adn8754_Supplementary_Final

Supplemental code_20250606

Table S3_qPCR Ct values_20250204

adn8754_ReproducibilityChecklist_20250608

Materials and Methods

Figs. S1 to S6

Tables S1 to S6 (Excel format)

References (39–58)

Custom Code (with usage instructions)

Cell Radial Profiling_v1.1.zip (newly developed ImageJ plugin)

MDAR Reproducibility Checklist

## Data Availability

All data are available in the main text or the supplementary materials. The RNA-seq data used in this study can be found in the NCBI Sequence Read Archive (SRA) and the accession codes for each biological sample are SAMN25414087, SAMN25414088, SAMN25414089, SAMN25414090, SAMN25414091, SAMN25414092. The ChIP-seq data used in this study has been submitted to GEO database and the GEO accession numbers are GSE235724, GSE290404, GSE290488. The newly generated code in this study can be found in the supplementary materials.
