# Supplementary material for: Lysosomes Signal through the Epigenome to Regulate Longevity across Generations
Source: Science. Author manuscript; Available in PMC 2026 Jan 24. (PMC12831228; doi:10.1126/science.adn8754)
Supplement: Table S1_20250204 [file NIHMS2127653-supplement-Table_S1_20250204.pdf]

Table S1. Summary of longitudinal survival analyses for transgenerational longevity studies.

Group	Fig	*Genotype*	Rep	Lifespan (mean ± s.e.)	# *P value*	§Lifespan Change	※Combined *p* value	Combined Lifespan (mean ± s.e)	Combined Lifespan Change	Total Number (censored)	Lab Code Strain Number of P0
1a	Fig.1A	*T2_WT +/+ from WTxWT*	#1	16.059 ± 0.715			<0.001	15.97 ± 0.24		90 (32)	N2
		T2_WT +/+ from WTxWT	#2	16.426 ± 0.671						90 (30)	
		T2_WT +/+ from WTxWT	#3	16.074 ± 0.694						90 (36)	
		T2_WT +/+ from WTxWT	#4	15.822 ± 0.338						90 (10)	
		T2_WT +/+ from WTxWT	#5	15.724 ± 0.338						90 (9)	
1b		*T2_WT +/+ from WT x lipl-4 Tg*	#1	19.113 ± 0.699	0.037	+19%		18.43 ± 0.24	+15%	90 (46)	MCW14
		T2_WT +/+ from WT x lipl-4 Tg	#2	19.427 ± 0.731	0.007	+18%				90 (44)	
		T2_WT +/+ from WT x lipl-4 Tg	#3	18.939 ± 0.676	0.015	+18%				90 (37)	
		T2_WT +/+ from WT x lipl-4 Tg	#4	17.612 ± 0.377	<0.001	+11%				90 (7)	
		T2_WT +/+ from WT x lipl-4 Tg	#5	18.022 ± 0.349	<0.001	+15%				90 (9)	

Group	Fig	*Genotype*	Rep	Lifespan (mean ± s.e.)	# *P value*	§Lifespan Change	※Combined *p* value	Combined Lifespan (mean ± s.e)	Combined Lifespan Change	Total Number (censored)	Lab Code Strain Number of P0
1a	fig. S3A	*T1_WT +/+ from WTxWT*	#1	17.069 ± 0.409				17.00 ± 0.24		90 (17)	N2
		T1_WT +/+ from WTxWT	#2	16.785 ± 0.449						90 (17)	
		T1_WT +/+ from WTxWT	#3	17.139 ± 0.412						90 (16)	
1b		*T1_WT +/+ from WT x lipl-4 Tg*	#1	19.345 ± 0.431	<0.001	+13%	0.000	19.56 ± 0.24	15%	90 (20)	MCW14
		T1_WT +/+ from WT x lipl-4 Tg	#2	19.863 ± 0.417	<0.001	+18%				90 (20)	
		T1_WT +/+ from WT x lipl-4 Tg	#3	19.485 ± 0.423	<0.001	+14%				90 (18)	
1a	fig. S3B	*T3_WT +/+ from WTxWT*	#1	14.984 ± 0.428				14.94 ± 0.25		90 (12)	N2
		T3_WT +/+ from WTxWT	#2	15.128 ± 0.417						90 (18)	
		T3_WT +/+ from WTxWT	#3	14.706 ± 0.451						90 (20)	
1b		*T3_WT +/+ from WT x lipl-4 Tg*	#1	16.630 ± 0.383	0.024	+11%	0.005	16.39 ± 0.22	10%	90 (9)	MCW14
		T3_WT +/+ from WT x lipl-4 Tg	#2	16.296 ± 0.401	0.074	+8%				90 (10)	
		T3_WT +/+ from WT x lipl-4 Tg	#3	16.234 ± 0.353	0.052	+10%				90 (13)	
1a	fig. S3C	*T4_WT +/+ from WTxWT*	#1	15.533 ± 0.350				15.35 ± 0.20		90 (13)	N2
		T4_WT +/+ from WTxWT	#2	15.082 ± 0.355						90 (14)	
		T4_WT +/+ from WTxWT	#3	15.434 ± 0.355						90 (13)	
1b		*T4_WT +/+ from WT x lipl-4 Tg*	#1	16.830 ± 0.393	0.011	+8%	0.002	16.45 ± 0.22	7%	90 (14)	MCW14
		T4_WT +/+ from WT x lipl-4 Tg	#2	16.022 ± 0.372	0.077	+6%				90 (14)	
		T4_WT +/+ from WT x lipl-4 Tg	#3	16.497 ± 0.389	0.039	+7%				90 (16)	

Group	Fig	*Genotype*	Rep	Lifespan (mean ± s.e.)	# *P value*	§Lifespan Change	※Combined *p* value	Combined Lifespan (mean ± s.e)	Combined Lifespan Change	Total number (censored)	Lab Code Strain Number of P0
1a	Fig.3B	*T2_WT +/+ from WT x WT*	#1	16.779 ± 0.428				16.49 ± 0.24		90 (12)	N2
		T2_WT +/+ from WT x WT	#2	16.222 ± 0.430						90 (20)	
		T2_WT +/+ from WT x WT	#3	16.432 ± 0.414						90 (13)	
1b		*T2_WT +/+ from his-71(lf) x lipl-4 Tg*	#1	16.851 ± 0.407	0.008	+0%	<0.001	16.94 ± 0.23	3%	90 (9)	RB1781/ MCW14
		T2_WT +/+ from his-71(lf) x lipl-4 Tg	#2	16.832 ± 0.397	0.014	+4%				90 (11)	
		T2_WT +/+ from his-71(lf) x lipl-4 Tg	#3	17.125 ± 0.398	0.002	+4%				90 (7)	

Group	Fig	*Genotype*	Rep	Lifespan (mean ± s.e.)	# *P value*	§Lifespan Change	※Combined *p* value	Combined Lifespan (mean ± s.e)	Combined Lifespan Change	Total number (censored)	Lab Code Strain Number of P0
Intestine-specific HIS-71 degradation induced by auxin-treatment on T1											
1a	Fig.3C	*T2_WT +/+ from WT x WT*	#1	19.711 ± 0.343				19.90 ± 0.20		90 (0)	MCW1541
		T2_WT +/+ from WT x WT	#2	19.862 ± 0.340						90 (4)	
		T2_WT +/+ from WT x WT	#3	20.123 ± 0.350						90 (4)	
1b		*T2_WT +/+ from WT x lipl-4 Tg*	#1	19.533 ± 0.390	0.880	-1%	0.949	19.54 ± 0.22	-2%	90 (0)	MCW1548
		T2_WT +/+ from WT x lipl-4 Tg	#2	19.689 ± 0.370	0.985	-1%				90 (0)	
		T2_WT +/+ from WT x lipl-4 Tg	#3	19.400 ± 0.390	0.754	-4%				90 (0)	

Group	Fig	*Genotype*	Rep	Lifespan (mean ± s.e.)	# *P value*	§Lifespan Change	※Combined *p* value	Combined Lifespan (mean ± s.e)	Combined Lifespan Change	Total number (censored)	Lab Code Strain Number of P0
1a	Fig.3D, 3K	*T2_WT +/+ from P0 WT*	#1	16.362 ± 0.447				16.29 ± 0.25		90 (13)	N2
		T2_WT +/+ from P0 WT	#2	16.300 ± 0.435						90 (12)	
		T2_WT +/+ from P0 WT	#3	16.212 ± 0.411						90 (11)	
1b	Fig.3D	*T2_WT +/+ from P0 his-71 int-Tg*	#1	18.342 ± 0.350	0.016	+12%	<0.001	18.21 ± 0.21	12%	90 (10)	MCW1350
		T2_WT +/+ from P0 his-71 int-Tg	#2	18.052 ± 0.372	0.017	+11%				90 (7)	
		T2_WT +/+ from P0 his-71 int-Tg	#3	18.242 ± 0.382	0.002	+13%				90 (9)	
	Fig.3K	*T2_WT +/+ from P0 his-71 germ-Tg*	#1	18.693 ± 0.389	0.001	+14%	<0.001	18.57 ± 0.23	14%	90 (5)	MCW1321
		T2_WT +/+ from P0 his-71 germ-Tg	#2	18.572 ± 0.409	0.001	+14%				90 (9)	
		T2_WT +/+ from P0 his-71 germ-Tg	#3	18.448 ± 0.391	<0.001	+14%				90 (8)	

Group	Fig	*Genotype*	Rep	Lifespan (mean ± s.e.)	# *P* value	§Lifespan Change	※Combined *p* value	Combined Lifespan (mean ± s.e)	Combined Lifespan Change	Total number (censored)	Lab Code Strain Number of P0
rme-2 RNAi on T1											
1a	Fig.3J	*T2_WT +/+ from WT x WT*	#1	16.846 ± 0.400				17.08 ± 0.23		90 (7)	N2
		T2_WT +/+ from WT x WT	#2	17.646 ± 0.383						90 (5)	
		T2_WT +/+ from WT x WT	#3	16.726 ± 0.400						90 (5)	
1b		*T2_WT +/+ from WT x lipl-4 Tg*	#1	18.044 ± 0.362	0.051	+7%	0.053	17.93 ± 0.21	5%	90 (3)	MCW14
		T2_WT +/+ from WT x lipl-4 Tg	#2	17.763 ± 0.375	0.861	+1%				90 (2)	
		T2_WT +/+ from WT x lipl-4 Tg	#3	17.970 ± 0.361	0.046	+7%				90 (2)	

Group	Fig	*Genotype*	Rep	Lifespan (mean ± s.e.)	# *P* value	§Lifespan Change	※Combined *p* value	Combined Lifespan (mean ± s.e)	Combined Lifespan Change	Total Number (censored)	Lab Code Strain Number of P0
Germline-specific HIS-71 degradation induced by auxin-treatment on T1											
2a	fig.S3I	*T2_WT +/+ from WT x WT*	#1	19.119 ± 0.442				19.18 ± 0.24		90 (2)	MCW1539
		T2_WT +/+ from WT x WT	#2	19.236 ± 0.387						90 (1)	
		T2_WT +/+ from WT x WT	#3	19.171 ± 0.417						90 (1)	
2b		*T2_WT +/+ from WT x lipl-4 Tg*	#1	19.659 ± 0.335	0.975	+3%	0.997	19.60 ± 0.20	2%	90 (2)	MCW1546
		T2_WT +/+ from WT x lipl-4 Tg	#2	19.545 ± 0.348	0.815	+2%				90 (2)	
		T2_WT +/+ from WT x lipl-4 Tg	#3	19.591 ± 0.347	0.952	+2%				90 (2)	

Group	Fig	*Genotype*	Rep	Lifespan (mean ± s.e.)	# *P* value	§Lifespan Change	※Combined *p* value	Combined Lifespan (mean ± s.e)	Combined Lifespan Change	Total number (censored)	Lab Code Strain Number of P0
1a	fig.S3K	*T1_WT +/+ from P0 WT*	#1	16.359 ± 0.395				16.56 ± 0.23		90 (9)	N2
		T1_WT +/+ from P0 WT	#2	16.486 ± 0.409						90 (11)	
		T1_WT +/+ from P0 WT	#3	16.839 ± 0.378						90 (15)	
1b		*T1_WT +/+ from P0 his-71 germ-Tg*	#1	19.083 ± 0.369	<0.001	+17%	<0.001	19.00 ± 0.21	15%	90 (12)	MCW1321
		T1_WT +/+ from P0 his-71 germ-Tg	#2	18.691 ± 0.383	0.001	+13%				90 (12)	
		T1_WT +/+ from P0 his-71 germ-Tg	#3	19.228 ± 0.351	<0.001	+14%				90 (11)	

Group	Fig	*Genotype*	Rep	Lifespan (mean ± s.e.)	# *P value*	§Lifespan Change	※Combined *p* value	Combined Lifespan (mean ± s.e)	Combined Lifespan Change	Total Number (censored)	Lab Code Strain Number of P0
1a	fig.S4S	*T2_WT +/+ from WTxWT*	#1	18.051 ± 0.472				17.98 ± 0.27		90 (15)	N2
		T2_WT +/+ from WTxWT	#2	18.027 ± 0.461						90 (15)	
		T2_WT +/+ from WTxWT	#3	17.857 ± 0.482						90 (15)	
1b		*T2_WT +/+ from dot-1.3(lf) x lipl-4 Tg*	#1	17.786 ± 0.393	0.266	-1%	0.046	17.37 ± 0.23	-3%	90 (22)	VC2294/MCW14
		T2_WT +/+ from dot-1.3(lf) x lipl-4 Tg	#2	17.405 ± 0.407	0.138	-3%				90 (23)	
		T2_WT +/+ from dot-1.3(lf) x lipl-4 Tg	#3	16.900 ± 0.418	0.045	-5%				90 (25)	

Group	Fig	*Genotype*	Rep	Lifespan (mean ± s.e.)	# *P value*	§Lifespan Change	※Combined *p* value	Combined Lifespan (mean ± s.e)	Combined Lifespan Change	Total Number (censored)	Lab Code Strain Number of P0
dot-1.3 RNAi on T1											
1a	Fig.4H	*T2_WT +/+ from WT x WT*	#1	19.132 ± 0.447				18.74 ± 0.26		90 (5)	N2
		T2_WT +/+ from WT x WT	#2	18.344 ± 0.468						90 (4)	
		T2_WT +/+ from WT x WT	#3	18.733 ± 0.440						90 (5)	
1b		*T2_WT +/+ from WT x lipl-4 Tg*	#1	18.898 ± 0.503	0.051	-1%	0.053	18.72 ± 0.27	0%	90 (2)	MCW14
		T2_WT +/+ from WT x lipl-4 Tg	#2	18.527 ± 0.476	0.861	+1%				90 (3)	
		T2_WT +/+ from WT x lipl-4 Tg	#3	18.729 ± 0.455	0.046	-0%				90 (1)	

Group	Fig	*Genotype*	Rep	Lifespan (mean ± s.e.)	# *P value*	§Lifespan Change	※Combined *p* value	Combined Lifespan (mean ± s.e)	Combined Lifespan Change	Total Number (censored)	Lab Code Strain Number of P0
1a	Fig.4I	*T2_WT +/+ from WT x WT*	#1	15.639 ± 0.406				15.76 ± 0.23		90 (17)	N2
		T2_WT +/+ from WT x WT	#2	15.840 ± 0.410						90 (16)	
		T2_WT +/+ from WT x WT	#3	15.804 ± 0.394						90 (14)	
1b		*T2_WT +/+ from WT x dot-1.3 Tg*	#1	18.339 ± 0.591	<0.001	+17%	<0.001	18.21 ± 0.31	16%	90 (26)	MCW1149
		T2_WT +/+ from WT x dot-1.3 Tg	#2	18.843 ± 0.533	<0.001	+19%				90 (29)	
		T2_WT +/+ from WT x dot-1.3 Tg	#3	17.480 ± 0.502	0.007	+11%				90 (25)	

Group	Fig	*Genotype*	Rep	Lifespan (mean ± s.e.)	# *P value*	§Lifespan Change	※Combined *p* value	Combined Lifespan (mean ± s.e)	Combined Lifespan Change	Total Number (censored)	Lab Code Strain Number of P0
1a	Fig.4J	*T2_WT +/+ from WT x WT*	#1	16.212 ± 0.413				16.22 ± 0.24		90 (14)	N2
		T2_WT +/+ from WT x WT	#2	16.262 ± 0.410						90 (8)	
		T2_WT +/+ from WT x WT	#3	16.192 ± 0.441						90 (11)	
1b		*T2_his-71 -/-from his-71(lf) x dot-1.3 Tg*	#1	17.072 ± 0.335	0.305	+5%	0.207	17.09 ± 0.22	5%	90 (9)	RB1781/ MCW1149
		T2_his-71 -/-from his-71(lf) x dot-1.3 Tg	#2	17.115 ± 0.398	0.188	+5%				90 (8)	
		T2_his-71 -/-from his-71(lf) x dot-1.3 Tg	#3	17.093 ± 0.407	0.255	+6%				90 (8)	

Group	Fig	*Genotype*	Rep	Lifespan (mean ± s.e.)	# *P value*	§Lifespan Change	※Combined *p* value	Combined Lifespan (mean ± s.e)	Combined Lifespan Change	Total Number (censored)	Lab Code Strain Number of P0
1a	Fig.5E, 5F,5G	*T2_WT +/+ from WT x WT*	#1	17.719 ± 0.373				17.71 ± 0.21		90 (11)	N2
		T2_WT +/+ from WT x WT	#2	17.728 ± 0.373						90 (9)	
		T2_WT +/+ from WT x WT	#3	17.677 ± 0.358						90 (9)	
1b	Fig.5E	*T2_WT +/+ from WT x raga-1(lf)*	#1	20.731 ± 0.336	<0.001	+17%	<0.001	20.43 ± 0.22	15%	90 (13)	VC222
		T2_WT +/+ from WT x raga-1(lf)	#2	20.317 ± 0.419	<0.001	+15%				90 (11)	
		T2_WT +/+ from WT x raga-1(lf)	#3	20.242 ± 0.405	<0.001	+15%				90 (6)	
1c	Fig.5F	*T2_WT +/+ from his-71(lf) x raga-1(lf)*	#1	17.232 ± 0.566	0.753	-3%	0.945	17.07 ± 0.31	-4%	90 (11)	RB1781/ VC222
		T2_WT +/+ from his-71(lf) x raga-1(lf)	#2	16.943 ± 0.481	0.605	-4%				90 (12)	
		T2_WT +/+ from his-71(lf) x raga-1(lf)	#3	17.023 ± 0.545	0.940	-4%				90 (13)	
1d	Fig.5G	*T2_WT +/+ from dot-1.3(lf) x raga-1(lf)*	#1	17.008 ± 0.387	0.240	-4%	0.059	16.82 ± 0.22	-5%	90 (11)	VC2294/ VC222
		T2_WT +/+ from dot-1.3(lf) x raga-1(lf)	#2	16.793 ± 0.381	0.108	-5%				90 (12)	
		T2_WT +/+ from dot-1.3(lf) x raga-1(lf)	#3	16.675 ± 0.381	0.089	-6%				90 (13)	

Group	Fig	*Genotype*	Rep	Lifespan (mean ± s.e.)	# *P value*	§Lifespan Change	※Combined *p* value	Combined Lifespan (mean ± s.e)	Combined Lifespan Change	Total Number (censored)	Lab Code Strain Number of P0
1a	Fig. 5I	*T2_WT +/+ from P0 WT*	#1	16.261 ± 0.429				16.38 ± 0.26		90 (10)	N2
		T2_WT +/+ from P0 WT	#2	16.382 ± 0.433						90 (10)	
		T2_WT +/+ from P0 WT	#3	16.499 ± 0.477						90 (9)	
1b		*T2_WT +/+ from P0 aak-2 lyso-Tg*	#1	19.010 ± 0.468	<0.001	+17%	<0.001	19.22 ± 0.26	17%	90 (11)	MCW1653
		T2_WT +/+ from P0 aak-2 lyso-Tg	#2	19.440 ± 0.466	<0.001	+19%				90 (11)	
		T2_WT +/+ from P0 aak-2 lyso-Tg	#3	19.201 ± 0.435	<0.001	+16%				90 (7)	

Group	Fig	*Genotype*	Rep	Lifespan (mean ± s.e.)	*# P value*	§Lifespan Change	※Combined *p* value	Combined Lifespan (mean ± s.e)	Combined Lifespan Change	Total Number (censored)	Lab Code Strain Number of P0
1a	fig. S5N	*T2_WT +/+ from P0 WT*	#1	16.755 ± 0.461				16.42 ± 0.27		90 (17)	N2
		T2_WT +/+ from P0 WT	#2	16.540 ± 0.456						90 (14)	
		T2_WT +/+ from P0 WT	#3	15.936 ± 0.510						90 (21)	
1b		*T2_WT +/+ from P0 aak-2 Tg*	#1	16.115 ± 0.432	0.274	-4%	0.683	16.48 ± 0.26	0%	90 (19)	WBM60
		T2_WT +/+ from P0 aak-2 Tg	#2	16.689 ± 0.476	0.880	+1%				90 (21)	
		T2_WT +/+ from P0 aak-2 Tg	#3	16.649 ± 0.435	0.575	+4%				90 (19)	

Group	Fig	*Genotype*	Rep	Lifespan (mean ± s.e.)	*# P value*	§Lifespan Change	※Combined *p* value	Combined Lifespan (mean ± s.e)	Combined Lifespan Change	Total Number (censored)	Lab Code Strain Number of P0
dot-1.3 RNAi on T1											
1a	fig.S5Q	*T2_WT +/+ from P0 WT*	#1	19.391 ± 0.351				19.15 ± 0.21		90 (3)	N2
		T2_WT +/+ from P0 WT	#2	19.168 ± 0.376						90 (3)	
		T2_WT +/+ from P0 WT	#3	19.192 ± 0.391						90 (3)	
1b		*T2_WT +/+ from P0 aak-2 lyso-Tg*	#1	18.273 ± 0.453	0.312	-6%	0.517	18.39 ± 0.25	-4%	90 (5)	MCW1653
		T2_WT +/+ from P0 aak-2 lyso-Tg	#2	18.493 ± 0.443	0.578	-4%				90 (6)	
		T2_WT +/+ from P0 aak-2 lyso-Tg	#3	18.405 ± 0.436	0.410	-4%				90 (4)	

P value: compare values between the different alphabet initiated with the same number and followed by the same replicate number (#) in the same frame using a log-rank test, e.g. "1a#1 vs. 1b#1".

§ Lifespan Changes are attached to the *p* value analyses by using "+" to indicate the increased lifespan percentage and using "-" to indicate the decreased lifespan percentage.

※ Combined *p* value of three independent replicates is calculated by the Fisher's method using the R package metap (v1.8).
