# Supplementary material for: Lysosomes Signal through the Epigenome to Regulate Longevity across Generations
Source: Science. Author manuscript; Available in PMC 2026 Jan 24. (PMC12831228; doi:10.1126/science.adn8754)
Supplement: Table S2_20250204 [file NIHMS2127653-supplement-Table_S2_20250204.pdf]

Table S2. Summary of longitudinal survival analyses in transgenetic strains and mutants.

Group	Fig	*Genotype*	Rep	Lifespanc (mean ± s.e.)	# *P value 1*	§Lifespan Change 1	※Combined *p* Value 1	& *P Value 2*	§Lifespan Change 2	※Combined *p* Value 2	Combined Lifespan (mean ± s.e.)	Combined lifespan Change 1	Combined lifespan Change 2	Total number (censored)	Lab Code Strain Number
1a		*WT*	#1	17.965 ± 0.407										90 (12)	N2
		WT	#2	17.770 ± 0.410							17.91 ± 0.24			90 (16)	
		WT	#3	17.989 ± 0.414										90 (15)	
1b		*lipl-4 Tg*	#1	24.062 ± 0.572	<0.001	+33.9%								90 (25)	MCW15
		lipl-4 Tg	#2	24.034 ± 0.624	<0.001	+35.3%	<0.001				24.19 ± 0.35	+35%		90 (26)	
	Fig. 2G	*lipl-4 Tg*	#3	24.471 ± 0.621	<0.001	+36.0%								90 (26)	
2a		*H3.3(lf)*	#1	20.380 ± 0.484				0.000	+13.4%					89 (10)	FAS43
		H3.3(lf)	#2	19.407 ± 0.430				0.005	+9.2%	<0.001	19.83 ± 0.27		+11%	90 (6)	
		H3.3(lf)	#3	19.722 ± 0.487				0.003	+9.6%					91 (7)	
2b		*lipl-4 Tg;H3.3(lf)*	#1	19.452 ± 0.582	0.260	-4.6%		<0.001	-19.2%					90 (31)	MCW1150
		lipl-4 Tg;H3.3(lf)	#2	19.501 ± 0.490	0.972	+0.5%	0.800	<0.001	-18.9%	<0.001	19.66 ± 0.31	-1%	-19%	90 (27)	
		lipl-4 Tg;H3.3(lf)	#3	20.032 ± 0.543	0.854	+1.6%		<0.001	-18.1%					90 (30)	

Group	Fig	*Genotype*	Rep	Lifespan (mean ± s.e.)	# *P value 1*	§Lifespan Change 1	※Combined *p* Value 1	& *P Value 2*	§Lifespan Change 2	※Combined *p* Value 2	Combined Lifespan (mean ± s.e.)	Combined lifespan Change 1	Combined lifespan Change 2	Total number (censored)	Lab Code Strain Number
1a		*WT*	#1	15.870 ± 0.510										90 (37)	N2
		WT	#2	16.224 ± 0.533							15.95 ± 0.30			90 (30)	
	Fig. 2H, 2I	*WT*	#3	15.717 ± 0.515										90 (33)	
1b		*lipl-4 Tg*	#1	24.490 ± 0.803	<0.001	+54.3%								90 (15)	MCW15
		lipl-4 Tg	#2	24.288 ± 0.677	<0.001	+49.7%	<0.001				24.45 ± 0.41	+53%		90 (15)	
		lipl-4 Tg	#3	24.576 ± 0.679	<0.001	+56.4%								90 (14)	
2a		*his-71(lf)*	#1	16.665 ± 0.491				0.210	+5.0%					90 (28)	RB1781
		his-71(lf)	#2	16.636 ± 0.489				0.603	+2.5%	0.171	16.73 ± 0.29		+5%	90 (27)	
	Fig. 2H	*his-71(lf)*	#3	16.919 ± 0.549				0.086	+7.6%					90 (34)	
2b		*lipl-4 Tg;his-71(lf)*	#1	18.998 ± 0.624	0.001	+14.0%		<0.001	-22.4%					90 (17)	MCW1271
		lipl-4 Tg;his-71(lf)	#2	18.754 ± 0.695	0.005	+12.7%	<0.001	<0.001	-22.8%	<0.001	18.96 ± 0.36	+13%	-22%	90 (16)	
		lipl-4 Tg;his-71(lf)	#3	19.112 ± 0.587	0.004	+13.0%		<0.001	-22.2%					90 (10)	
3a		*his-69&70(lf)*	#1	14.626 ± 0.462				0.079	-7.8%					90 (26)	FAS65
		his-69&70(lf)	#2	14.923 ± 0.492				0.065	-8.0%	0.040	14.88 ± 0.27		-7%	90 (28)	
		his-69&70(lf)	#3	15.078 ± 0.477				0.265	-4.1%					90 (28)	
3b	Fig. 2I	*lipl-4 Tg;his-69&70(lf)*	#1	23.949 ± 0.779	<0.001	+63.7%		0.641	-2.2%					90 (19)	MCW1270
		lipl-4 Tg;his-69&70(lf)	#2	23.812 ± 0.743	<0.001	+59.6%	<0.001	0.897	-2.0%	0.434	23.53 ± 0.42	+58%	-4%	90 (19)	
		lipl-4 Tg;his-69&70(lf)	#3	22.858 ± 0.674	<0.001	+51.6%		0.091	-7.0%					90 (17)	

Group	Fig	*Genotype*	Rep	Lifespan (mean ± s.e.)	*# P value 1*	§Lifespan Change 1	※Combined *p* Value 1				Combined Lifespan (mean ± s.e.)	Combined lifespan Change 1		Total number (censored)	Lab Code Strain Number
1a	fig. S2D	*WT*	#1	18.028 ± 0.465							18.36 ± 0.24			90 (33)	N2
		WT	#2	18.531 ± 0.403										90 (30)	
		WT	#3	18.483 ± 0.388										90 (25)	
1b		*his-71 int-Tg line1*	#1	20.310 ± 0.506	<0.001	+12.7%	<0.001				20.47 ± 0.28	11%		90 (14)	MCW1350
		his-71 int-Tg line1	#2	20.253 ± 0.485	<0.001	+9.3%								90 (17)	
		his-71 int-Tg line1	#3	20.855 ± 0.481	<0.001	+12.8%								90 (17)	
2a	Fig. 2K	*WT*	#1	16.362 ± 0.447							16.29 ± 0.25			90 (13)	N2
		WT	#2	16.300 ± 0.435										90 (12)	
		WT	#3	16.212 ± 0.411										90 (11)	
2b		*his-71 int-Tg line2*	#1	20.391 ± 0.467	<0.001	+24.6%	<0.001				20.32 ± 0.29	25%		90 (10)	MCW1350
		his-71 int-Tg line2	#2	20.080 ± 0.536	<0.001	+23.2%								90 (16)	
		his-71 int-Tg line2	#3	20.499 ± 0.489	<0.001	+26.4%								90 (18)	

Group	Fig	*Genotype*	Rep	Lifespan (mean ± s.e.)	vs. WT			vs. *his-71 germ-Tg*			Combined Lifespan (mean ± s.e.)	vs. WT	vs. *his-71 germ-Tg*	Total number (censored)	Lab Code Strain Number
					P Value	§Lifespan Change	Combined *p* Value	*P Value*	§Lifespan Change	Combined *p* Value		Combined Lifespan change	Combined Lifespan change		
1a	fig. S3J, S4U	*WT*	#1	16.501 ± 0.369							16.34 ± 0.21			90 (16)	N2
		WT	#2	16.180 ± 0.363										90 (22)	
		WT	#3	16.335 ± 0.378										90 (16)	
1b	fig. S3J	*his-71 germ-Tg*	#1	20.213 ± 0.593	<0.001	+22.5%	<0.001				19.84 ± 0.33	21%		90 (29)	MCW1321
		his-71 germ-Tg	#2	19.626 ± 0.563	<0.001	+21.3%								90 (30)	
		his-71 germ-Tg	#3	19.693 ± 0.577	<0.001	+20.6%								90 (28)	
1b	fig. S4U	*his-71K79A germ-Tg*	#1	17.832 ± 0.421	0.004	+8.1%	<0.001	<0.001	-11.8%	<0.001	17.78 ± 0.24	9%	-10%	90 (17)	MCW1527
		his-71K79A germ-Tg	#2	17.645 ± 0.423	0.002	+9.1%		0.004	-10.1%					90 (14)	
		his-71K79A germ-Tg	#3	17.863 ± 0.401	0.003	+9.4%		0.005	-9.3%					90 (16)	

Group	Fig	*Genotype*	Rep	Lifespan (mean ± s.e.)	*# P value 1*	§Lifespan Change 1	※Combined *p* Value 1				Combined Lifespan (mean ± s.e.)	Combined lifespan Change 1		Total number (censored)	Lab Code Strain Number
		lipl-4 Tg	#1	26.994 ± 0.834										90 (24)	MCW14
1a	fig. S4F, S4G	*lipl-4 Tg*	#2	25.543 ± 0.820							26.24 ± 0.47			90 (14)	
		lipl-4 Tg	#3	26.294 ± 0.794										90 (18)	
		lipl-4 Tg;dot-1.3(lf)	#1	21.530 ± 0.684	<0.001	-20.2%								90 (38)	MCW1072
1b	fig. S4F	*lipl-4 Tg;dot-1.3(lf)*	#2	21.517 ± 0.669	<0.001	-15.8%	<0.001				21.38 ± 0.39	-19%		90 (41)	
		lipl-4 Tg;dot-1.3(lf)	#3	21.077 ± 0.669	<0.001	-19.8%								90 (42)	
		lipl-4 Tg;dot-1.1(lf)	#1	25.076 ± 0.779	0.070	-7.1%								90 (31)	MCW1098
1b	fig. S4G	*lipl-4 Tg;dot-1.1(lf)*	#2	25.378 ± 0.771	0.647	-0.6%	0.220				25.41 ± 0.43	-3%		90 (33)	
		lipl-4 Tg;dot-1.1(lf)	#3	25.797 ± 0.706	0.356	-1.9%								90 (33)	

Group	Fig	*Genotype*	Rep	Lifespan (mean ± s.e.)	vs. *dot-1.3(lf);lipl-4 Tg; non Tg*			*vs. lipl-4 Tg*			Combined Lifespan (mean ± s.e.)	vs. *dot-1.3(lf);lipl-4 Tg;non Tg*	*vs. lipl-4 Tg*	Total number (censored)	Lab Code Strain Number
					P Value	§Lifespan Change	Combined *p* Value	*P Value*	§Lifespan Change	Combined *p* Value		Combined lifespan Change	Combined lifespan Change		
Germline rescue of dot-1.3 expression															
		dot-1.3(lf);lipl-4 Tg; non Tg	#1	20.749 ± 0.733										84 (22)	MCW1072
1a		*dot-1.3(lf);lipl-4 Tg; non Tg*	#2	21.261 ± 0.720							21.13 ± 0.42			83 (19)	
		dot-1.3(lf);lipl-4 Tg; non Tg	#3	21.373 ± 0.763										83 (24)	
	Fig. 4D	*dot-1.3(lf);lipl-4 Tg; dot-1.3 Tg*	#1	25.658 ± 0.682	<0.001	+23.7%		0.956	1%					93 (21)	MCW1155
1b		*dot-1.3(lf);lipl-4 Tg; dot-1.3 Tg*	#2	25.239 ± 0.773	<0.001	+18.7%	<0.001	0.344	4%	0.783	25.59 ± 0.41	21%	1%	93 (21)	
		dot-1.3(lf);lipl-4 Tg; dot-1.3 Tg	#3	25.838 ± 0.678	<0.001	+20.9%		0.614	-2%					93 (15)	
		lipl-4 Tg	#1	25.528 ± 0.752	<0.001	+23.0%								90 (21)	MCW14
1b		*lipl-4 Tg*	#2	24.347 ± 0.664	0.008	+14.5%	<0.001				25.37 ± 0.41	20%		90 (14)	
		lipl-4 Tg	#3	26.321 ± 0.699	<0.001	+23.2%								90 (21)	

Group	Fig	*Genotype*	Rep	Lifespan (mean ± s.e.)	vs. *dot-1.3(lf);lipl-4 Tg; non Tg*			vs. *lipl-4 Tg*			Combined Lifespan (mean ± s.e.)	vs. *dot-1.3(lf);lipl-4 Tg;non Tg*	vs. *lipl-4 Tg*	Total number (censored)	Lab Code Strain Number
					P Value	§Lifespan Change	Combined *p* Value	*P Value*	§Lifespan Change	Combined *p* Value		Combined lifespan Change	Combined lifespan Change		
Intestine rescue of dot-1.3 expression															
	fig. S4O	*dot-1.3(lf);lipl-4 Tg; non Tg*	#1	22.033 ± 0.743										87 (42)	MCW1072
1a		*dot-1.3(lf);lipl-4 Tg; non Tg*	#2	22.367 ± 0.752							22.26 ± 0.42			88 (41)	
		dot-1.3(lf);lipl-4 Tg; non Tg	#3	22.370 ± 0.716										88 (43)	
		dot-1.3(lf);lipl-4 Tg;dot-1.3 Tg	#1	21.203 ± 0.704	0.431	-3.8%		<0.001	-17%					96 (39)	MCW1228
1b		*dot-1.3(lf);lipl-4 Tg;dot-1.3 Tg*	#2	20.901 ± 0.706	0.121	-6.6%	0.362	0.001	-14%	<0.001	21.33 ± 0.42	-4%	-16%	90 (46)	
		dot-1.3(lf);lipl-4 Tg;dot-1.3 Tg	#3	21.931 ± 0.775	0.715	-2.0%		<0.001	-17%					87 (42)	
		lipl-4 Tg	#1	25.528 ± 0.752	0.002	+15.9%								90 (21)	MCW14
1b		*lipl-4 Tg*	#2	24.347 ± 0.664	0.060	+8.9%	<0.001				25.37 ± 0.41	14%		90 (14)	
		lipl-4 Tg	#3	26.321 ± 0.699	0.000	+17.7%								90 (21)	

Group	Fig	*Genotype*	Rep	Lifespan (mean ± s.e.)	*# P value 1*	§Lifespan Change 1	※Combined *p* Value 1				Combined Lifespan (mean ± s.e.)	Combined lifespan Change 1		Total number (censored)	Lab Code Strain Number
	Fig. 4E	*WT*	#1	17.676 ± 0.361										90 (26)	N2
1a		*WT*	#2	16.934 ± 0.391							17.29 ± 0.22			90 (23)	
		WT	#3	17.280 ± 0.384										90 (25)	
		dot-1.3 Tg	#1	20.741 ± 0.566	<0.001	+17.3%								90 (22)	MCW1370
1b		*dot-1.3 Tg*	#2	20.220 ± 0.568	<0.001	+19.4%	<0.001				20.66 ± 0.33	19%		90 (21)	
		dot-1.3 Tg	#3	21.016 ± 0.566	<0.001	+21.6%								90 (22)	

Group	Fig	*Genotype*	Rep	Lifespan (mean ± s.e.)	*# P value 1*	§Lifespan Change	※Combined *p* Value 1				Combined Lifespan (mean ± s.e.)	Combined lifespan Change 1		Total number (censored)	Lab Code Strain Number
1a	fig. S4T	*dot-1.3(lf)*	#1	17.797 ± 0.381							18.03 ± 0.21			90 (11)	VC2294
		dot-1.3(lf)	#2	18.383 ± 0.325										90 (5)	
		dot-1.3(lf)	#3	17.888 ± 0.372										90 (6)	
1b		*his-71 germ-Tg;dot-1.3(lf)*	#1	17.393 ± 0.508	0.842	-2.3%	0.329				17.05 ± 0.29	-5%		90 (19)	MCW1372
		his-71 germ-Tg;dot-1.3(lf)	#2	16.749 ± 0.471	0.061	-8.9%								90 (19)	
		his-71 germ-Tg;dot-1.3(lf)	#3	17.013 ± 0.530	0.614	-4.9%								90 (24)	

Group	Fig	*Genotype*	Rep	Lifespan (mean ± s.e.)	*# P value 1*	§Lifespan Change	※Combined *p* value 1				Combined Lifespan (mean ± s.e.)	Combined lifespan Change 1		Total number (censored)	Lab Code Strain Number
1a	fig. S4V	*H3.3(lf)*	#1	18.676 ± 0.382							18.77 ± 0.22			90 (15)	FAS43
		H3.3(lf)	#2	18.733 ± 0.393										90 (10)	
		H3.3(lf)	#3	18.901 ± 0.387										90 (14)	
1b		*dot-1.3 Tg;H3.3(lf)*	#1	18.062 ± 0.488	0.634	-3.3%	0.365				18.00 ± 0.27	-4%		90 (21)	MCW1406
		dot-1.3 Tg;H3.3(lf)	#2	17.891 ± 0.476	0.275	-4.5%								90 (23)	
		dot-1.3 Tg;H3.3(lf)	#3	18.032 ± 0.451	0.218	-4.6%								90 (25)	

Group	Fig	*Genotype*	Rep	Lifespan (mean ± s.e.)	*# P value 1*	§Lifespan Change	※Combined *p* value 1				Combined Lifespan (mean ± s.e.)	Combined lifespan Change 1		Total number (censored)	Lab Code Strain Number
1a	fig. S4W	*his-71(lf)*	#1	17.846 ± 0.334							18.09 ± 0.20			90 (15)	RB1781
		his-71(lf)	#2	18.199 ± 0.353										90 (14)	
		his-71(lf)	#3	18.213 ± 0.357										90 (14)	
1b		*dot-1.3 Tg;his-71(lf)*	#1	21.198 ± 0.640	<0.001	+18.8%	<0.001				20.82 ± 0.38	15%		90 (13)	MCW1371
		dot-1.3 Tg;his-71(lf)	#2	20.499 ± 0.669	<0.001	+12.6%								90 (16)	
		dot-1.3 Tg;his-71(lf)	#3	20.751 ± 0.670	<0.001	+13.9%								90 (10)	

Group	Fig	*Genotype*	Rep	Lifespan (mean ± s.e.)	*# P value 1*	§Lifespan Change	※Combined *p* value 1	*& P Value 2*	§Lifespan Change 2	※Combined *p* Value 2	Combined Lifespan (mean ± s.e.)	Combined lifespan Change 1	Combined lifespan Change 2	Total number (censored)	Lab Code Strain Number
1a	Fig. 5A, fig. S5A, fig. S5B	*WT*	#1	14.698 ± 0.339							14.49 ± 0.19			90 (34)	N2
		WT	#2	14.363 ± 0.336										90 (29)	
		WT	#3	14.437 ± 0.313										90 (28)	
1b		*his-71(lf)*	#1	13.886 ± 0.345	0.092	-5.5%	0.272				13.96 ± 0.21	-4%		90 (40)	RB1781
		his-71(lf)	#2	13.974 ± 0.387	0.529	-2.7%								90 (41)	
		his-71(lf)	#3	14.029 ± 0.362	0.469	-2.8%								90 (39)	
2a	Fig. 5A	*raga-1(lf)*	#1	22.562 ± 0.919				<0.001	+53.5%	<0.001	22.66 ± 0.46		+56%	90 (41)	VC222
		raga-1(lf)	#2	22.349 ± 0.737				<0.001	+55.6%					90 (28)	
		raga-1(lf)	#3	23.177 ± 0.772				<0.001	+60.5%					90 (31)	
2b		*raga-1(lf);his-71(lf)*	#1	14.602 ± 0.598	<0.001	-35.3%	<0.001	0.888	+5.2%	0.302	14.98 ± 0.35	-34%	+7%	90 (5)	MCW1293
		raga-1(lf);his-71(lf)	#2	15.001 ± 0.600	<0.001	-32.9%		0.278	+7.3%					90 (5)	
		raga-1(lf);his-71(lf)	#3	15.335 ± 0.629	<0.001	-33.8%		0.110	+9.3%					90 (7)	
3a	fig. S5A	*daf-2(lf)*	#1	45.247 ± 2.647				<0.001	+207.8%	<0.001	45.41 ± 1.50		+213%	90 (44)	CB1370
		daf-2(lf)	#2	45.190 ± 2.649				<0.001	+214.6%					90 (49)	
		daf-2(lf)	#3	45.752 ± 2.567				<0.001	+216.9%					90 (49)	
3b		*daf-2(lf);his-71(lf)*	#1	46.704 ± 1.515	0.545	+3.2%	0.376	<0.001	+236.3%	<0.001	45.82 ± 0.85	+1%	+228%	90 (15)	MCW1300
		daf-2(lf);his-71(lf)	#2	46.260 ± 1.438	0.357	+2.4%		<0.001	+231.0%					90 (13)	
		daf-2(lf);his-71(lf)	#3	44.508 ± 1.503	0.206	-2.7%		<0.001	+217.3%					90 (13)	
4a	fig. S5B	*eat-2(lf)*	#1	22.345 ± 0.840				<0.001	+52.0%	<0.001	22.04 ± 0.47		+52%	90 (32)	DA1116
		eat-2(lf)	#2	22.317 ± 0.879				<0.001	+55.4%					90 (33)	
		eat-2(lf)	#3	21.524 ± 0.741				<0.001	+49.1%					90 (25)	
4b		*eat-2(lf);his-71(lf)*	#1	23.861 ± 0.789	0.209	+6.8%	0.111	<0.001	+71.8%	<0.001	23.57 ± 0.44	+7%	+69%	90 (21)	MCW1301
		eat-2(lf);his-71(lf)	#2	23.614 ± 0.770	0.297	+5.8%		<0.001	+69.0%					90 (19)	
		eat-2(lf);his-71(lf)	#3	23.210 ± 0.747	0.091	+7.8%		<0.001	+65.4%					90 (23)	

Group	Fig	*Genotype*	Rep	Lifespan (mean ± s.e.)	*# P value 1*	§Lifespan Change	※Combined *p* value 1	*& P Value 2*	§Lifespan Change 2	※Combined *p* Value 2	Combined Lifespan (mean ± s.e.)	Combined lifespan Change 1	Combined lifespan Change 2	Total number (censored)	Lab Code Strain Number
	fig. S5C	*WT*	#1	18.101 ± 0.354										90 (16)	N2
1a		*WT*	#2	17.942 ± 0.382							18.02 ± 0.21			90 (15)	
		WT	#3	18.019 ± 0.340										90 (11)	
		his-71(lf)	#1	18.412 ± 0.419	0.316	+1.7%								90 (25)	RB1781
1b		*his-71(lf)*	#2	18.540 ± 0.467	0.146	+3.3%	0.200				18.46 ± 0.25	+2%		90 (21)	
		his-71(lf)	#3	18.422 ± 0.439	0.300	+2.2%								90 (20)	
		glp-1(lf)	#1	26.733 ± 0.643				<0.001	+47.7%					90 (0)	CB4037
2a		*glp-1(lf)*	#2	26.867 ± 0.661				<0.001	+49.7%	<0.001	26.73 ± 0.37		+48%	90 (0)	
		glp-1(lf)	#3	26.578 ± 0.606				<0.001	+47.5%					90 (0)	
		glp-1(lf);his-71(lf)	#1	25.132 ± 0.768	0.542	-6.0%		<0.001	+36.5%					90 (1)	MCW1302
2b		*glp-1(lf);his-71(lf)*	#2	24.529 ± 0.766	0.079	-8.7%	0.355	<0.001	+32.3%	<0.001	24.92 ± 0.45	-7%	+35%	90 (1)	
		glp-1(lf);his-71(lf)	#3	25.111 ± 0.790	0.842	-5.5%		<0.001	+36.3%					90 (0)	

Group	Fig	*Genotype*	Rep	Lifespan (mean ± s.e.)	*# P value 1*	§Lifespan Change	※Combined *p* value 1	*& P Value 2*	§Lifespan Change 2	※Combined *p* Value 2	Combined Lifespan (mean ± s.e.)	Combined lifespan Change 1	Combined lifespan Change 2	Total number (censored)	Lab Code Strain Number
	Fig. 5B	*WT*	#1	16.369 ± 0.373										90 (13)	N2
1a		*WT*	#2	16.761 ± 0.353							16.59 ± 0.21			90 (15)	
		WT	#3	16.644 ± 0.376										90 (14)	
		dot-1.3(lf)	#1	17.263 ± 0.460	0.061	+5.5%								90 (20)	VC2294
1b		*dot-1.3(lf)*	#2	17.100 ± 0.496	0.147	+2.0%	0.004				17.47 ± 0.26	+5%		90 (16)	
		dot-1.3(lf)	#3	18.038 ± 0.407	0.008	+8.4%								90 (15)	
		raga-1(lf)	#1	20.176 ± 0.835				<0.001	+23.3%					90 (38)	VC222
2a		*raga-1(lf)*	#2	20.514 ± 0.901				<0.001	+22.4%	<0.001	19.80 ± 0.49		+19%	90 (34)	
		raga-1(lf)	#3	19.213 ± 0.772				<0.001	+15.4%					90 (25)	
		raga-1(lf);dot-1.3(lf)	#1	17.394 ± 0.809	0.019	-13.8%		0.640	+0.8%					90 (40)	MCW1288
2b		*raga-1(lf);dot-1.3(lf)*	#2	16.933 ± 0.748	0.002	-17.5%	<0.001	0.942	-1.0%	0.772	17.02 ± 0.44	-14%	-3%	90 (40)	
		raga-1(lf);dot-1.3(lf)	#3	16.741 ± 0.727	0.020	-12.9%		0.320	-7.2%					90 (38)	

Group	Fig	*Genotype*	Rep	Lifespan (mean ± s.e.)	*# P value 1*	§Lifespan Change	※Combined *p* value 1	*& P Value 2*	§Lifespan Change 2	※Combined *p* Value 2	Combined Lifespan (mean ± s.e.)	Combined lifespan Change 1	Combined lifespan Change 2	Total number (censored)	Lab Code Strain Number
	fig. S5E, S5F	*WT*	#1	16.184 ± 0.338										90 (19)	N2
1a		*WT*	#2	16.590 ± 0.372							16.38 ± 0.21			90 (22)	
		WT	#3	16.384 ± 0.384										90 (25)	
		dot-1.3(lf)	#1	17.418 ± 0.404	0.011	+7.6%								90 (20)	VC2294
1b		*dot-1.3(lf)*	#2	17.532 ± 0.408	0.046	+5.7%	<0.001				17.48 ± 0.24	+7%		90 (17)	
		dot-1.3(lf)	#3	17.494 ± 0.434	0.025	+6.8%								90 (19)	
	fig. S5E	*daf-2(lf)*	#1	41.610 ± 1.729				<0.001	+157.1%					90 (30)	CB1370
2a		*daf-2(lf)*	#2	43.000 ± 1.622				<0.001	+159.2%	<0.001	42.98 ± 0.93		+162%	90 (24)	
		daf-2(lf)	#3	44.315 ± 1.464				<0.001	+170.5%					90 (30)	
		daf-2(lf);dot-1.3(lf)	#1	42.143 ± 2.135	0.576	+1.3%		<0.001	+142.0%					90 (40)	MCW1178
2b		*daf-2(lf);dot-1.3(lf)*	#2	39.797 ± 2.274	0.578	-7.4%	0.871	<0.001	+127.0%	<0.001	40.61 ± 1.25	-6%	+132%	90 (40)	
		daf-2(lf);dot-1.3(lf)	#3	39.970 ± 2.136	0.871	-9.8%		<0.001	+128.5%					90 (33)	
	fig. S5F	*eat-2(lf)*	#1	25.008 ± 0.848				<0.001	+54.5%					90 (30)	DA1116
3a		*eat-2(lf)*	#2	25.422 ± 0.867				<0.001	+53.2%	<0.001	25.37 ± 0.48		+55%	90 (22)	
		eat-2(lf)	#3	25.626 ± 0.817				<0.001	+56.4%					90 (26)	
		eat-2(lf);dot-1.3(lf)	#1	24.931 ± 0.727	0.714	-0.3%		<0.001	+43.1%					90 (26)	MCW1203
3b		*eat-2(lf);dot-1.3(lf)*	#2	24.858 ± 0.752	0.399	-2.2%	0.864	<0.001	+41.8%	<0.001	25.10 ± 0.45	-1%	+44%	100 (34)	
		eat-2(lf);dot-1.3(lf)	#3	25.575 ± 0.863	0.984	-0.2%		<0.001	+46.2%					90 (33)	

Group	Fig	*Genotype*	Rep	Lifespan (mean ± s.e.)	*# P value 1*	§Lifespan Change	※Combined *p* value 1	*& P Value 2*	§Lifespan Change 2	※Combined *p* Value 2	Combined Lifespan (mean ± s.e.)	Combined lifespan Change 1	Combined lifespan Change 2	Total number (censored)	Lab Code Strain Number
	fig. S5G	*WT*	#1	17.610 ± 0.380										90 (10)	N2
1a		*WT*	#2	18.236 ± 0.438							18.01 ± 0.24			90 (8)	
		WT	#3	18.177 ± 0.417										90 (11)	
		dot-1.3	#1	16.011 ± 0.435	0.017	-9.1%								90 (17)	VC2294
1b		*dot-1.3*	#2	16.036 ± 0.426	<0.001	-12.1%	<0.001				15.88 ± 0.25	-12%		90 (16)	
		dot-1.3	#3	15.584 ± 0.435	<0.001	-14.3%								90 (17)	
		glp-1(lf)	#1	21.798 ± 0.979				<0.001	+23.8%					90 (3)	CB4037
2a		*glp-1(lf)*	#2	22.427 ± 0.944				<0.001	+23.0%	<0.01	21.99 ± 0.56		+22%	90 (2)	
		glp-1(lf)	#3	21.740 ± 0.994				<0.001	+19.6%					90 (3)	
		glp-1(lf);dot-1.3(lf)	#1	22.855 ± 0.877	0.616	+4.8%		<0.001	+42.7%					90 (1)	MCW1177
2b		*glp-1(lf);dot-1.3(lf)*	#2	21.871 ± 0.877	0.773	-2.5%	0.703	<0.001	+36.4%	<0.01	21.86 ± 0.50	-1%	+38%	100 (2)	
		glp-1(lf);dot-1.3(lf)	#3	20.854 ± 0.836	0.313	-4.1%		<0.001	+33.8%					90 (2)	

Group	Fig	*Genotype*	Rep	Lifespan (mean ± s.e.)	*# P value 1*	§Lifespan Change	※Combined *p* value 1				Combined Lifespan (mean ± s.e.)	Combined lifespan Change 1	Combined lifespan Change 2	Total number (censored)	Lab Code Strain Number
1a	Fig. 5H, fig. S5M	*WT*	#1	16.261 ± 0.429							16.38 ± 0.26			90 (10)	N2
		WT	#2	16.382 ± 0.433										90 (10)	
		WT	#3	16.499 ± 0.477										90 (9)	
1b	Fig. 5H	*aak-2 lyso-Tg*	#1	23.472 ± 0.740	<0.001	+44.3%	<0.001				23.18 ± 0.40	42%		90 (7)	MCW1653
		aak-2 lyso-Tg	#2	23.515 ± 0.751	<0.001	+43.5%								90 (3)	
		aak-2 lyso-Tg	#3	22.565 ± 0.603	<0.001	+36.8%								90 (4)	
1b	fig. S5M	*wrmScarlet lyso-Tg*	#1	15.667 ± 0.461	0.384	-3.7%	0.421				15.79 ± 0.27	-4%		90 (21)	MCW1654
		wrmScarlet lyso-Tg	#2	15.971 ± 0.471	0.624	-2.5%								90 (17)	
		wrmScarlet lyso-Tg	#3	15.704 ± 0.486	0.206	-4.8%								90 (21)	

Group	Fig	*Genotype*	Rep	Lifespan (mean ± s.e.)	*# P value 1*	§Lifespan Change	※Combined *p* value 1				Combined Lifespan (mean ± s.e.)	Combined lifespan Change 1	Combined lifespan Change 2	Total number (censored)	Lab Code Strain Number
1a	fig. S5L	*WT*	#1	16.755 ± 0.461							16.42 ± 0.27			90 (17)	N2
		WT	#2	16.540 ± 0.456										90 (14)	
		WT	#3	15.936 ± 0.510										90 (21)	
1b		*aak-2 Tg*	#1	21.824 ± 0.454	<0.001	+30.3%	<0.001				21.54 ± 0.28	31%		90 (1)	WBM60
		aak-2 Tg	#2	21.952 ± 0.482	<0.001	+32.7%								90 (2)	
		aak-2 Tg	#3	20.841 ± 0.497	<0.001	+30.8%								90 (3)	

Group	Fig	*Genotype*	Rep	Lifespan (mean ± s.e.)	*# P value 1*	§Lifespan Change	※Combined *p* value 1				Combined Lifespan (mean ± s.e.)	Combined lifespan Change 1	Combined lifespan Change 2	Total number (censored)	Lab Code Strain Number
1a	Fig. 5L	*WT_fed*	#1	16.478 ± 0.406							16.24 ± 0.18			90 (4)	N2
		WT_fed	#2	16.146 ± 0.417										90 (6)	
		WT_fed	#3	16.403 ± 0.413										90 (5)	
		WT_fed	#4	16.161 ± 0.465										90 (13)	
		WT_fed	#5	16.445 ± 0.467										90 (19)	
		WT_fed	#6	15.880 ± 0.445										90 (19)	
1b		*WT_starvation(stv)*	#1	19.053 ± 0.404	<0.001	16%	<0.001				19.28 ± 0.19	+19%		90 (7)	N2
		WT_stv	#2	18.297 ± 0.454	0.001	13%								90 (10)	
		WT_stv	#3	18.212 ± 0.418	0.005	11%								90 (10)	
		WT_stv	#4	20.019 ± 0.462	<0.001	24%								90 (11)	
		WT_stv	#5	19.943 ± 0.510	<0.001	21%								90 (15)	
		WT_stv	#6	20.227 ± 0.480	<0.001	27%								90 (9)	

Group	Fig	*Genotype*	Rep	Lifespan (mean ± s.e.)	*# P value 1*	§Lifespan Change	※Combined *p* value 1				Combined Lifespan (mean ± s.e.)	Combined lifespan Change 1	Combined lifespan Change 2	Total number (censored)	Lab Code Strain Number
1a	Fig. 5M	*his-71(lf)_fed*	#1	17.188 ± 0.455							17.38 ± 0.26			90 (11)	RB1781
		his-71(lf)_fed	#2	17.543 ± 0.464										90 (12)	
		his-71(lf)_fed	#3	17.403 ± 0.456										90 (10)	
1b		*his-71(lf) _stv*	#1	18.289 ± 0.462	0.143	6%	0.188				18.35 ± 0.26	+6%		90 (11)	RB1781
		his-71(lf)_stv	#2	18.555 ± 0.432	0.263	6%								90 (15)	
		his-71(lf)_stv	#3	18.232 ± 0.439	0.333	5%								90 (10)	

Group	Fig	*Genotype*	Rep	Lifespan (mean ± s.e.)	*# P value 1*	§Lifespan Change	※Combined *p* value 1				Combined Lifespan (mean ± s.e.)	Combined lifespan Change 1	Combined lifespan Change 2	Total number (censored)	Lab Code Strain Number
1a	Fig. 5N	*dot-1.3(lf)_fed*	#1	17.264 ± 0.428							17.08 ± 0.24			90 (3)	VC2294
		dot-1.3(lf)_fed	#2	16.957 ± 0.444										90 (3)	
		dot-1.3(lf)_fed	#3	17.030 ± 0.393										90 (3)	
1b		*dot-1.3(lf)_stv*	#1	17.446 ± 0.532	0.616	1%	0.169				17.58 ± 0.32	+3%		90 (19)	VC2294
		dot-1.3(lf)_stv	#2	17.547 ± 0.576	0.217	3%								90 (12)	
		dot-1.3(lf)_stv	#3	17.738 ± 0.563	0.080	4%								90 (16)	

Group	Fig	*Genotype*	Rep	Lifespan (mean ± s.e.)	*# P value 1*	§Lifespan Change	※Combined *p* value 1				Combined Lifespan (mean ± s.e.)	Combined lifespan Change 1	Combined lifespan Change 2	Total number (censored)	Lab Code Strain Number
1a	fig. S5R	*lipl-4(lf)_fed*	#1	18.274 ± 0.422							18.02 ± 0.25			90 (5)	tm4417
		lipl-4(lf)_fed	#2	18.010 ± 0.423										90 (3)	
		lipl-4(lf)_fed	#3	17.771 ± 0.438										90 (8)	
1b		*lipl-4(lf)_stv*	#1	19.693 ± 0.506	0.007	8%	<0.001				19.80 ± 0.27	+10%		90 (3)	tm4417
		lipl-4(lf)_stv	#2	19.963 ± 0.457	0.005	11%								90 (2)	
		lipl-4(lf)_stv	#3	19.757 ± 0.439	0.015	11%								90 (3)	

\# *P* value 1: compare values between the different alphabet initiated with the same number and followed by the same replicate numbers(#) in the same frame using a log-rank test, e.g. "1b#1 vs. 1a#1 ", "2b#3 vs. 2a#3".

& *P* value 2: compare value to the same alphabet initiated with No.1 and followed by the same replicate number (#) using a log-rank test, e.g. "2a#1 vs. 1a#1", "3a#2 vs. 1a#2", or "4b#3 vs. 1b#3".

§ Lifespan Changes (1, 2) are respectively attached to the *p* value analyses (1, 2) by using "+" to indicate the increased lifespan percentage and using "-" to indicate the decreased lifespan percentage.

※ Combined *p* value of three independent replicates is calculated by the Fisher's method using the R package metap (v1.8).
