# Supplementary material for: Lysosomes Signal through the Epigenome to Regulate Longevity across Generations
Source: Science. Author manuscript; Available in PMC 2026 Jan 24. (PMC12831228; doi:10.1126/science.adn8754)
Supplement: Table S4_20250204 [file NIHMS2127653-supplement-Table_S4_20250204.pdf]

Table S4. Summary of longitudinal survival analyses using RNAi treatments.

Group	Fig	*Genotype*	RNAi	Rep	Lifespan (mean ± s.e.)	# *P* value 1	§Lifespan Change	※ Combined *p* value 1	& *P* value 2	§Lifespan Change	※ Combined *p* value 2	Combined Lifespan (mean ± s.e.)	Combined lifespan Change 1	Combined lifespan Change 2	Total number (censored)	Lab Code Strain Number
1a	Fig. 4A	*WT*	*EV*	#1	16.857 ± 0.587							16.55 ± 0.32			63 (9)	N2
		WT	*EV*	#2	16.302 ± 0.544										63 (9)	
		WT	*EV*	#3	16.497 ± 0.550										64 (6)	
1b		*lipl-4 Tg*	*EV*	#1	24.448 ± 0.435	<0.001	+45.0%	<0.001				24.39 ± 0.30	+47%		70 (12)	MCW14
		lipl-4 Tg	*EV*	#2	24.597 ± 0.522	<0.001	+50.9%								70 (17)	
		lipl-4 Tg	*EV*	#3	24.136 ± 0.595	<0.001	+46.3%								70 (13)	
2a		*WT*	*dot-1.3*	#1	18.540 ± 0.550				0.096	+10.0%	<0.001	18.76 ± 0.33		+13%	63 (8)	N2
		WT	*dot-1.3*	#2	18.340 ± 0.590				0.015	+12.5%					63 (7)	
		WT	*dot-1.3*	#3	19.400 ± 0.570				0.002	+17.6%					62 (7)	
2b		*lipl-4 Tg*	*dot-1.3*	#1	21.269 ± 0.592	<0.001	+14.7%	<0.001	<0.001	-13.0%	<0.001	21.81 ± 0.33	+16%	-11%	70 (18)	MCW14
		lipl-4 Tg	*dot-1.3*	#2	21.946 ± 0.502	<0.001	+19.7%		<0.001	-10.8%					70 (23)	
		lipl-4 Tg	*dot-1.3*	#3	22.236 ± 0.588	<0.001	+14.6%		0.017	-7.9%					70 (19)	

Group	Fig	*Genotype*	RNAi	Rep	Lifespan (mean ± s.e.)	# *P* value 1	§Lifespan Change	※ Combined *p* value 1	& *P* value 2	§Lifespan Change	※ Combined *p* value 2	Combined Lifespan (mean ± s.e.)	Combined lifespan Change 1	Combined lifespan Change 2	Total number (censored)	Lab Code Strain Number
1a	fig. S4A, S4C, S4E	*WT*	*EV*	#1	17.275 ± 0.556							17.31 ± 0.32			80 (9)	N2
		WT	*EV*	#2	17.132 ± 0.556										80 (9)	
		WT	*EV*	#3	17.547 ± 0.560										80 (12)	
1b		*lipl-4 Tg*	*EV*	#1	23.769 ± 0.386	<0.001	+37.6%	<0.001				23.67 ± 0.27	+37%		90 (7)	MCW14
		lipl-4 Tg	*EV*	#2	23.645 ± 0.520	<0.001	+38.0%								80 (14)	
		lipl-4 Tg	*EV*	#3	23.574 ± 0.500	<0.001	+34.3%								82 (7)	
2a	fig. S4A	*WT*	*dot-1.1*	#1	22.956 ± 0.603				<0.001	+32.9%	<0.001	22.75 ± 0.37		+31%	80 (6)	N2
		WT	*dot-1.1*	#2	22.653 ± 0.673				<0.001	+32.2%					80 (5)	
		WT	*dot-1.1*	#3	22.642 ± 0.644				<0.001	+29.0%					80 (3)	
2b		*lipl-4 Tg*	*dot-1.1*	#1	27.009 ± 0.632	<0.001	+17.7%	<0.001	<0.001	+13.6%	<0.001	26.63 ± 0.35	+17%	+13%	80 (18)	MCW14
		lipl-4 Tg	*dot-1.1*	#2	26.496 ± 0.633	<0.001	+17.0%		<0.001	+12.1%					80 (14)	
		lipl-4 Tg	*dot-1.1*	#3	26.395 ± 0.584	0.001	+16.6%		<0.001	+12.0%					80 (16)	
3a	fig. S4C	*WT*	*dot-1.4*	#1	18.305 ± 0.522				0.255	+6.0%	0.143	18.31 ± 0.33		+6%	80 (5)	N2
		WT	*dot-1.4*	#2	17.960 ± 0.607				0.227	+4.8%					80 (7)	
		WT	*dot-1.4*	#3	18.650 ± 0.589				0.143	+6.3%					80 (6)	
3b		*lipl-4 Tg*	*dot-1.4*	#1	24.417 ± 0.362	<0.001	+33.4%	<0.001	0.332	+2.7%	0.174	24.74 ± 0.23	+35%	+5%	80 (8)	MCW14
		lipl-4 Tg	*dot-1.4*	#2	25.119 ± 0.454	<0.001	+39.9%		0.118	+6.2%					72 (13)	
		lipl-4 Tg	*dot-1.4*	#3	24.743 ± 0.389	<0.001	+32.7%		0.285	+5.0%					80 (10)	
4a	fig. S4E	*WT*	*D1053.2*	#1	18.170 ± 0.510				0.382	+5.2%	0.797	17.82 ± 0.29		+3%	80 (7)	N2
		WT	*D1053.2*	#2	17.710 ± 0.490				0.678	+3.4%					80 (5)	
		WT	*D1053.2*	#3	17.600 ± 0.490				0.823	+0.3%					80 (5)	
4b		*lipl-4 Tg*	*D1053.2*	#1	26.580 ± 0.470	<0.001	+46.3%	<0.001	<0.001	+11.8%	<0.001	26.81 ± 0.27	+50%	+13%	80 (12)	MCW14
		lipl-4 Tg	*D1053.2*	#2	26.970 ± 0.470	<0.001	+52.3%		<0.001	+14.1%					80 (9)	
		lipl-4 Tg	*D1053.2*	#3	26.870 ± 0.480	<0.001	+52.7%		<0.001	+14.0%					80 (11)	

Group	Fig	*Genotype*	RNAi	Rep	Lifespan (mean ± s.e.)	# *P* value 1	§Lifespan Change	※ Combined *p* value 1	& *P* value 2	§Lifespan Change	※ Combined *p* value 2	Combined Lifespan (mean ± s.e.)	Combined lifespan Change 1	Combined lifespan Change 2	Total number (censored)	Lab Code Strain Number
1a	fig. S4B , S4D	*WT*	*EV*	#1	16.120 ± 0.535							16.12 ± 0.33			76 (3)	N2
		WT	*EV*	#2	15.680 ± 0.653										66 (5)	
		WT	*EV*	#3	16.490 ± 0.568										83 (7)	
1b		*lipl-4 Tg*	*EV*	#1	22.979 ± 0.469	<0.001	+42.5%	<0.001				23.17 ± 0.27	+44%		84 (11)	MCW14
		lipl-4 Tg	*EV*	#2	23.581 ± 0.486	<0.001	+50.4%								84 (23)	
		lipl-4 Tg	*EV*	#3	22.987 ± 0.460	<0.001	+39.4%								84 (17)	
2a	fig. S4B	*WT*	*dot-1.2*	#1	17.297 ± 0.538				0.167	+7.3%	0.266	17.16 ± 0.30		+6%	83 (11)	N2
		WT	*dot-1.2*	#2	17.114 ± 0.505				0.223	+9.1%					79 (8)	
		WT	*dot-1.2*	#3	17.074 ± 0.507				0.589	+3.5%					84 (8)	
2b		*lipl-4 Tg*	*dot-1.2*	#1	24.916 ± 0.435	<0.001	+44.0%	<0.001	<0.001	+8.4%	0.010	24.52 ± 0.26	+43%	+6%	83 (9)	MCW14
		lipl-4 Tg	*dot-1.2*	#2	24.352 ± 0.444	<0.001	+42.3%		0.117	+3.3%					85 (14)	
		lipl-4 Tg	*dot-1.2*	#3	24.277 ± 0.458	<0.001	+42.2%		0.004	+5.6%					84 (16)	
3a	fig. S4D	*WT*	*dot-1.5*	#1	17.239 ± 0.567				0.256	+6.9%	0.256	17.35 ± 0.29		+8%	82 (15)	N2
		WT	*dot-1.5*	#2	17.172 ± 0.439				0.257	+9.5%					82 (12)	
		WT	*dot-1.5*	#3	17.629 ± 0.522				0.313	+6.9%					82 (10)	
3b		*lipl-4 Tg*	*dot-1.5*	#1	22.419 ± 0.459	<0.001	+30.0%	<0.001	0.416	-2.4%	0.672	22.67 ± 0.27	+31%	-2%	82 (11)	MCW14
		lipl-4 Tg	*dot-1.5*	#2	22.703 ± 0.483	<0.001	+32.2%		0.335	-3.7%					82 (8)	
		lipl-4 Tg	*dot-1.5*	#3	22.893 ± 0.494	<0.001	+29.9%		0.956	-0.4%					82 (10)	

Group	Fig	*Genotype*	RNAi	Rep	Lifespan (mean ± s.e.)	# *P* value 1	§Lifespan Change	※ Combined *p* value 1	& *P* value 2	§Lifespan Change	※ Combined *p* value 2	Combined Lifespan (mean ± s.e.)	Combined lifespan Change 1	Combined lifespan Change 2	Total number (censored)	Lab Code Strain Number
RNAi defective control																
1a	fig. S4K	*lipl-4 Tg*	*EV*	#1	20.955 ± 0.458							20.70 ± 0.28			92 (22)	MCW1564
		lipl-4 Tg	*EV*	#2	20.540 ± 0.498										91 (30)	
		lipl-4 Tg	*EV*	#3	20.584 ± 0.511										92 (30)	
1b		*lipl-4 Tg*	*dot-1.3*	#1	21.569 ± 0.639	0.183	+2.9%	0.004				21.99 ± 0.34	+6%		90 (28)	MCW1564
		lipl-4 Tg	*dot-1.3*	#2	22.265 ± 0.548	0.017	+8.4%								91 (24)	
		lipl-4 Tg	*dot-1.3*	#3	22.129 ± 0.592	0.026	+7.5%								91 (26)	

Group	Fig	*Genotype*	RNAi	Rep	Lifespan (mean ± s.e.)	vs. *lipl-4 Tg with EV treatment*			vs. *lipl-4 Tg with dot-1.3 RNAi treatment*			Combined Lifespan (mean ± s.e.)	vs. *lipl-4 Tg with EV treatment*	vs. *lipl-4 Tg with dot-1.3 RNAi treatment*	Total number (censored)	Lab Code Strain Number
						# *P* value	§Lifespan Change	※ Combined *p* value	& *P* value	§Lifespan Change	※ Combined *p* value		Combined lifespan Change	Combined lifespan Change		
Germline-specific knockdown																
		lipl-4 Tg	*EV*	#1	21.292 ± 0.458										90 (18)	MCW1057
1a		*lipl-4 Tg*	*EV*	#2	21.552 ± 0.508							21.29 ± 0.45			90 (19)	
		lipl-4 Tg	*EV*	#3	22.006 ± 0.503										90 (26)	
		lipl-4 Tg	*dot-1.3*	#1	19.006 ± 0.489	0.001	-10.7%								90 (29)	
1b	Fig. 4C	*lipl-4 Tg*	*dot-1.3*	#2	19.689 ± 0.531	0.010	-8.6%	<0.001				19.01 ± 0.48	-11%		90 (28)	
		lipl-4 Tg	*dot-1.3*	#3	19.009 ± 0.527	<0.001	-13.6%								90 (34)	
		non Tg	*EV*	#1	15.273 ± 0.480	<0.001	-28.3%		<0.001	-19.6%					90 (24)	AMJ345
1b		*non Tg*	*EV*	#2	15.502 ± 0.389	<0.001	-28.1%	<0.001	<0.001	-21.3%	<0.001	15.27 ± 0.48	-28%	-20%	90 (16)	
		non Tg	*EV*	#3	15.381 ± 0.463	<0.001	-30.1%		<0.001	-19.1%					90 (22)	

`

Group	Fig	*Genotype*	RNAi	Rep	Lifespan (mean ± s.e.)	vs. *lipl-4 Tg with EV treatment*			vs. *lipl-4 Tg with dot-1.3 RNAi treatment*			Combined Lifespan (mean ± s.e.)	vs. *lipl-4 Tg with EV treatment*	vs. *lipl-4 Tg with dot-1.3 RNAi treatment*	Total number (censored)	Lab Code Strain Number
						# *P* value	§Lifespan Change	※ Combined *p* value	& *P* value	§Lifespan Change	※ Combined *p* value		Combined lifespan Change	Combined lifespan Change		
Intestine-specific knockdown																
		lipl-4 Tg	*EV*	#1	26.435 ± 0.614										90 (11)	MCW1056
1a		*lipl-4 Tg*	*EV*	#2	25.752 ± 0.580							26.49 ± 0.34			90 (11)	
		lipl-4 Tg	*EV*	#3	27.290 ± 0.583										90 (12)	
		lipl-4 Tg	*dot-1.3*	#1	26.964 ± 0.586	0.579	+2.0%								90 (8)	
1b	fig. S4N	*lipl-4 Tg*	*dot-1.3*	#2	26.642 ± 0.551	0.283	+3.5%	0.684				26.86 ± 0.33	+1%		90 (5)	
		lipl-4 Tg	*dot-1.3*	#3	26.999 ± 0.585	0.848	-1.1%								90 (9)	
		non Tg	*EV*	#1	19.473 ± 0.478	<0.001	-27.8%		<0.001	-27.8%					90 (6)	JM45
1b		*non Tg*	*EV*	#2	19.051 ± 0.477	<0.001	-28.5%	<0.001	<0.001	-28.5%	<0.001	19.35 ± 0.27	-27%	-28%	90 (6)	
		non Tg	*EV*	#3	19.519 ± 0.451	<0.001	-27.7%		<0.001	-27.7%					90 (6)	

Group	Fig	*Genotype*	RNAi	Rep	Lifespan (mean ± s.e.)	# *P* value 1	§Lifespan Change	※ Combined *p* value 1	& *P* value 2	§Lifespan Change	※ Combined *p* value 2	Combined Lifespan (mean ± s.e.)	Combined lifespan Change 1	Combined lifespan Change 2	Total number (censored)	Lab Code Strain Number
		WT	*EV*	#1	17.394 ± 0.643										90 (3)	N2
1a		*WT*	*EV*	#2	17.719 ± 0.613							17.59 ± 0.36			90 (1)	
		WT	*EV*	#3	17.654 ± 0.651										90 (4)	
		his-71(lf)	*EV*	#1	17.804 ± 0.630	0.758	+2.4%								90 (3)	RB1781
1b		*his-71(lf)*	*EV*	#2	17.689 ± 0.632	0.983	-0.2%	0.941				17.55 ± 0.36	0%		90 (3)	
	fig. S5D	*his-71(lf)*	*EV*	#3	17.167 ± 0.608	0.560	-2.8%								90 (3)	
		WT	*cco-1*	#1	30.632 ± 1.092				<0.001	+76.1%					90 (32)	N2
2a		*WT*	*cco-1*	#2	30.719 ± 1.180				<0.001	+73.4%	<0.001	30.13 ± 0.64		71%	90 (38)	
		WT	*cco-1*	#3	29.022 ± 1.061				<0.001	+64.4%					90 (36)	
		his-71(lf)	*cco-1*	#1	31.185 ± 0.928	0.999	+1.8%		<0.001	+75.2%					90 (23)	RB1781
2b		*his-71(lf)*	*cco-1*	#2	31.362 ± 1.027	0.886	+2.1%	0.426	<0.001	+77.3%	<0.001	31.55 ± 0.54	5%	80%	90 (27)	
		his-71(lf)	*cco-1*	#3	32.088 ± 0.894	0.057	+10.6%		<0.001	+86.9%					90 (21)	

Group	Fig	*Genotype*	RNAi	Rep	Lifespan (mean ± s.e.)	# *P* value 1	§Lifespan Change	※ Combined *p* value 1	& *P* value 2	§Lifespan Change	※ Combined *p* value 2	Combined Lifespan (mean ± s.e.)	Combined lifespan Change 1	Combined lifespan Change 2	Total number (censored)	Lab Code Strain Number
		WT	*EV*	#1	15.241 ± 0.446										90 (3)	N2
1a		*WT*	*EV*	#2	14.956 ± 0.422							15.27 ± 0.32			90 (4)	
		WT	*EV*	#3	15.243 ± 0.438										90 (3)	
		dot-1.3(lf)	*EV*	#1	11.014 ± 0.266	<0.001	-27.7%								90 (21)	VC2294
1b		*dot-1.3(lf)*	*EV*	#2	11.178 ± 0.284	<0.001	-25.3%	<0.001				11.10 ± 0.19	-27%		90 (17)	
	fig. S5H	*dot-1.3(lf)*	*EV*	#3	11.545 ± 0.311	<0.001	-24.3%								90 (24)	
		WT	*cco-1*	#1	32.047 ± 1.016				<0.001	+110.3%					90 (27)	N2
2a		*WT*	*cco-1*	#2	30.904 ± 1.128				<0.001	+106.6%	<0.001	31.63 ± 0.70		+107%	90 (28)	
		WT	*cco-1*	#3	31.550 ± 1.123				<0.001	+107.0%					90 (28)	
		dot-1.3(lf)	*cco-1*	#1	26.731 ± 0.960	0.001	-16.6%		<0.001	+142.7%					90 (23)	VC2294
2b		*dot-1.3(lf)*	*cco-1*	#2	27.247 ± 1.089	0.037	-11.8%	<0.001	<0.001	+143.8%	<0.001	26.62 ± 0.73	-16%	+140%	90 (28)	
		dot-1.3(lf)	*cco-1*	#3	27.864 ± 1.074	0.019	-11.7%		<0.001	+141.4%					90 (31)	

Group	Fig	*Genotype*	RNAi	Rep	Lifespan (mean ± s.e.)	# *P* value 1	§Lifespan Change	※ Combined *p* value 1	& *P* value 2	§Lifespan Change	※ Combined *p* value 2	Combined Lifespan (mean ± s.e.)	Combined lifespan Change 1	Combined lifespan Change 2	Total number (censored)	Lab Code Strain Number
1a	fig. S5K	*WT*	*EV*	#1	14.438 ± 0.452							14.39 ± 0.27			90 (1)	N2
		WT	*EV*	#2	14.231 ± 0.448										90 (2)	
		WT	*EV*	#3	14.502 ± 0.493										90 (3)	
1b		*lipl-4 Tg*	*EV*	#1	28.023 ± 0.458	<0.001	+94.1%	<0.001				28.14 ± 0.26	96%		90 (3)	MCW14
		lipl-4 Tg	*EV*	#2	28.205 ± 0.448	<0.001	+98.2%								90 (4)	
		lipl-4 Tg	*EV*	#3	28.195 ± 0.451	<0.001	+94.4%								90 (5)	
2a		*WT*	*raga-1*	#1	18.772 ± 0.533				<0.001	+30.0%	<0.001	18.64 ± 0.30		30%	90 (1)	N2
		WT	*raga-1*	#2	18.590 ± 0.512				<0.001	+30.6%					90 (1)	
		WT	*raga-1*	#3	18.560 ± 0.514				<0.001	+28.0%					90 (2)	
2b		*lipl-4 Tg*	*raga-1*	#1	26.588 ± 0.655	<0.001	+41.6%	<0.001	0.433	-5.1%	0.099	26.27 ± 0.37	41%	-7%	90 (11)	MCW14
		lipl-4 Tg	*raga-1*	#2	26.371 ± 0.624	<0.001	+41.9%		0.117	-6.5%					90 (8)	
		lipl-4 Tg	*raga-1*	#3	25.869 ± 0.667	<0.001	+39.4%		0.095	-8.2%					90 (8)	

P value 1: compare values between the different alphabet initiated with the same number and followed by the same replicate numbers(#) in the same frame using a log-rank test, e.g. "1b#1 vs. 1a#1 ", "2b#3 vs. 2a#3".

& *P* value 2: compare value to the same alphabet initiated with No.1 and followed by the same replicate number (#) using a log-rank test, e.g. "2a#1 vs. 1a#1", "3a#2 vs. 1a#2", or "4b#3 vs. 1b#3".

※ Combined *p* value of three independent replicates is calculated by the Fisher's method using the R package metap(v1.8).

§ Lifespan Changes (1, 2) are respectively attached to the *p* value analyses (1, 2) by using "+" to indicate the increased lifespan percentage and using "-" to indicate the decreased lifespan percentage.
