# Supplementary material for: Lysosomes Signal through the Epigenome to Regulate Longevity across Generations
Source: Science. Author manuscript; Available in PMC 2026 Jan 24. (PMC12831228; doi:10.1126/science.adn8754)
Supplement: Table S5_primers_20250204 [file NIHMS2127653-supplement-Table_S5_primers_20250204.pdf]

Table S5. List of primer sequences.

**Primer name**		**Primer sequence**	**Experimental assays**	**Notes**
rpl-32	FWD	AGGGAATTGATAACCGTGTCCGCA	qRT-PCR	
rpl-32	REV	TGTAGGACTGCATGAGGAGCATGT	qRT-PCR	
his-69	FWD	ACGAAAGCTGCTCGAAAGAAC	qRT-PCR	
his-69	REV	GATTGGAAGCGAAGATCCTGT	qRT-PCR	
his-70	FWD	AAAGGACTCAGTTTTTTTGAGAACA	qRT-PCR	
his-70	REV	AATTTCCTTGAGTGCATTGGAAC	qRT-PCR	
his-71	FWD	CCCGTGAACCAGAATTGAAATG	qRT-PCR	
his-71	REV	TTGCGAGCCGCCTTAGTG	qRT-PCR	
his-72	FWD	ACCATAATCCTCGTATTTTTCCAT	qRT-PCR	
his-72	REV	GTTGGAGTTCAACATGAAATTCAG	qRT-PCR	
his-74	FWD	TATGCCTCTTGATGAAATTCCG	qRT-PCR	
his-74	REV	GAAAAAGGAACGCAGGGTTTAT	qRT-PCR	
sun-1	FWD	CGTGCGACCTTGACCGTG	qRT-PCR	
sun-1	REV	GTCCGTGCATGCCACGAC	qRT-PCR	
vha-6	FWD	ATGGAGAGAAAGATTAATTTCGTCG	qRT-PCR	
vha-6	REV	AGCTTCCGAGATTGACATAGCAG	qRT-PCR	
univeral canonical H3	FWD	CCATTCCAGCGCCTTGTT	qRT-PCR	
univeral canonical H3	REV	TCCTGAAGAGCCATGACAGC	qRT-PCR	
his-71-3xflag	FWD	ACAAGGATGACGATGACAAGAG	qRT-PCR	for detection of *his-71* expressed from intestinal *his-71-3xFlag* transgene
his-71-3xflag	REV	CACAGGGAGAAAGAGCATGTAG	qRT-PCR	for detection of *his-71* expressed from intestinal *his-71-3xFlag* transgene
lipl-4	FWD	ATGGCCGAGAAGTTCCTACATCGT	qRT-PCR	
lipl-4	REV	GGTGAATTGGCGACCCAATCGAAA	qRT-PCR	
glp-1(e2141)	FWD	CCTCTCCATGTTCAAGCTGC	Genotyping by DNA sequencing	point mutation

glp-1(e2141)	REV	CATTTCATGGTTAAACATGTACTCC	Genotyping by DNA sequencing	point mutation
daf-2(e1370)	FWD	CCTTCTCGCTGCTTCGCTTTC	Genotyping by DNA sequencing	point mutation
daf-2(e1370)	REV	GCGTACCTGGAGTCGCTCAAG	Genotyping by DNA sequencing	point mutation
eat-2(ad1116)	FWD	CACATGCATTTGAAACGGAGAAC	Genotyping by DNA sequencing	point mutation
eat-2(ad1116)	REV	CTCACCCAGTCGCCCATCC	Genotyping by DNA sequencing	point mutation
rde-1(ne219)	FWD	ACCTTTCCTGGACATTCGATCA	Genotyping by DNA sequencing	point mutation
rde-1(ne219)	REV	TTTTCAGGTCGAGATTGACAGAAC	Genotyping by DNA sequencing	point mutation
jamSi2 [Pmex-5::rde-1(+)]	FWD	CCGTACTCCGTTTGTTTGATC	Genotyping	knockin. Cloning Primer source: doi: 10.1073/pnas.1608959113
jamSi2 [Pmex-5::rde-1(+)]	REV	TGCCGTCGCATTTACCAGTG	Genotyping	knockin. Cloning Primer source: doi: 10.1073/pnas.1608959113
dot-1.3(2831)	FWD	TATTGAGGAGGAGGGAAGGG	Genotyping	deletion mutation
dot-1.3(2831)	REV	TTTTCTGGACAAACGCTGAG	Genotyping	deletion mutation
dot-1.1(ok2154)-MU	FWD	GAAAATTGAAAATTCCGCCA	Genotyping	deletion mutation
dot-1.1(ok2154)-MU	REV	AGAACAAAGGTGGTGGATGC	Genotyping	deletion mutation
dot-1.1(ok2154)-WT	REV	CATGGTGCATCGTCAAG	Genotyping	work with dot-1.1(ok2154)-MU-FWD to produce PCR amplicon only from WT or hets
raga-1(ok386)-MU	FWD	AACTACATCGCCAGGGATTG	Genotyping	deletion mutation
raga-1(ok386)-MU	REV	TTCGAAGAATCGTGTGGTCA	Genotyping	deletion mutation
raga-1(ok386)-WT	FWD	TGGGATGAGACACTCTACAAAGC	Genotyping	produce PCR amplicon only from WT or hets
raga-1(ok386)-WT	REV	AAATCTAAGCGGTGAATTACGAA	Genotyping	produce PCR amplicon only from WT or hets
his-69&70(uge44)	FWD	GCTGGCTCTTTGTCGGTCTC	Genotyping	deletion mutation
his-69&70(uge44)	REV	CATTGAAATCACCGGAACCAC	Genotyping	deletion mutation
his-71(ok2289)-MU	FWD	TGTTCCCGTTCACAATCGTA	Genotyping	deletion mutation
his-71(ok2289)-MU	REV	AAACTCAAAACCGGCAAATG	Genotyping	deletion mutation
his-71(ok2289)-WT	FWD	TCGGTCTTGAAATCTTGAGCTAGA	Genotyping	produce PCR amplicon only from WT or hets
his-71(ok2289)-WT	REV	CGTCTCCGCATAAACACCA	Genotyping	produce PCR amplicon only from WT or hets

dot-1.3::mNeonGreen::3xFlag	FWD	GCTCAAAGAGATCCTCATGGGCTGCAAG	Genotyping by DNA sequencing	knockin
dot-1.3::mNeonGreen::3xFlag	REV	TGAGGAGGAGGGAAGGGGGAGATC	Genotyping by DNA sequencing	knockin
his-71::mNeonGreen::3xFlag	FWD	CGCAAGCTTCCATTCCAACGTCT	Genotyping by DNA sequencing	knockin
his-71::mNeonGreen::3xFlag	REV	GGCGCGCTTTATAGTTGTTAGCCT	Genotyping by DNA sequencing	knockin
dot-1.3-codingsequence	FWD	ATGCCAGTCATTCACTTGTCAAG	Cloning	
dot-1.3-codingsequence	REV	TCACTTGTTGCGATTGATTGTAG	Cloning	
his-71 cDNA	FWD	ATAATTTTCAGGTGAACCAGAATTGA	Cloning	
his-71 cDNA	REV	CACAAAATATTTACGTTTATTACTTTTTTGTAA	Cloning	
HIS-71K79A-mu	FWD	TCAAGATTTCgcaACCGATCTCCGTTTC	Cloning	Q5® Site-Directed Mutagenesis (mutations in red)
HIS-71K79A-mu	REV	GCGATTTCACGAACAAGAC	Cloning	
PDD162-gR-dot-1.3	FWD	gactacaatcaatcgcaacaGTTTTAGAGCTAGAAATAGCAAGT	Cloning	Cas9–sgRNA construct for generating DOT-1.3::mNeonGreen::3xFlag (gRNA sequence is in lower case)
PDD162-gR-his-71	FWD	acgtttatgcacgttctccaGTTTTAGAGCTAGAAATAGCAAGT	Cloning	Cas9–sgRNA construct for generating HIS-71::mNeonGreen::AID (gRNA sequence is in lower case)
PDD162	REV	CAAGACATCTCGCAATAGG	Cloning	univeral reverse primer for Cas9–sgRNA construct
his-71-5'-homology arm	FWD	acgttgtaaaacgacggccagtcgccggcaCTGGCGGAGCGTGCCG	Cloning	*his-71* homolgy arm adding to mNeonGreen-SEC vector (PDD268). Overlapping sequence to vector PDD268 is in lower case.
his-71-5'-homology arm	REV	catcgatgctcctgaggctcccgatgctccTGCACGTTCTCCACGTATGCGT	Cloning	*his-71* homolgy arm adding to mNeonGreen-SEC vector (PDD268). Overlapping sequence to vector PDD268 is in lower case. Synonymous mutation in PAM is labbled in red.
his-71-3'-homology arm	FWD	cgtgattacaaggatgacgatgacaagagaTAAACGTTGAGCTGTTTCGCCA	Cloning	*his-71* homolgy arm adding to mNeonGreen-SEC vector (PDD268). Overlapping sequence to vector PDD268 is in lower case.
his-71-3'-homology arm	REV	ggaaacagctatgaccatgttatcgatttcCTAGCACTCCCTTTTCAGTTTCGTT	Cloning	*his-71* homolgy arm adding to mNeonGreen-SEC vector (PDD268). Overlapping sequence to vector PDD268 is in lower case.
auxin-inducible degron (AID) minimal sequence	Fragment	attttcagggagccggatctgattacggatccggaggtggcgggATGCCTAAAGATCCAGCCAAACCTCCGGCCAAGGCACAAGTTGTGGGATGGCCACCGGTGAGATCATACCGGAAGAACGTGATGGTTTCCTGCCAAAAATCAAGCGGTGGCCCGGAGGCGGCGGCGTTCGTGAAGtaaacgttgagctgtttcgccaat	Cloning	Sequence source: doi: 10.1093/genetics/iyab006. Overlappiong sequences to recombinant vector (PDD268) with his-71 homology arms are in lower case.

dot-1.3-5'-homology arm	FWD	acgttgtaaaacgacggccagtcgccggcaTGGGTC CTTCCGACTATTTCAT	Cloning	*dot-1.3* homolgy arm adding to mNeonGreen-SEC vector (PDD268). Overlapping sequence to vector PDD268 is in lower case.
dot-1.3-5'-homology arm	REV	catcgatgctcctgaggctcccgatgctccTTTGTTGC GATTGATTGTAGTCAAGT	Cloning	*dot-1.3* homolgy arm adding to mNeonGreen-SEC vector (PDD268). Overlapping sequence to vector PDD268 is in lower case. Synonymous mutation in PAM is labbled in red.
dot-1.3-3'-homology arm	FWD	cgtgattacaaggatgacgatgacaagagaTAAGTTA GTTTTTTTTACTGTGTTCGATT	Cloning	*dot-1.3* homolgy arm adding to mNeonGreen-SEC vector (PDD268). Overlapping sequence to vector PDD268 is in lower case.
dot-1.3-3'-homology arm	REV	ggaaacagctatgaccatgttatcgatttcTTCGAAGT AGCGTAGTCAGGTTCA	Cloning	*dot-1.3* homolgy arm adding to mNeonGreen-SEC vector (PDD268). Overlapping sequence to vector PDD268 is in lower case.
