# Supplementary material for: Lysosomes Signal through the Epigenome to Regulate Longevity across Generations
Source: Science. Author manuscript; Available in PMC 2026 Jan 24. (PMC12831228; doi:10.1126/science.adn8754)
Supplement: Table S6_strains_20250204 [file NIHMS2127653-supplement-Table_S6_strains_20250204.pdf]

Table S6. List of *C. elegans* strains used in this study.

Strain/Usage	Genotype	Source	Lab Code
N2	*wild type*	CGC	
lipl-4 Tg	*raxIs3[ges-1p::lipl-4::sl2-GFP; myo-2p::mCherry]*		MCW14
H3.3(lf)	*his-69&his-70(uge44); his-72(tm2066); his-74(uge18); his-71(ok2289)*	CGC	FAS43
lipl-4 Tg;H3.3(lf)	*raxEx402[ges-1p::lipl-4::sl2-GFP]; his-69&his-70(uge44); his-72(tm2066); his-74(uge18); his-71(ok2289)*		MCW1150
his-71(lf)	*his-71(ok2289)*	CGC	RB1781
lipl-4 Tg;his-71(lf);	*raxIs3[ges-1p::lipl-4::sl2-GFP]; his-71(ok2289)*		MCW1271
his-69&70(lf)	*his-69&70(uge44)*		FAS65
lipl-4 Tg;his-69&70(lf)	*raxIs3[ges-1p::lipl-4::sl2-GFP]; his-69&70(uge44)*		MCW1270
intestine-specific auxin-induced HIS-71 degradation in WT background	*ieSi61 [ges-1p::TIR1::mRuby::unc-54 3'UTR + Cbr-unc-119(+)]; [his-71::mNeonGreen::AID]*		MCW1541
intestine-specific auxin-induced HIS-71 degradation in lipl-4 Tg background	*ieSi61 [ges-1p::TIR1::mRuby::unc-54 3'UTR + Cbr-unc-119(+)]; [his-71::mNeonGreen::AID]; raxIs3[ges-1p::lipl-4::sl2-GFP; myo-2p::mCherry]*		MCW1548
his-71::3xflag int-Tg	*raxIs191[ges-1p::his-71cds::3xflag::unc-54 3'UTR; myo-2p::mCherry]*		MCW1628
his-71 int-Tg	*raxEx541[ges-1p::his-71::sl2-GFP; myo-2p::mCherry]*		MCW1350
germline-specific auxin-induced HIS-71 degradation in WT background	*ieSi64 [gld-1p::TIR1::mRuby::gld-1 3'UTR + Cbr-unc-119(+)]; [his-71::mNeonGreen::AID]*		MCW1539
germline-specific auxin-induced HIS-71 degradation in lipl-4 Tg background	*ieSi64 [gld-1p::TIR1::mRuby::gld-1 3'UTR + Cbr-unc-119(+)]; [his-71::mNeonGreen::AID]; raxIs3[ges-1p::lipl-4::sl2-GFP; myo-2p::mCherry]*		MCW1546
his-71 germ-Tg	*raxEx535[pie-1p::his-71::sl2-GFP; myo-2p::mCherry]*		MCW1321
lipl-4 Tg;dot-1.3(lf)	*dot-1.3(ok2831); raxIs3[ges-1p::lipl-4::sl2-GFP; myo-2p::mCherry]*		MCW1072
lipl-4 Tg;dot-1.1(lf)	*dot-1.1(ok2154); raxIs3[ges-1p::lipl-4::sl2-GFP; myo-2p::mCherry]*		MCW1098
dot-1.3 endogenously tagged with mNeonGreen	*[dot-1.3::mNeonGreen::3xflag]*		MCW1339
RNAi defective control in lipl-4 Tg background	*rde-1(ne219); raxEx402[ges-1p::lipl-4::sl2-GFP]*		MCW1564
germline knockdown in WT background	*rde-1(ne219); jamSi2 [mex-5p::rde-1(+)]*	CGC	AMJ345
germline knockdown in lipl-4 Tg background	*rde-1(ne219); jamSi2 [mex-5p::rde-1(+)]; raxEx402[ges-1p::lipl-4::sl2-GFP]*		MCW1057
germline rescue of dot-1.3 expression in dot-1.3(lf);lipl-4 Tg background	*dot-1.3(ok2831); raxIs3[ges-1p::lipl-4::sl2-GFP]; Ex[pie-1p::dot-1.3::sl2-RFP::unc-54 3'UTR; myo-2p::GFP]*		MCW1155
intestine knockdown in WT background	*rde-1(ne219); Is[ges-1p::RDE-1::unc54 3'UTR; myo-2p::RFP3]*	gift from Justine Mello	JM45
intestine knockdown in lipl-4 Tg background	*rde-1(ne219); Is[ges-1p::RDE-1::unc54 3'UTR; myo-2p::RFP3]; raxEx402[ges-1p::lipl-4::sl2-GFP]*		MCW1056

intestine rescue of dot-1.3 expression in dot-1.3(lf);lipl-4 Tg background	dot-1.3 (ok2831); raxIs3[ges-1p::lipl-4::sl2-GFP]; Ex[ges-1p::dot-1.3::sl2-RFP::unc-54 3'UTR; myo-2::GFP]		MCW1228
dot-1.3 Tg	raxIs171[pie-1p::dot-1.3::sl2-RFP::unc-54 3UTR; myo-2p::GFP]		MCW1569
dot-1.3(lf)	dot-1.3(ok2831)	CGC	VC2294
dot-1.3(lf);his-71 germ-Tg	dot-1.3(ok2831); raxEx535[pie-1p::his-71::sl2-GFP; myo-2p::mCherry]		MCW1372
his-71K79A germ-Tg	raxEx613[pie-1p::HIS-71K79A::sl2-GFP; myo-2p::mCherry]		MCW1527
dot-1.3 Tg;H3.3(lf)	his-69&his-70(uge44); his-72(tm2066); his-74(uge18); his-71(ok2289); Ex[pie-1p::dot-1.3::sl2-RFP::unc-54 3UTR; myo-2p::GFP]		MCW1406
dot-1.3 Tg;his-71(lf)	his-71(ok2289); Ex[pie-1p::dot-1.3::sl2-RFP::unc-54 3UTR; myo-2p::GFP]		MCW1371
raga-1(lf)	raga-1(ok386)	CGC	VC222
raga-1(lf);his-71(lf)	raga-1(ok386); his-71(ok2289)		MCW1293
daf-2(lf)	daf-2(e1370)	CGC	CB1370
daf-2(lf);his-71(lf)	daf-2(e1370); his-71(ok2289)		MCW1300
glp-1(lf)	glp-1(e2141)	CGC	VC2294
glp-1(lf);his-71(lf)	glp-1(e2141); his-71(ok2289)		MCW1302
eat-2(lf)	eat-2(ad1116)	CGC	DA1116
eat-2(lf);his-71(lf)	eat-2(ad1116); his-71(ok2289)		MCW1301
raga-1(lf);dot-1.3(lf)	raga-1(ok386); dot-1.3(ok2831)		MCW1288
daf-2(lf);dot-1.3(lf)	daf-2(e1370); dot-1.3(ok2831)		MCW1178
glp-1(lf);dot-1.3(lf)	glp-1(e2141); dot-1.3(ok2831)		MCW1177
eat-2(lf);dot-1.3(lf)	eat-2(ad1116); dot-1.3(ok2831)		MCW1203
aak-2 Tg	uthIs248 [aak-2p::aak-2(genomic aa1-321)::GFP::unc-54 3'UTR + myo-2p::tdTOMATO]		WBM60
aak-2 lyso-Tg	raxEx632[ges-1p::lmp1::3xGAS::aak-2::HA; myo-2p::GFP]		MCW1653
wrmScarlet lyso-Tg	raxEx633[ges-1p::lmp1::3xGAS::wrmScarlet::HA; lin-44p::GFP]		MCW1654
aak-2 lyso-Tg	raxIs195[ges-1p::lmp1::3xGAS::aak-2::HA; myo-2p::GFP]		MCW1951
lipl-4(lf)	lipl-4(tm4417)		tm4417
