# Supplementary material for: Lysosomes Signal through the Epigenome to Regulate Longevity across Generations
Source: Science. Author manuscript; Available in PMC 2026 Jan 24. (PMC12831228; doi:10.1126/science.adn8754)
Supplement: adn8754_Supplementary_Final [file NIHMS2127653-supplement-adn8754_Supplementary_Final.pdf]

Science
AAAS

Supplementary Materials for

Lysosomes Signal through the Epigenome to Regulate Longevity across Generations

Qinghao Zhang, Weiwei Dang, Meng C. Wang*

Correspondence to: mengwang@janelia.hhmi.org

The PDF file includes:

Materials and Methods
Figs. S1 to S6
References (39-58)

Other Supplementary Materials for this manuscript include the following:

Tables S1 to S6 (Excel format)
Custom Code (with usage instructions)
Cell Radial Profiling_v1.1.zip (newly developed ImageJ plugin)
MDAR Reproducibility Checklist

Materials and Methods

C. elegans strains and maintenance

All *C. elegans* strains were maintained on standard nematode growth medium (NGM) agar plates seeded with a pre-cultured common laboratory bacterial strain *E. coli* OP50 (Straptomycin-resistant) at 20 °C unless otherwise stated. Full list of *C. elegans* strains used in this study is provided in the Table S6.

Generation of transgenic plasmid and transgenic worm strains

The Gibson assembly (NEBuilder® HiFi DNA Assembly) and restriction enzyme cloning were used to generate the tissue-specific expression plasmid vector of *his-71* and *dot-1.3* based on the backbone of pJM23 plasmid. The plasmid *ges-1p::his-71::sl2-GFP::unc-54 3'UTR* (pQZ1.7) contains a 2155 bp of *ges-1* promoter (*ges-1p*) region upstream of the full length *his-71* cDNA, followed by an *sl2-GFP::unc-54 3'UTR* sequence. The *his-71* cDNA fragment was generated by high-fidelity PCR amplification from wild-type N2 worm cDNA mixture which is reversely transcribed from total RNA. The sequence of *ges-1p* and *sl2-GFP::unc-54 3'UTR* were cloned from the plasmids used in our previous study (*8*). To express *his-71* in the germline, we digested the plasmid pQZ1.7 using restriction enzymes FseI/AscI and replaced the *ges-1p* with the *pie-1* promoter (*pie-1p*) to construct the plasmid pQZ1.11. The *pie-1p* sequence was cloned from pPK605 plasmid, a gift from Patricia Kuwabara (Addgene #38148). The HIS-71K79A mutation plasmid was generated by Q5® Site-Directed Mutagenesis Kit based on the plasmid pQZ1.11. To express the chimeric HIS-71 protein tagged with three consecutive FLAG sequences (3×FLAG) in the intestine, the plasmid *ges-1p::his-71::3×flag::unc-54 3'UTR* (pQZ6.0) was constructed based on the backbone of pQZ1.7 by Gibson assembly, which contains the *ges-1p*, the *his-71* coding region fused to a synthesized 3×FLAG fragment followed by an artificial stop codon (synthesized by GenScript USA Inc.), and the *unc-54* 3'UTR. To express *dot-1.3* in the germline, the plasmid *pie-1p::dot-1.3::sl2-RFP::unc-54 3'UTR* was generated, containing a *pie-1p* upstream of the *dot-1.3* coding sequence with its own stop codon followed by an *sl2-RFP::unc-54 3'UTR*. The *dot-1.3* coding sequence was cloned from wild-type N2 worm cDNA mixture by high-fidelity PCR amplification as mentioned above. To express the intestinal lysosome-tethered AAK-2, the plasmid pQZ5.1 was constructed using Gibson assembly based on pQZ6.0 backbone including the *ges-1p* and the *unc-54 3'UTR*. The synthesized coding region contains the *lmp-1* and the *aak-2* (isoform a) coding regions, terminated by an artificial stop codon (GenScript USA Inc.). To generate a control for pQZ5.1, *aak-2* coding region was replaced with the fluorescent maker *mScarlet* (GenScript USA Inc.), yielding plasmid pQZ5.2. All fragments generated via PCR amplification were verified by Sanger sequencing and the primers used in transgenic plasmid construction were listed in the Table S5.

Transgenic strains carrying extrachromosomal arrays were generated as previously described (*8*). Briefly, plasmid together with co-injection marker was injected into the gonad of young adult hermaphrodite worms at a final concentration of 10 ng/µL. The plasmid *myo-2p::mCherry* (pCFJ90, Addgene #19327), a gift from Erik Jorgensen, was used as the co-injection marker for

sl2-GFP strains or for *his-71::3×flag int-Tg* strain, and the plasmid *myo-2p::GFP* (pBCN27, Addgene #26347), a gift from Ben Lehner, was used for *sl2-RFP* strains and *aak-2 lyso-Tg* strain. The plasmid *lin-44p::GFP* was used as the co-injection marker for *mScarlet lyso-Tg* strain. The fluorescence from co-injection maker and/or the trans-spliced fluorescent reporters was employed to tell the worm candidates containing extra-chromosomal arrays and maintain the strain. The extrachromosomal arrays were further integrated into worm genome by gamma-ray irradiation as previously described (*6*). Integrated transgenic strains have been backcrossed at least six times before used in any experiments. All the transgenic strains generated in this study were listed in the Table S6.

CRISPR-Cas9 genome knock-in

The self-excising cassette (SEC) selection based CRISPR-Cas9 genome editing was exploited to knock in fluorescent protein tag as previously described (*39*). Using Cas9-triggered homologous recombination, we tagged the endogenous DOT-1.3 with mNeonGreen and the HIS-71 with mNeonGreen fused via a flexible linker to an auxin-inducible degron (AID) minimal sequence (*40*) at its carboxyl terminus. The sgRNA sequences were inserted into the Cas9-sgRNA vector (PDD162, Addgene #47549), a gift from Bob Goldstein. The sgRNAs targeting *dot-1.3* was 5'-gactacaatcaatcgcaaca-3'. The sgRNA targeting *his-71* was 5'-acgtttatgcacgttctcca-3'. Homology arms for either *dot-1.3* or *his-71* gene were generated by high-fidelity PCR amplification from wild-type N2 worm genomic DNA with synonymous mutation at PAM motif. By using Gibson assembly, the homology arms of either *dot-1.3* or *his-71* gene were inserted into the donor vector pDD268 which had been digested by restriction enzymes AvrII and SpeI. To insert the degron label, the 3×FLAG sequence in the recombinant vector pDD268 with *his-71* homology arms was replaced with the AID minimal sequence using Gibson assembly. The young hermaphrodites at day-1 adulthood were injected with the plasmid DNA mixture including Cas9-sgRNA (50 ng/μL), donor vector containing homology arms (10 ng/μL) and co-injection markers. After hygromycin-resistant selection, the homozygous candidates containing correct insertions were subjected to heat shock treatment to remove the selectable marker dependent on the SEC-excising. All tagged strains were outcrossed to wild-type N2 worms for at least six times before any experimental usage. The tag-in homozygotes were preliminarily confirmed by PCR amplification and were finally confirmed by high-fidelity PCR amplification followed by Sanger sequencing.

Western blotting

Age-synchronized worms were harvested to prepare worm lysates for SDS-PAGE and Western blot analysis. Worms growing on the standard NGM plates seeded with *E. coli* OP50 were collected on the first day of adulthood. Worms subjected to HT115 RNAi knockdown treatments were grown until day six of adulthood on plates supplemented with 5'-fluorodeoxyuridine (FUdR) at a final concentration of 25 μM from the fourth larval (L4) stage. Worms were collected by M9 washing and remaining eggs were excluded by further M9 washing on appropriate strainer for three times. Worm pellets were snap-frozen in liquid nitrogen and stored

at -80 °C. Frozen worm samples were ground using motorized pestle on ice and resuspended in lysis buffer (50 mM Tris·HCl (pH 8), 300 mM NaCl, 1 mM EDTA, 0.5% Triton X-100, 1mM PMSF, 1× cOmplete protease inhibitor cocktail) followed by sonication. Homogenates were centrifuged at 13000 g and supernatant was aliquoted and frozen at -80 °C. Total protein concentration was quantified by BCA protein assay. Worm lysates were denatured at 70 °C in LDS sample buffer for 10 min before electrophoresis on the NuPAGE Bis-Tris gels. Standard Western blot analysis was conducted on PVDF membrane after blocked with Everyblot blocking buffer (Bio-Rad) or 5% BSA. Blots were incubated with the following primary antibodies at 4 °C overnight: anti-H3K79me1 (CST #12522) at 1: 2000, anti-H3K79me2 (CST #5427) at 1:2000, anti-H3K79me3 (CST #4260) at 1:2000, anti-H3 (CST #4499) at 1:2000, anti-β-actin (Santa Cruz #sc-47778) at 1:5000 and anti-α-tubulin (Abcam #ab52866) at 1:5000. Secondary antibodies were anti-rabbit IgG-HRP (Santa Cruz #sc-2357) at 1:5000 or anti-mouse m-IgG Fc BP-HRP (Santa Cruz #sc-525409) at 1:5000. Protein detection was performed using chemiluminescent substrate (Pierce™ ECL Plus Western Blotting Substrate) and images acquired by using a gel imaging system (ImageQuant LAS 500, Thermo Fisher Scientific) or the autoradiography film (HyBlot CL).

In Western blot images, numbers above the lane represent the relative H3K79 methylation levels which were first normalized by the H3 intensity in the same column and then by the mean relative value of the first two WT replicates. The bar charts summarize all the Western blot replicates. Statistical analyses were performed by unpaired t-test with Welch's correction or ordinary one-way ANOVA followed by Tukey test for multiple comparison analyses, as indicated.

<u>RNA sequencing and analysis</u>

Total RNA from the *lipl-4 Tg* and WT worms was isolated from 3000 worms of synchronized age on the first day of adulthood and remnant embryos were excluded by washing on the strainers. Total RNA isolation was performed by using Trizol extraction combined with Qiagen column purification. Sequencing libraries were prepared using the Truseq Stranded mRNA sample preparation kit (Illumina) according to the manufacturer's instructions. Libraries were pooled together and sequenced using Illumina NextSeq 500 system. Sequencing reads were aligned to the *C. elegans* reference genome and counted using STAR 2.7.9a aligner, followed by importing to DEseq2 for differential gene expression analysis (*41*, *42*). The statistical significance was defined by $|\text{fold change}| \geq 1.5$; p value <0.05, FDR < 0.05.

<u>ChIP sequencing and analysis</u>

Age-synchronized day-1 young adult worms (~20,000) were harvested and washed three times with M9 buffer on strainers to avoid remnant eggs. After further wash in phosphate-buffered saline (PBS) for once, worm pellets were snap-frozen in liquid nitrogen, followed by one round of freeze-thaw cycle and store at -80 °C before crosslinking. Cuticles of the frozen worms were cracked open by gently ground on ice using pestle. Worm samples were fixed in the freshly

prepared 1% formaldehyde/PBS solution for 10 min while stroked for ten times and mixed upside down in on a tube rotator. Crosslinking was quenched by adding glycine solution to a final concentration of 125 mM and mixed upside down for another 5 min. After centrifugation at 4 °C, worm samples were washed three times with ice-cold PBS buffer supplemented with 1× proteinase-inhibitor cOmplete cocktail (Roche) and 100 μM PMSF. Next, worm pellets were resuspended in ice-cold FA buffer (50 mM HEPES/KOH pH 7.5, 150 mM NaCl, 1mM EDTA, 1% Triton X-100, 0.1% sodium deoxycholate) supplemented with 1× proteinase-inhibitor cOmplete cocktail and 500 μM PMSF, and sonicated at 4 °C using a Branson 250 sonicator (25% duty cycle, power output at 4) for 16 min to shear chromatin. The sonicated samples were centrifuged at 16000 g for 15 min at 4 °C, and the supernatant was collected as the chromatin extract, with its concentration quantified using the BCA protein assay. Chromatin extract was diluted into 0.2 μg/μL with 1% Sarkosyl/ FA buffer supplemented with 1× proteinase-inhibitor cOmplete cocktail and 1 mM PMSF, before overnight immunoprecipitation (IP) at 4 °C on a rotator. The ChIP-seq formulated antibodies were anti-histone H3 (CST #4620), H3K79me2 (CST #5427), H3K79me3 (Abcam #ab2621), all in a final concentration of 4 ng/μL. 30 μL ChIP-grade Protein G Magnetic beads (CST #9006) were added to each 500 μL IP reaction and incubate for 2 hours at 4 °C with rotation. Beads were separated on a magnetic rack and subjected to 10 min washes with following ice-cold buffers in turn: FA buffer, FA-500 buffer (50 mM HEPES/KOH pH 7.5, 500 mM NaCl, 1mM EDTA, 1% Triton X-100, 0.1% sodium deoxycholate), FA-1000 buffer (50 mM HEPES/KOH pH 7.5, 1 M NaCl, 1mM EDTA, 1% Triton X-100, 0.1% sodium deoxycholate), TEL buffer (0.25 M LiCl, 1% NP-40, 1% sodium deoxycholate, 1 mM EDTA, 10 mM Tris-HCl, pH 8.0) and TE buffer (10 mM Tris-HCl pH 8.0, 1 mM EDTA). Elusion was performed at 65 °C for 30 min in 150 μL elution buffer per sample (1% SDS in TE buffer with 250 mM NaCl). After overnight reverse crosslinking at 65 °C in 1% SDS TE buffer with 270 mM NaCl, the chromatin solutions were treated with 10 μg/μL RNase at 37 °C and subsequently 10 mg/mL proteinase K at 65 °C for one hour. The liberated DNA was purified using QIAquick PCR purification kit (Qiagen) and then quantified by Qubit® dsDNA HS Assay Kits.

Sequencing libraries were constructed by NEBNext® Ultra™ II DNA Library Prep with Sample Purification Beads kit (NEB #E7103) according to manufacturer's instructions. Libraries were pooled together and sequenced on the NextSeq 500 system (Illumina) or the NextSeq 2000 system (Illumina).

Raw reads of ChIP-seq were filtered using Trim Galore (version 0.4.5) (*43*) and aligned to the *C. elegans* reference genome by Bowtie2 (version 2.1.0) (*44*). The Samtools (version 1.7) (*45*) was used to converse sequence alignment/map (sam) format to binary alignment/map (bam). The MACS2 algorithm (version 2.1.0) (*46*) was used to call peaks for H3K79me2 or H3K79me3, with histone H3 ChIP-seq reads serving as internal controls for normalization between samples and to offsetting differences from nucleosome input.

To visualize ChIP-seq peaks, data from replicates were merged into a single track by subcommand macs2 bdgcmp to generate the fold enrichment of the normalized pileup reads in bedGraph (bdg) format. After UCSC-bedClip cleanup, UCSC-BedGraphToBigWig package was used to convert bdg file to bigwig (bw) file (*47*), and the later was visualized by the Integrative Genomics Viewer (IGV) software (*48*).

The Deeptools2 (*48*) was used to create the metaprofile by its subcommand computeMatrix and plotProfile based on the single-track ChIP-seq data as aforementioned, where a window of ±1kb genomic region flanking the TSS was specified. The R package DiffBind (*50*, *51*) was used to

analyze the differential occupancy of H3K79me2/3 marks in *lipl-4 Tg* vs. WT worms (p value < 0.01, FDR < 0.05), followed by annotation using ChIPseeker (*52*, *53*). The R package EnhancedVolcano (version 1.16.0) (*54*) was used to generate the volcano plots and package Venneuler (1.1-3) (*55*) was used to create Venn diagrams. The heatmap representing ChIP-seq&RNA-seq combinational analysis was generated using ComplexHeatmap package (*56*), where the normalized mean peak read concentrations obtained by DiffBind and the normalized count values obtained by DESseq2 from each replicate were illustrated.

RNA interference (RNAi) experiments

RNAi clones used in this study were sourced from libraries generated in the laboratories of Dr. Julie Ahringer (*dot-1.1*, *dot-1.2*, *dot-1.3*, *D1053.2, cco-1*), Dr. Marc Vidal (*skn-1*, *rme-2*, *dot-1.4*, *dot-1.5, raga-1*), or constructed in-house (*vits*). For the *vits* RNAi construct, a synthetic mosaic DNA sequence (GenScript USA Inc.) was designed, containing coding regions of vitellogenin proteins: 333 bp from homologous sequence between *vit-1* and *vit-2*, 333 bp between *vit-3*, *vit-4* and *vit-5*, and 333 bp from *vit-6*. This sequence was cloned into the L4440 vector using XbaI/HindIII restriction sites. All RNAi clones were verified by Sanger sequencing. RNAi-based experiments were conducted using *E. coli* HT115 bacteria, with L4440 empty vector bacteria used as controls.

Auxin-induced degradation treatment

Egg-laying was performed within 6-8 hours on 1 mM (*40*) auxin-containing NGM plates seeded with *E. coli* OP50 bacteria lawns. Auxin-induced degradation was applied to worms carrying endogenous degron-tagged chimeric HIS-71 protein throughout the entire larval stages and up to mid-day-1 adulthood, marked by the onset of egg-laying. Worms were then picked and transferred to standard NGM plates with OP50 bacteria lawns for reproduction and descendant collection.

L1 larvae starvation

Starvation treatment was conducted as previously described (*31*). Worm eggs were isolated using a bleach-based protocol and split between NGM plates seeded with OP50 bacteria (fed condition) and empty NGM plates (starvation condition). Larvae hatched on empty NGM plates were arrested at the L1 stage and starved for six days. After starvation treatment, L1 larvae were collected using M9 buffer and transferred to NGM plates seeded with OP50 bacteria to resume growth.

Lifespan assays

Lifespan assays and genetic crosses were all performed at 20 °C without 5-fluoro-2'-deoxyuridine (FUdR) as previously described with minor modification (*8*). Regarding the lifespan assays on standard NGM plates seeded with OP50 bacteria, worms were age-synchronized by egg-laying. As for RNAi knockdown condition, worms were age-synchronized by bleach-based egg isolation followed by starvation in M9 buffer for 16-18 hours before placing onto appropriate RNAi plates. In all conditions, worms were age-synchronized again by manual

picking at mid-L4 stage and designated as Day 0. In lifespan assays, adult worms were transferred to new plates every two days, coinciding with survival assessment for Kaplan-Meier survival estimation. All mutants or integrated transgenic strains were backcrossed at least six times before lifespan assays. As for the transgenic strains carrying extrachromosomal array obtained by micro-injection, worms were backcrossed at least three times before the lifespan assays. During the genetic crosses, the mutation was genotyped as in single worm by PCR (deletion mutation) or DNA sequencing (point mutation) to isolate heterozygotes and homozygotes. Additionally, *raga-1(lf)* homozygotes were maintained at 15 °C following the backcross to preserve their longevity phenotype. Primers used in this study are listed in Table S5. The information about biological replicates, samples size and mean lifespan values are summarized in the Tables S1, S2 and S4.

Quantitative RT-PCR (qRT-PCR)

To isolate total RNA from whole worms, worms at day-1 adulthood under each condition were harvested and washed by M9 buffer for three times on strainers. To isolate total RNA from specific tissues, at least 40 worms at day-1 adulthood for each replicate were picked and washed by M9 buffer. Under the stereo microscope, the germline and intestine from the same group of worms were released by incising the worm head using needles (27G1/2 or 30G1/2, Becton Dickinson) in ice-cold M9 buffer. The anterior intestine and full germline arms containing mature oocytes were collected respectively.
Total RNA from whole worms or specific tissue samples were isolated using Trizol (Invitrogen) and Qiagen column with on-column DNA digestion/purification. The cDNA synthesis was performed on the same amount of total RNA between controls and treatments by using the amfiRivert Platinum cDNA Synthesis Master Mix (GenDEPOT) for whole worm samples or the Superscript™ IV VILO™ Master Mix kit (Invitrogen) for tissue samples.
The qPCR was performed using Power SYBR™ Green PCR Master Mix (Applied Biosystems) in a QuantStudio™ 3 Real-Time PCR machine (Applied Biosystems). All values were normalized to *rpl-32* as an internal control as previously described (*8*). Primers used in this study are listed in Table S5.

Hoechst staining *in vivo*

100 µL of 1 mg/mL Hoechst 33342 (Invitrogen) was added on the top of *E. coli* OP50 bacteria lawn (100 µL) on NGM plates, and the Hoechst plates were maintained in dark at room temperature overnight before use. Egg-laying was performed on Hoechst plates using worms carrying endogenous fluorescent tag, DOT-1.3::mNeonGreen or HIS-71::mNeonGreen::AID. To prepare day-1 adult worms, L4 larvae were picked and transferred to a fresh Hoechst plate on the day before imaging. For imaging, the L4 larvae of DOT-1.3::mNeonGreen worms or the day-1 adult of HIS-71::mNeonGreen::AID worms were mounted on 2% agarose pad containing 2% sodium azide as anesthetic on glass microscope slides. Fluorescent images were taken using confocal FV3000 (Olympus) equipped with 60× objective.

Immunofluorescence staining and fluorescence intensity profiling

Age-synchronized worms were grown on RNAi plates, followed by a secondary synchronization at mid-L4 stage with manual picking, and then maintained on the same RNAi condition until day-2 adulthood. To release the germline, the worm head was incised by needles in ice-cold M9 buffer under the stereo microscope. The worm germlines were fixed in 4% paraformaldehyde for 10 mins, followed by twice washes using PBST (PBS with 0.1% Tween-20). Dissected germlines were precipitated at 1000 g for 20 s and treated with ice-cold methanol for an hour at -20 °C. After three washes with PBST at room temperature, germline samples were blocked with 5% BSA for 30 mins. Subsequently, the primary antibody ANTI-FLAG® M2 monoclonal antibody (Sigma #F3165) was applied at 1:200 in 5% BSA, and samples were incubated at 4 °C overnight. After three washes with PBST, the secondary antibody Alexa Fluor™ 633 goat anti-mouse IgG1 (Invitrogen #A-21126) was applied at 1:500 in 5% BSA for two hours, followed by three washes in PBST. The samples were mounted with VECTASHIELD® anti-fade mounting medium with DAPI (Vector Laboratories). Imaging was performed using a confocal FV3000 microscope (Olympus) with 60× objective.

In the image analyses, the oocytes at the proximal -2 position were delineated as the region of interest (ROI), where we quantitatively assessed the distribution of HIS-71::3×FLAG fluorescence intensities, extending from the oocyte nucleus to the periphery. This assessment calls the FUJI/ImageJ Radial Profile Extended plugin (https://imagej.net/ij/plugins/radial-profile-ext.html or questpharma.u-strasbg.fr/html/radial-profile-ext.html), combined with customized scripts, with an integration angle of 45 degrees and starting angles of -135, -45, +45, and +135 degrees, which divided the ROI into four quadrants for pixel-wise intensity integration. For each biological replicate, consisting of 7-15 oocytes under a given condition, we averaged all the intensity values at identical pixel positions along the radius, which represented both intensity levels and their spatial distribution across the radius. Four replicates were used for each condition, with a total of 37-50 oocytes per condition. The intensity values, from every five-pixel interval along the radius within 0-100 pixels (distance to nuclear fluorescence center), were further averaged to generate the radial plots. Subsequent statistical analyses were performed using two-way ANOVA (distance to nuclear fluorescence center × condition), followed by Bonferroni's multiple comparisons test, to compare (1) empty vector (EV) vs. negative control (Neg ctrl) conditions, (2) target gene RNAi knockdown (*rme-2* or *vits*) vs. Neg ctrl conditions, and (3) target gene (*rme-2* or *vits*) RNAi knockdown vs. EV conditions. The complete code is available in the Supplementary Custom Code section.

Statistical information

For survival analyses, the Kaplan-Meier method combined with the log-rank (Mantel-Cox) test was used for statistical analysis in SPSS software (IBM) or the online application OASIS2 (*57*). Subsequently, Fisher's method was employed to integrate the *p* values from biologically independent lifespan assay replicates, facilitated by the metap software (version 1.8) (*58*). The detailed information is listed in the Tables S1, S2 and S4. For results of qRT-PCR, Western blots and fluorescence intensity profiling, statistical analyses were performed on three or more biologically independent replicates by using GraphPad Prism 9 (GraphPad Software). For ChIP-

seq data analysis, two biologically independent replicates were used for each experimental condition. For RNA-seq data analysis, three biologically independent replicates were included, as previously described (*7*). In all cases, p values > 0.05 were considered not significant (n.s.), and asterisks indicate statistical significance as follows: *$p < 0.05$, **$p < 0.01$, ***$p < 0.001$, ****$p < 0.0001$. The statistical information for the experiments can also be found in Tables S1, S2 and S4, the figure legends, and the Materials and Methods section.

Fig. S1.

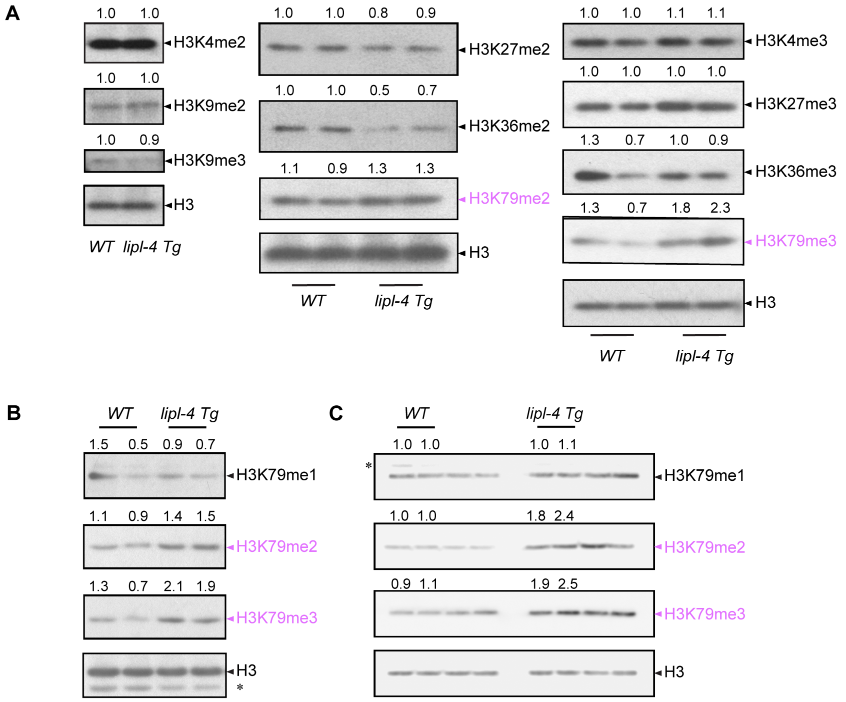
A
1.0 1.0
H3K4me2
1.0 1.0
H3K9me2
1.0 0.9
H3K9me3
H3
WT lipl-4 Tg
1.0 1.0 0.8 0.9
H3K27me2
1.0 1.0 0.5 0.7
H3K36me2
1.1 0.9 1.3 1.3
H3K79me2
H3
WT lipl-4 Tg
1.0 1.0 1.1 1.1
H3K4me3
1.0 1.0 1.0 1.0
H3K27me3
1.3 0.7 1.0 0.9
H3K36me3
1.3 0.7 1.8 2.3
H3K79me3
H3
WT lipl-4 Tg
B
WT lipl-4 Tg
1.5 0.5 0.9 0.7
H3K79me1
1.1 0.9 1.4 1.5
H3K79me2
1.3 0.7 2.1 1.9
H3K79me3
H3
*
C
WT lipl-4 Tg
1.0 1.0 1.0 1.1
*
H3K79me1
1.0 1.0 1.8 2.4
H3K79me2
0.9 1.1 1.9 2.5
H3K79me3
H3

fig. S1. Western blot images illustrating histone H3 PTM levels in *lipl-4 Tg* vs. WT worms. (**A**) Western blot screens of histone H3 PTM levels. (**B**, **C**) Western blot replicates on *lipl-4 Tg* worms. The asterisks indicate non-specific bands.

Fig. S2.

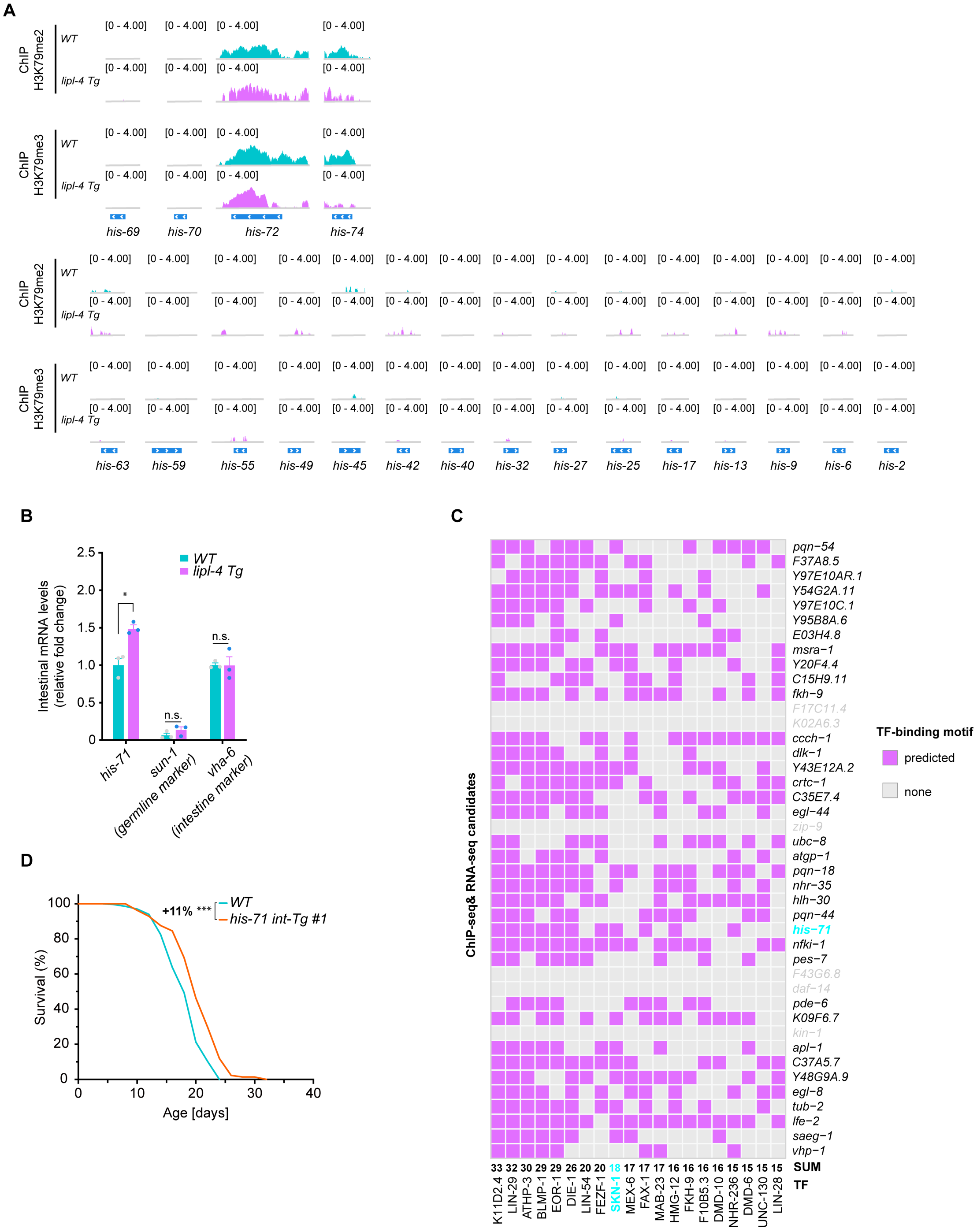
A
ChIP H3K79me2
ChIP H3K79me3
WT
lipl-4 Tg
[0 - 4.00]
his-69
his-70
his-72
his-74
his-63
his-59
his-55
his-49
his-45
his-42
his-40
his-32
his-27
his-25
his-17
his-13
his-9
his-6
his-2
B
Intestinal mRNA levels (relative fold change)
WT
lipl-4 Tg
*
n.s.
n.s.
his-71
sun-1 (germline marker)
vha-6 (intestine marker)
C
ChIP-seq& RNA-seq candidates
pqn−54
F37A8.5
Y97E10AR.1
Y54G2A.11
Y97E10C.1
Y95B8A.6
E03H4.8
msra−1
Y20F4.4
C15H9.11
fkh−9
F17C11.4
K02A6.3
ccch−1
dlk−1
Y43E12A.2
crtc−1
C35E7.4
egl−44
zip−9
ubc−8
atgp−1
pqn−18
nhr−35
hlh−30
pqn−44
his−71
nfki−1
pes−7
F43G6.8
daf−14
pde−6
K09F6.7
kin−1
apl−1
C37A5.7
Y48G9A.9
egl−8
tub−2
lfe−2
saeg−1
vhp−1
33 32 30 29 29 26 20 20 18 17 17 17 16 16 16 16 15 15 15 15
SUM
K11D2.4 LIN-29 ATHP-3 BLMP-1 EOR-1 DIE-1 LIN-54 FEZF-1 SKN-1 MEX-6 FAX-1 MAB-23 HMG-12 FKH-9 F10B5.3 DMD-10 NHR-236 DMD-6 UNC-130 LIN-28
TF
TF-binding motif
predicted
none
D
Survival (%)
Age [days]
+11% ***
WT
his-71 int-Tg #1

fig. S2. H3K79me2 and H3K79me3 deposition on H3.3 and H3, transcription factor analysis, and additional controls. (**A**) Distribution of H3K79me2 and H3K79me3 marks at genes encoding H3.3 variants (*his-69*, *his-70*, *his-72*, *his-74*) and canonical H3 (*his-2, his-6, his-9, his-13, his-17, his-25, his-27, his-32, his-40, his-42, his-45, his-49, his-55, his-59, his-63*) in WT and *lipl-4 Tg* worms. The y-axis of ChIP-seq track is shown in a log scale (0-4.00). (**B**) Intestinal *his-71* transcription is upregulated in *lipl-4 Tg* vs. WT worms. Tissue-specific gene markers were included for validation: *sun-1* for the germline and *vha-6* for the intestine. Error bars represent mean ± s.e.m., n.s., $p > 0.05$, *$p < 0.05$ (unpaired t-test, Welch's correction). (**C**) Heatmap showing the predicted transcription factor (TF)-binding motifs (name at the bottom) within the promoter of candidate genes (name on the right) that are both enriched with H3K79me2 and H3K79me3 marks and transcriptionally upregulated (> 1.5-fold) in *lipl-4 Tg* vs. WT worms. Violet indicates the presence of a predicted TF-binding motif within the promoter, while gray signifies its absence. The "SUM" row presents the total number of candidate genes with the assigned TF-binding motif. SKN-1 and *his-71* are highlighted in red, and the genes with promoters shorter than 1 kb are colored in gray. The *Caenorhabditis elegans* TF motifs with experimentally verified preferred binding sequences were sourced from the CisBP database v2.00. The software tool FIMO was utilized to scan and identify motif positions within 1000 bp promoter regions, based on a statistical cutoff of $p < 1.0e-4$. (**D**) Intestine-specific overexpression of *his-71* (*his-71 int-Tg*) extends lifespan. With n = 90 per replicate, 3 biological replicates; log-rank test followed by Fisher's method: ***$p < 0.001$. Percentage of lifespan extension (lower vs. upper) is labeled. Summary of lifespan replicates shown in table S2.

Fig. S3.

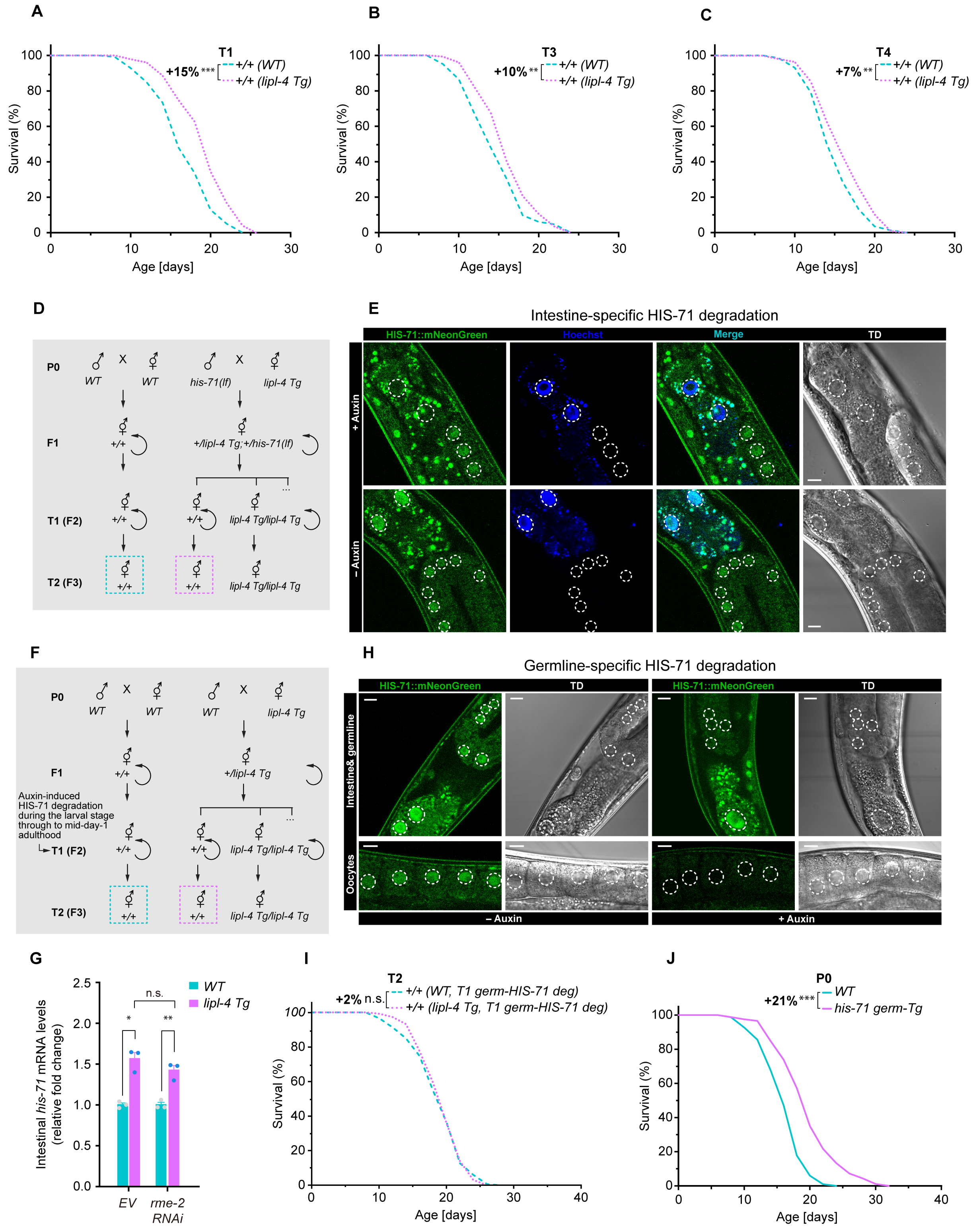
A
T1
+15% ***
+/+ (WT)
+/+ (lipl-4 Tg)
Survival (%)
Age [days]
B
T3
+10% **
+/+ (WT)
+/+ (lipl-4 Tg)
C
T4
+7% **
+/+ (WT)
+/+ (lipl-4 Tg)
D
P0
WT
his-71(lf)
lipl-4 Tg
F1
+/+
+/lipl-4 Tg;+/his-71(lf)
T1 (F2)
lipl-4 Tg/lipl-4 Tg
T2 (F3)
E
Intestine-specific HIS-71 degradation
HIS-71::mNeonGreen
Hoechst
Merge
TD
+ Auxin
– Auxin
F
+/lipl-4 Tg
Auxin-induced HIS-71 degradation during the larval stage through to mid-day-1 adulthood
H
Germline-specific HIS-71 degradation
Intestine& germline
Oocytes
– Auxin
+ Auxin
G
Intestinal his-71 mRNA levels (relative fold change)
WT
lipl-4 Tg
n.s.
EV
rme-2 RNAi
I
T2
+2% n.s.
+/+ (WT, T1 germ-HIS-71 deg)
+/+ (lipl-4 Tg, T1 germ-HIS-71 deg)
J
P0
+21% ***
WT
his-71 germ-Tg

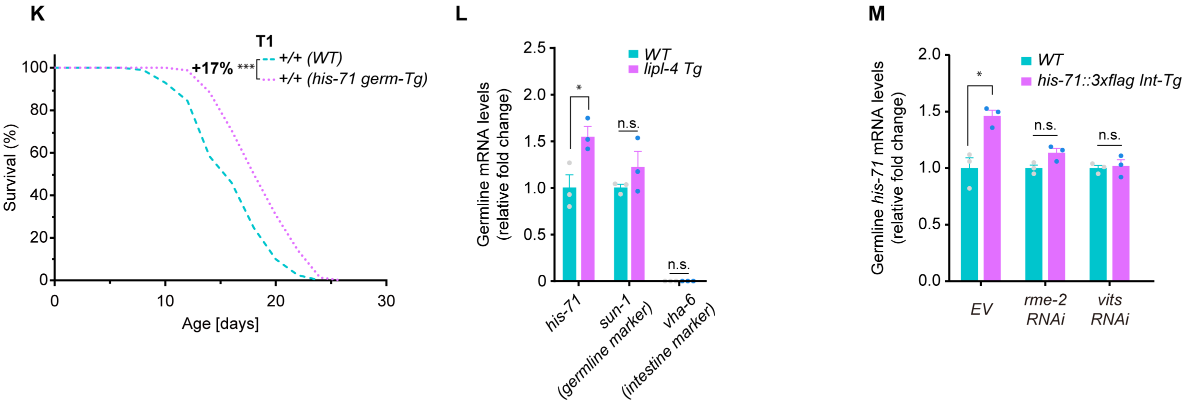
K
T1
Survival (%)
100
80
60
40
20
0
0
10
20
30
Age [days]
+17% ***
+/+ (WT)
+/+ (his-71 germ-Tg)
L
Germline mRNA levels
(relative fold change)
2.5
2.0
1.5
1.0
0.5
0
WT
lipl-4 Tg
*
n.s.
n.s.
his-71
sun-1
(germline marker)
vha-6
(intestine marker)
M
Germline his-71 mRNA levels
(relative fold change)
2.0
1.5
1.0
0.5
0.0
WT
his-71::3xflag Int-Tg
*
n.s.
n.s.
EV
rme-2
RNAi
vits
RNAi

fig. S3. Transgenerational longevity across generations, tissue-specific degradation validation, and *his-71* transcriptional coordination between tissues. (**A-C**) Lifespans of WT descendants originating from *lipl-4 Tg* and WT worms across generations. (**D**) Experimental design for genetic crosses and tracking of subsequent generations. T2 WT worms (framed with dotted boxes) were used for lifespan analyses. (**E**, **H**) Confocal images showing auxin-induced degradation of HIS-71 specifically in the intestine (E) and germline (H). Green, endogenous HIS-71 tagged with mNeonGreen; blue, nuclei stained with Hoechst. Merged images show fluorescence overlay. Transmission Detection (TD) imaging provides morphological context. Auxin-treated (+auxin) or non-auxin-treated (-auxin) are marked. Dotted lines encircle nuclei. Scale bar = 10 μm. In (H), top: intestine and germline; bottom: oocytes in focus. (**F**) Scheme for genetic crosses, generation tracking, and tissue-specific HIS-71 degradation. Lifespan analysis on T2 WT descendants (framed with dotted boxes). (**G**) RNAi knockdown of *rme-2* does not suppress the transcriptional upregulation of *his-71* in the intestine caused by *lipl-4 Tg*. (**I**) Germline degradation of HIS-71 in the T1 generation (T1 germ-HIS-71 deg) abolishes the transgenerational longevity in T2 WT from *lipl-4 Tg*. (**J**, **K**) Germline overexpression of *his-71* (*his-71 germ-Tg*) promoted longevity in the P0 generation and the T1 WT progeny. (**L**) Germline *his-71* transcription is upregulated in *lipl-4 Tg* vs. WT worms. (**M**) Intestinal overexpression of *his-71::3×flag* upregulates endogenous *his-71* transcription in the germline, which is suppressed by RNAi inactivation of *rme-2* or vitellogenin genes (*vits*). In (A-C, I-K), n = 90 per replicate, 3 biological replicates; log-rank test followed by Fisher's method: n.s., $p > 0.05$, $^{**}p < 0.01$, $^{***}p < 0.001$. Percentage of lifespan extension (lower vs. upper) is labeled. Summary of lifespan replicates shown in tables S1 and S2. In (G, L, M), error bars represent mean ± s.e.m., n.s., $p > 0.05$, $^{*}p < 0.05$, $^{**}p < 0.01$ (unpaired t-test, Welch's correction).

Fig. S4.

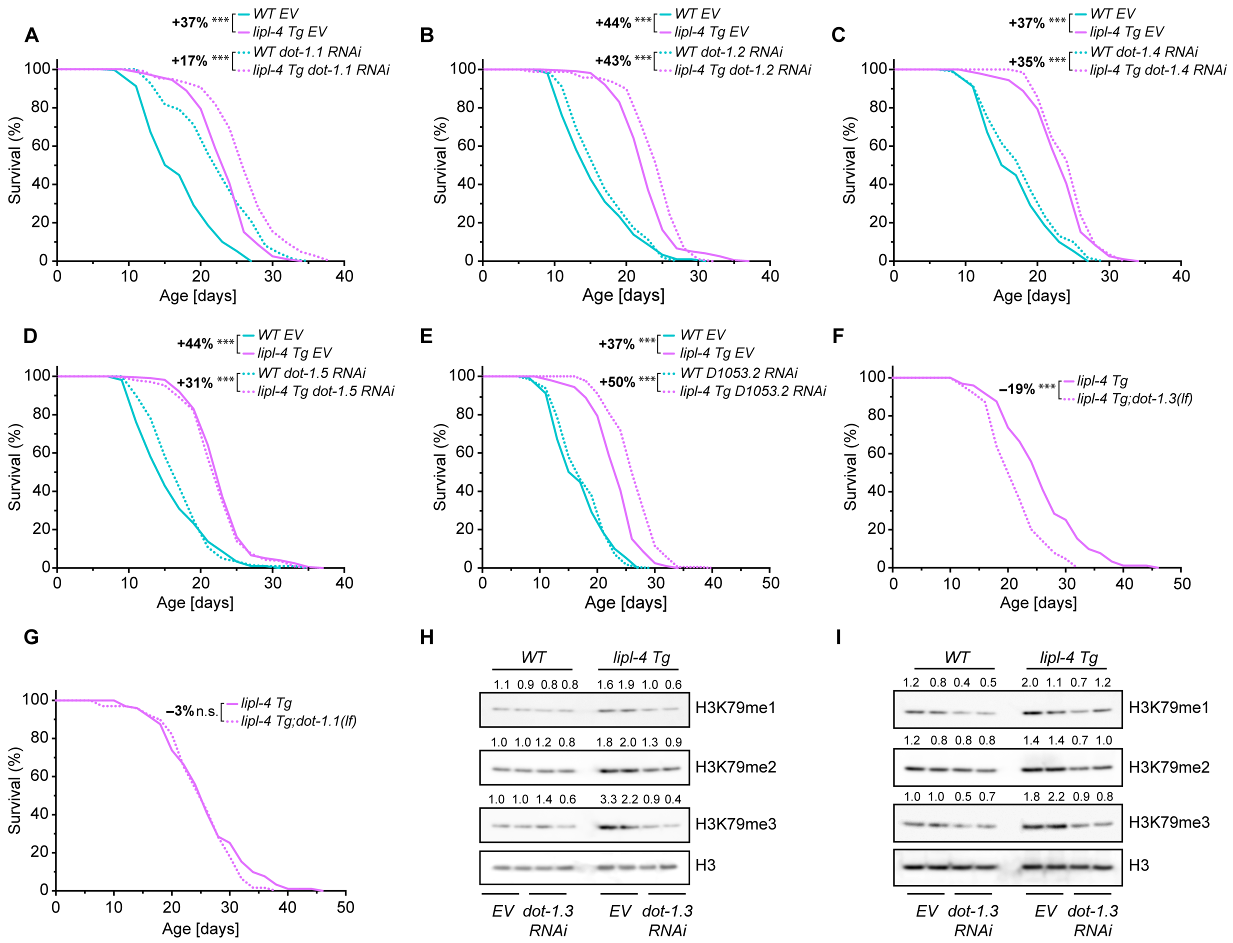
A
+37% *** WT EV
lipl-4 Tg EV
+17% *** WT dot-1.1 RNAi
lipl-4 Tg dot-1.1 RNAi
B
+44% *** WT EV
lipl-4 Tg EV
+43% *** WT dot-1.2 RNAi
lipl-4 Tg dot-1.2 RNAi
C
+37% *** WT EV
lipl-4 Tg EV
+35% *** WT dot-1.4 RNAi
lipl-4 Tg dot-1.4 RNAi
D
+44% *** WT EV
lipl-4 Tg EV
+31% *** WT dot-1.5 RNAi
lipl-4 Tg dot-1.5 RNAi
E
+37% *** WT EV
lipl-4 Tg EV
+50% *** WT D1053.2 RNAi
lipl-4 Tg D1053.2 RNAi
F
−19% *** lipl-4 Tg
lipl-4 Tg;dot-1.3(lf)
G
−3% n.s. lipl-4 Tg
lipl-4 Tg;dot-1.1(lf)
Survival (%)
Age [days]
H
WT
lipl-4 Tg
1.1 0.9 0.8 0.8 1.6 1.9 1.0 0.6
H3K79me1
1.0 1.0 1.2 0.8 1.8 2.0 1.3 0.9
H3K79me2
1.0 1.0 1.4 0.6 3.3 2.2 0.9 0.4
H3K79me3
H3
EV dot-1.3 RNAi EV dot-1.3 RNAi
I
WT
lipl-4 Tg
1.2 0.8 0.4 0.5 2.0 1.1 0.7 1.2
H3K79me1
1.2 0.8 0.8 0.8 1.4 1.4 0.7 1.0
H3K79me2
1.0 1.0 0.5 0.7 1.8 2.2 0.9 0.8
H3K79me3
H3
EV dot-1.3 RNAi EV dot-1.3 RNAi

fig. S4. H3K79 methyltransferase screens and the effect of *dot-1.3* on H3K79 methylation levels. (**A**-**E**) Effects of RNAi inactivation of H3K79 methyltransferase-encoding genes: *dot-1.1* (A), *dot-1.2* (B), *dot-1.4* (C), *dot-1.5* (D), *D1053.2* (E), on lifespans of WT and *lipl-4 Tg* worms. (**F**, **G**) *dot-1.3(lf)*, but not *dot-1.1(lf)*, mutant decreases lifespan of *lipl-4 Tg* worms. (**H**, **I**) Western blot replicates on worms with *dot-1.3* RNAi knockdown. In Kaplan-Meier survival curves (A-G), n = 60-90 worms per replicate, 3 biological replicates; log-rank test followed by Fisher's method: n.s., $p > 0.05$, ***$p < 0.001$. Percentage of lifespan extension (lower vs. upper, or indicated by arrows) is labeled. Summary of lifespan replicates shown in tables S2 and S4.

Fig. S5.

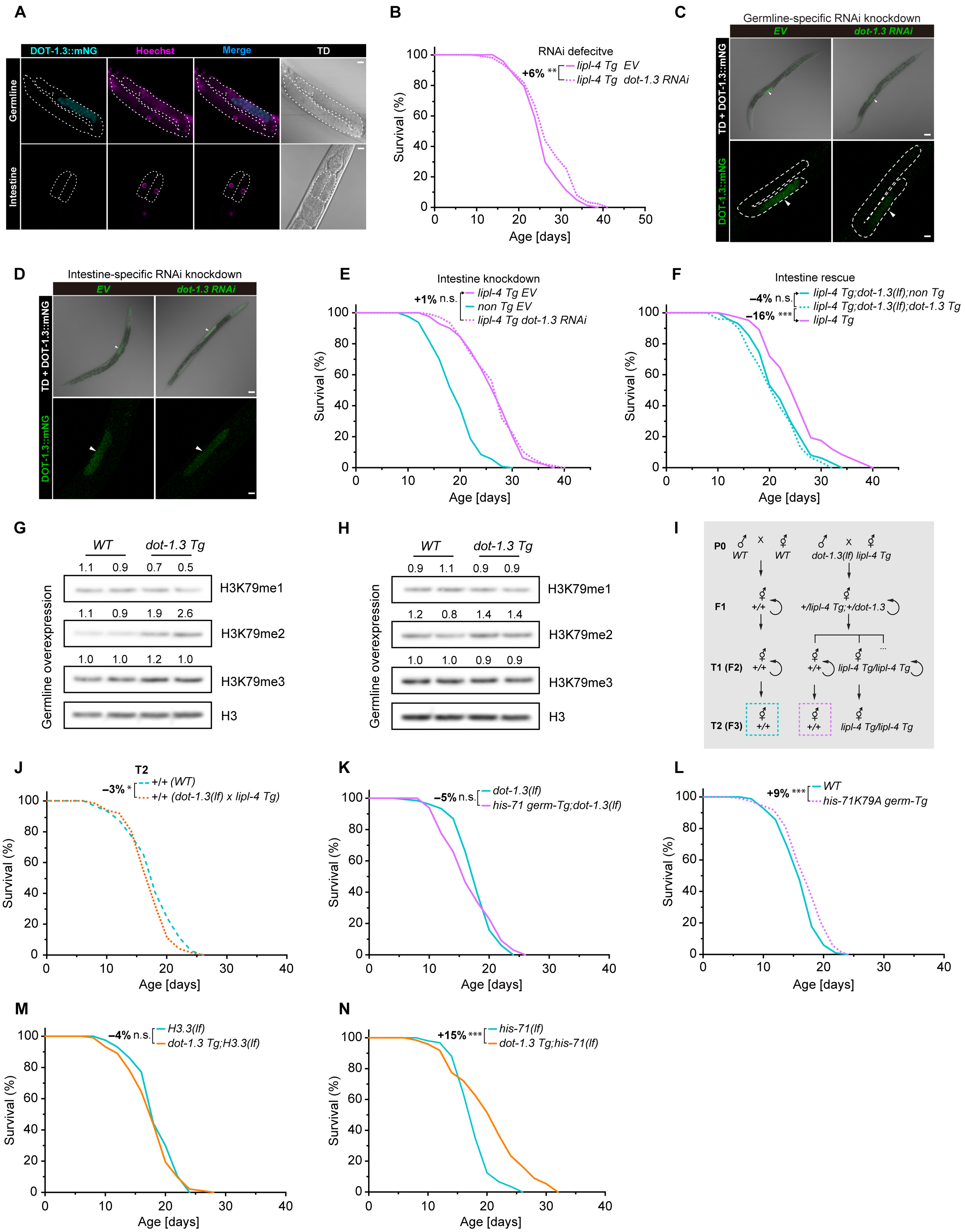
A
DOT-1.3::mNG
Hoechst
Merge
TD
Germline
Intestine
B
RNAi defecitve
+6% **
lipl-4 Tg EV
lipl-4 Tg dot-1.3 RNAi
Survival (%)
Age [days]
C
Germline-specific RNAi knockdown
EV
dot-1.3 RNAi
TD + DOT-1.3::mNG
DOT-1.3::mNG
D
Intestine-specific RNAi knockdown
EV
dot-1.3 RNAi
TD + DOT-1.3::mNG
DOT-1.3::mNG
E
Intestine knockdown
+1% n.s.
lipl-4 Tg EV
non Tg EV
lipl-4 Tg dot-1.3 RNAi
Survival (%)
Age [days]
F
Intestine rescue
−4% n.s.
−16% ***
lipl-4 Tg;dot-1.3(lf);non Tg
lipl-4 Tg;dot-1.3(lf);dot-1.3 Tg
lipl-4 Tg
Survival (%)
Age [days]
G
WT
dot-1.3 Tg
Germline overexpression
1.1 0.9 0.7 0.5
H3K79me1
1.1 0.9 1.9 2.6
H3K79me2
1.0 1.0 1.2 1.0
H3K79me3
H3
H
WT
dot-1.3 Tg
Germline overexpression
0.9 1.1 0.9 0.9
H3K79me1
1.2 0.8 1.4 1.4
H3K79me2
1.0 1.0 0.9 0.9
H3K79me3
H3
I
P0
WT
WT
dot-1.3(lf) lipl-4 Tg
F1
+/+
+/lipl-4 Tg;+/dot-1.3
T1 (F2)
+/+
+/+
lipl-4 Tg/lipl-4 Tg
T2 (F3)
+/+
+/+
lipl-4 Tg/lipl-4 Tg
J
T2
−3% *
+/+ (WT)
+/+ (dot-1.3(lf) x lipl-4 Tg)
Survival (%)
Age [days]
K
−5% n.s.
dot-1.3(lf)
his-71 germ-Tg;dot-1.3(lf)
Survival (%)
Age [days]
L
+9% ***
WT
his-71K79A germ-Tg
Survival (%)
Age [days]
M
−4% n.s.
H3.3(lf)
dot-1.3 Tg;H3.3(lf)
Survival (%)
Age [days]
N
+15% ***
his-71(lf)
dot-1.3 Tg;his-71(lf)
Survival (%)
Age [days]

fig. S5. Tissue-specific RNAi knockdown validation and interaction between DOT-1.3 and HIS-71 in regulation of longevity, and additional replicates. (**A**) Confocal images showing DOT-1.3::mNeonGreen fluorescent signals detected in the germline using a CRISPR knock-in line. Cyan, DOT-1.3::mNeonGreen; magenta, Hoechst stained nuclei. TD, Transmission Detection. Dotted lines indicate germline (top) or intestine (bottom) cells. Scale bar = 10 μm. (**B**) In *rde-1* null mutant where RNAi is defective, *dot-1.3* RNAi fails to reduce lifespan of *lipl-4 Tg* worms. (**C**, **D**) Validation of germline- or intestine-specific *dot-1.3* knockdown. Top: merged images of DOT-1.3::mNeonGreen fluorescence (arrowheads) and TD imaging (20× objective); bottom: enlarged fluorescent images. Dotted lines indicate germline (C). Scale bar = 10 μm. (**E**) Intestine-specific knockdown of *dot-1.3* does not shorten lifespan of *lipl-4 Tg* worms. (**F**) Restoration of *dot-1.3* expression in the intestine of *lipl-4 Tg;dot-1.3(lf)* mutant does not restore lifespan to the level of *lipl-4 Tg* alone. (**G**, **H**) Western blot replicates on worms with germline-specific *dot-1.3* overexpression. (**I**) Scheme representing genetic crosses and transgenerational groups (dotted boxes) used in lifespan analyses. (**J**) T2 WT descendants from the cross between *lipl-4 Tg* hermaphrodites and *dot-1.3(lf)* males do not show lifespan extension. (**K**) In *dot-1.3(lf)* mutant background, germline-specific overexpression of *his-71* fails to extend lifespan. (**L**) Germline-specific overexpression of HIS-71(K79A) mutant form results in negligible lifespan extension (< 10%). (**M**) In H3.3(null) mutant background, *dot-1.3 Tg* fails to extend lifespan. (**N**) *his-71(lf)* mutant alone does not suppress the lifespan extension in *dot-1.3 Tg* worms. In Kaplan-Meier survival curves, n = ~90 worms (B, E, F, J-N) per replicate, 3 biological replicates; log-rank test followed by Fisher's method: n.s., $p > 0.05$, *$p < 0.05$, ***$p < 0.001$. Percentage of lifespan extension (lower vs. upper, or indicated by arrows) is labeled. Summary of lifespan replicates shown in tables S1, S2 and S4.

Fig. S6.

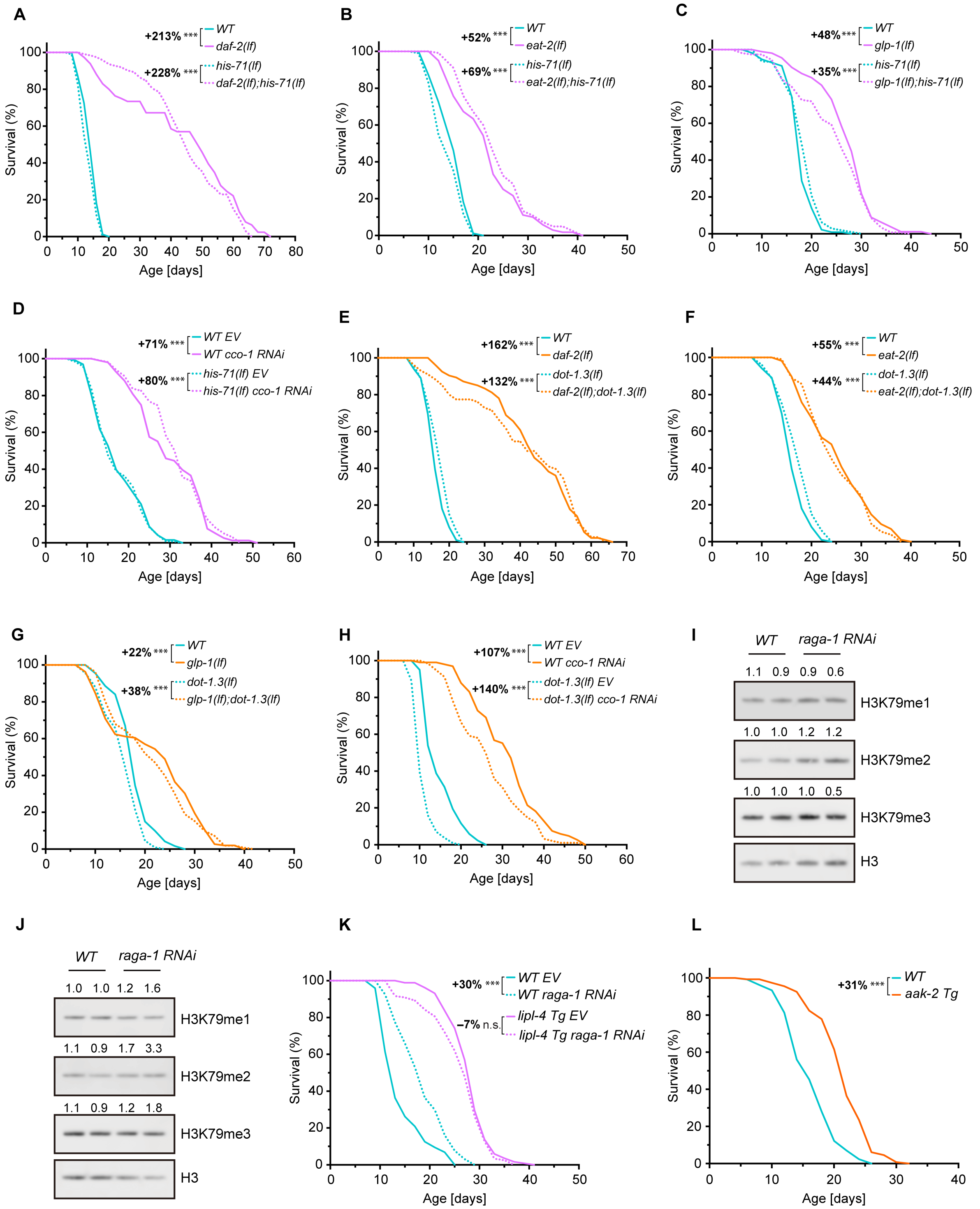
A
+213% *** WT
daf-2(lf)
+228% *** his-71(lf)
daf-2(lf);his-71(lf)
Survival (%)
Age [days]
B
+52% *** WT
eat-2(lf)
+69% *** his-71(lf)
eat-2(lf);his-71(lf)
C
+48% *** WT
glp-1(lf)
+35% *** his-71(lf)
glp-1(lf);his-71(lf)
D
+71% *** WT EV
WT cco-1 RNAi
+80% *** his-71(lf) EV
his-71(lf) cco-1 RNAi
E
+162% *** WT
daf-2(lf)
+132% *** dot-1.3(lf)
daf-2(lf);dot-1.3(lf)
F
+55% *** WT
eat-2(lf)
+44% *** dot-1.3(lf)
eat-2(lf);dot-1.3(lf)
G
+22% *** WT
glp-1(lf)
+38% *** dot-1.3(lf)
glp-1(lf);dot-1.3(lf)
H
+107% *** WT EV
WT cco-1 RNAi
+140% *** dot-1.3(lf) EV
dot-1.3(lf) cco-1 RNAi
I
WT raga-1 RNAi
1.1 0.9 0.9 0.6
H3K79me1
1.0 1.0 1.2 1.2
H3K79me2
1.0 1.0 1.0 0.5
H3K79me3
H3
J
WT raga-1 RNAi
1.0 1.0 1.2 1.6
H3K79me1
1.1 0.9 1.7 3.3
H3K79me2
1.1 0.9 1.2 1.8
H3K79me3
H3
K
+30% *** WT EV
WT raga-1 RNAi
−7% n.s. lipl-4 Tg EV
lipl-4 Tg raga-1 RNAi
L
+31% *** WT
aak-2 Tg

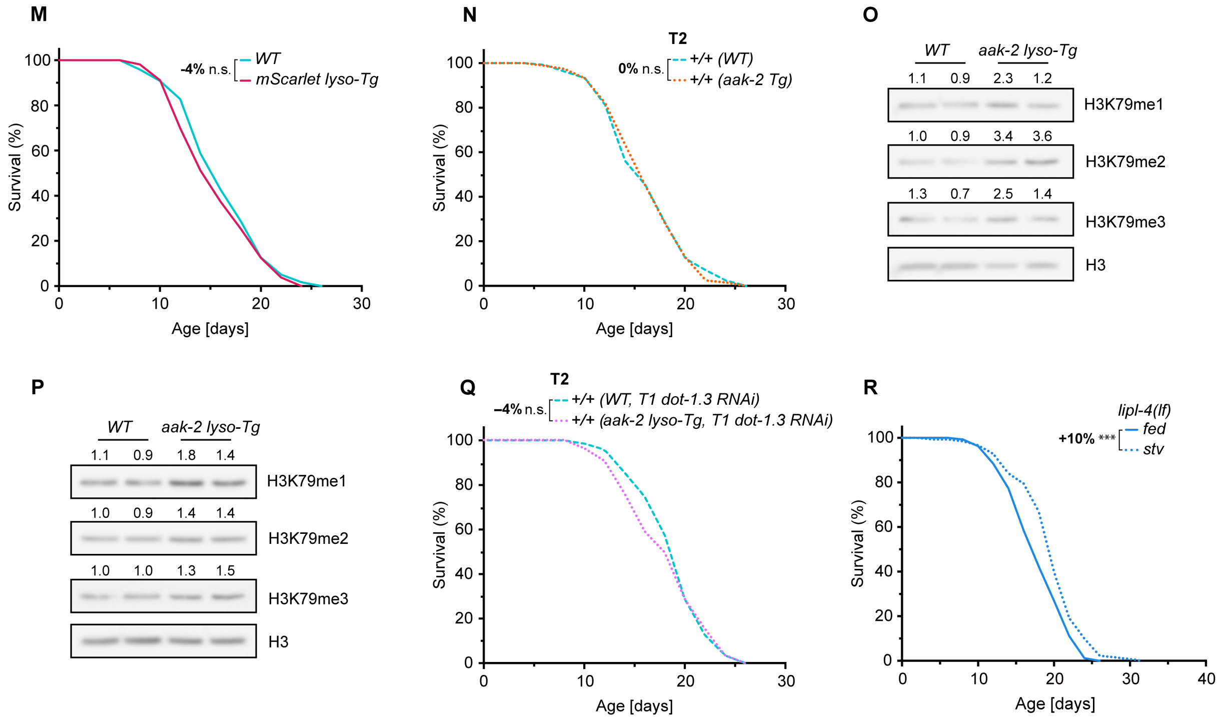
M
-4% n.s.
WT
mScarlet lyso-Tg
Survival (%)
Age [days]
N
T2
0% n.s.
+/+ (WT)
+/+ (aak-2 Tg)
Survival (%)
Age [days]
O
WT
aak-2 lyso-Tg
1.1 0.9 2.3 1.2
H3K79me1
1.0 0.9 3.4 3.6
H3K79me2
1.3 0.7 2.5 1.4
H3K79me3
H3
P
WT
aak-2 lyso-Tg
1.1 0.9 1.8 1.4
H3K79me1
1.0 0.9 1.4 1.4
H3K79me2
1.0 1.0 1.3 1.5
H3K79me3
H3
Q
T2
−4% n.s.
+/+ (WT, T1 dot-1.3 RNAi)
+/+ (aak-2 lyso-Tg, T1 dot-1.3 RNAi)
Survival (%)
Age [days]
R
lipl-4(lf)
+10% ***
fed
stv
Survival (%)
Age [days]

fig. S6. Interaction between longevity-promoting mechanisms and DOT-1.3 and HIS-71, additional replicates. (**A**-**H**) The *his-71(lf)* (A-D) or the *dot-1.3(lf)* (E-H) mutant does not diminish the pro-longevity effects associated with the *daf-2(lf)*, *glp-1(lf)*, or *eat-2(lf)* mutant, or with the *cco-1* RNAi knockdown. (**I**, **J**) Western blot replicates on worms with *raga-1* RNAi knockdown. (**K**) RNAi inactivation of *raga-1* does not further enhance lifespan extension caused by *lipl-4 Tg*. (**L**) Overexpression of *aak-2* without lysosomal tethering extends lifespan. (**M**) Transgenic worms overexpressing lysosome-tethered *mScarlet* in the intestine (*mScarlet lyso-Tg*) exhibit a lifespan comparable to WT worms. (**N**) Overexpression of *aak-2* without lysosomal tethering does not promote transgenerational longevity. (**O**, **P**) Western blot replicates on *aak-2 lyso-Tg* worms. (**Q**) Knockdown of *dot-1.3* in T1 WT descents of *aak-2 lyso-Tg* worms abolishes the transgenerational pro-longevity effect in T2 WT progeny. (**R**) Lifespan extension induced by starvation was reduced from 19% (WT) to 10% by the *lipl-4(lf)* mutant. In Kaplan-Meier survival curves, n = ~90 per replicate, 3 biological replicates; log-rank test followed by Fisher's method: n.s., $p > 0.05$, $***p < 0.001$. Percentage of lifespan extension (lower vs. upper as indicated) is labeled. Summary of lifespan replicates shown in tables S1, S2 and S4.
