# Supplementary material for: Lysosomes Signal through the Epigenome to Regulate Longevity across Generations
Source: Science. Author manuscript; Available in PMC 2026 Jan 24. (PMC12831228; doi:10.1126/science.adn8754)
Supplement: Supplemental code_20250606 [file NIHMS2127653-supplement-Supplemental_code_20250606.docx]

**Supplementary Code**

1. Overview

This document describes the ImageJ/FIJI plugin developed for this study, named Cell Radial Profiling (version 1.1). The plugin segments oocyte and quantitatively assesses immunofluorescence intensities of the intestine-exported HIS-71::3×FLAG, extending from the oocyte nucleus to the periphery in *C. elegans*, by calling the FUJI/ImageJ Radial Profile Extended plugin (https://imagej.net/ij/plugins/radial-profile-ext.html or questpharma.u-strasbg.fr/html/radial-profile-ext.html). The Cell Radial Profiling plugin was used to generate data presented in Fig.3 F-I of the main manuscript.

The plugin is included as a single file:

- Cell Radial Profiling.ijm (macros)

1. Requirements

This plugin is compatible with FIJI/ImageJ version 1.53t or later. FIJI can be downloaded from <https://imagej.net/software/fiji/>

1. Installation instructions for ‘Cell Radial Profiling.ijm’ macro plugin
2. The Radial Profile Extended plugin should be pre-installed. The plugin file (*.jar) can be downloaded from <http://questpharma.u-strasbg.fr/html/radial-profile-ext.html>, and installed via FIJI 🡪 Plugins 🡪 Install 🡪 Radial_Profile_Angle.jar.
3. Install Cell Radial Profiling (version 1.1) in FIJI/ImageJ. Navigate to FIJI 🡪 Plugins 🡪 Macros 🡪 install 🡪 Cell Radial Profiling_v1.1.ijm.
4. Usage instructions
5. Lunch FIJI and open the example image (*.oir format). In the Bio-Format Import Options window, select Split Channels.
6. Navigate to Plugins 🡪 Macros 🡪 Cell Radial Profiling_v1.1.
7. In the How to proceed window, choose Yes or No, then click OK.
8. Encircle the cell boundaries of interested. Then, right-click and select Add to ROI Manager (important). Click OK.
9. Encircle the nuclear boundaries in the encircled cell. Then, right-click and select Add to ROI Manager (important). Click OK.
10. Tick the option boxes to confirm that the above steps were completed successfully (important). Click OK.
11. Wait ~2 seconds for processing. Mark the center of nucleus using the mouse cursor. Click OK.
12. Wait ~2 seconds for processing. Save the Result.csv file, which contains radius values and intensity profiles (1 to 4), corresponding to integrations starting from angles of -135, -45, +45, and +135 degrees, respectively, as used in this study. Save the coordinates of the nucleus center as desired.
13. Code script description

| macro "Cell Radial Profiling_v1.1"  {  //Cell Radial Profiling Plugin  //Version: 1.1  //Author: Qinghao Zhang  //Licensed under the MIT License – see LICENSE.txt  //Copyright (c) 2025  //"Cell Radial Profiling_v1.1_Qinghao"  newNames = newArray("1", "2", "3"); // Set the desired new names for the images  for (i = 0; i < 3; i++) {  selectImage(i + 1); // Select the image by its index  title = getTitle(); // Get the current title of the image  newTitle = newNames[i] + ".tif"; // Add the desired new name and file extension  run("Rename...", "title=" + newTitle); // Set the new title  run("8-bit"); //set images to 8-bit  }  selectImage("2.tif");  run("Duplicate...", "title=2a.tif");  selectImage("1.tif");  run("Enhance Contrast...", "saturated=0.35");  run("Apply LUT");  selectImage("2.tif");  run("Enhance Contrast...", "saturated=0.35");  run("Apply LUT");  selectImage("3.tif");  run("Brightness/Contrast...", "minimum=35 maximum=150");  run("Apply LUT");  run("Merge Channels...", "c4=3.tif c5=2.tif c6=1.tif");  run("Rename...", "title=composite.tif");  selectWindow("composite.tif");  run("RGB Color");  setTool("polygon");  roiManager("Show None");  // Create a dialog box  Dialog.create("How to proceed?");  Dialog.addChoice("Do you need nuclear boundary assistance?", newArray("No", "Yes"));  Dialog.show();  // Get the user's choice  choice = Dialog.getChoice();  // Perform actions based on the user's choice  if (choice == "No") {  run("Clear Results");  waitForUser("First, encircle CELL boundaries. Then, right-click and select Add to ROI Manager before processing!");  setTool("ellipse");  waitForUser("Second, encircle NUCLEUS boundaries. Then, right-click and select Add to ROI Manager before processing!");      while (true) {  count= roiManager("count");  if (count == 2) {  break;}  else {  Dialog.create("Boundary selection");  waitForUser("First, encircle CELL boundaries. Then, right-click and select Add to ROI Manager before processing!");  waitForUser("Second, encircle NUCLEUS boundaries. Then, right-click and select Add to ROI Manager before processing!");  setTool("ellipse");  continue  }  }  Dialog.create("Boundary selection");  Dialog.addCheckbox("First, encircle CELL boundaries. Then, right-click and select Add to ROI Manager before processing!", false);  Dialog.addCheckbox("Second, encircle NUCLEUS boundaries. Then, right-click and select Add to ROI Manager before processing!", false);  Dialog.show();  cb = Dialog.getCheckbox();  nb = Dialog.getCheckbox();  while (true) {  if (cb) {break;}  else {  Dialog.create("Boundary selection");  Dialog.addCheckbox("First, encircle CELL boundaries. Then, right-click and select Add to ROI Manager before processing!", false);  Dialog.show();  cb = Dialog.getCheckbox();  }  }    while (true) {  if (nb) {break;}  else {  Dialog.create("Boundary selection");  Dialog.addCheckbox("Second, encircle NUCLEUS boundaries. Then, right-click and select Add to ROI Manager before processing!", false);  Dialog.show();  setTool("ellipse");  nb = Dialog.getCheckbox();  }  }  selectWindow("composite.tif");  roiManager("Add");  roiManager("Select", 0);  roiManager("Show All with labels");  selectWindow("2a.tif");  roiManager("Select", 0);  run("Measure");  setResult("Label", 0, "Custom Area");  selectWindow("2a.tif");  roiManager("Select", 0);  run("Make Inverse");  setBackgroundColor(0, 0, 0);  run("Clear", "slice");  roiManager("Select", 0);  run("Duplicate...", "2a-1.tif");  selectWindow("composite.tif");  roiManager("Select", 1);  roiManager("Show All with labels");  selectWindow("2a.tif");  roiManager("Select", 1);  run("Measure");  // Set the base label names  baseLabelCeArea = "Cell";  baseLabelNuArea = "Nucleus";  // Loop over the desired number of measurements  numMeasurements = nResults/2; // Change this value as per your requirement  for (i = 1; i <= numMeasurements; i++) {  // Construct the label names with the numeric variable  labelCeArea = baseLabelCeArea + "_" + i;  labelNuArea = baseLabelNuArea + "_" + i;    // Change the label names in the results table  h= 2*(i-1);  k= h+1;  setResult("Label", h, labelCeArea);  setResult("Label", k, labelNuArea);  }  selectWindow("2a-1.tif");  run("Copy");  newImage("Ready-to-Radius.tif", "8-bit black", 600, 600, 1);  run("Paste");  run("Enhance Contrast...", "saturated=0.35");  selectWindow("composite.tif");  roiManager("Select", 1);  run("Add Selection...");  run("Flatten");  selectWindow("composite.tif");  close();  selectWindow("2a.tif");  close();  selectWindow("2a-1.tif");  close();      ////Call radial angle profile  while (true) {  selectWindow("Ready-to-Radius.tif");  setTool("point");  waitForUser("Select nucleus center on the image");  s = selectionType();  if (s == -1) {  //exit("There was no selection.");  waitForUser("There was no selection.");  continue;    } else if (s != 10) {  //exit("The selection wasn't a point selection.");  waitForUser("The selection wasn't a point selection.");  continue;  } else {  getSelectionCoordinates(xPoints, yPoints);  Coord_x = xPoints[0];  Coord_y = yPoints[0];  showMessage("Nucleus center location (" + Coord_x + "," + Coord_y + ")");  break;  }  while  }  roiManager("reset");  makePoint(Coord_x, Coord_y, "small yellow hybrid");  roiManager("Add");  List.setMeasurements;  print("Coord_x=" + List.getValue("X") + " Coord_y=" + List.getValue("Y"));      selectWindow("Ready-to-Radius.tif");  roiManager("Select", 0);  run("Radial Profile Angle", "x_center=" + Coord_x + " y_center=" + Coord_y + " radius=" + 424.3 + " starting_angle=" + -135 + " integration_angle=" + 45 + " calculate_radial_profile_on_slack");  for(i = 0; i != Ext.getBinSize; i++)  {  //setResult("R_1", i, 1);  setResult("Radius", i, Ext.getXValue(i));  setResult("Intensity_1",i, Ext.getYValue(0,i));  }  updateResults();      selectWindow("Ready-to-Radius.tif");  roiManager("Select", 0);  run("Radial Profile Angle", "x_center=" + Coord_x + " y_center=" + Coord_y + " radius=" + 424.3 + " starting_angle=" + -45 + " integration_angle=" + 45 + " calculate_radial_profile_on_slack");  for(a = 0; a != Ext.getBinSize; a++)  {  //setResult("R_2", a, 2);  //setResult("Radius_2", a, Ext.getXValue(a));  setResult("Intensity_2",a, Ext.getYValue(0,a));  }  updateResults();  selectWindow("Ready-to-Radius.tif");  roiManager("Select", 0);  run("Radial Profile Angle", "x_center=" + Coord_x + " y_center=" + Coord_y + " radius=" + 424.3 + " starting_angle=" + 45 + " integration_angle=" + 45 + " calculate_radial_profile_on_slack");  for(b = 0; b != Ext.getBinSize; b++)  {  //setResult("R_3", b, 3);  //setResult("Radius_3", b, Ext.getXValue(b));  setResult("Intensity_3",b, Ext.getYValue(0,b));  }  updateResults();  selectWindow("Ready-to-Radius.tif");  roiManager("Select", 0);  run("Radial Profile Angle", "x_center=" + Coord_x + " y_center=" + Coord_y + " radius=" + 424.3 + " starting_angle=" + 135 + " integration_angle=" + 45 + " calculate_radial_profile_on_slack");  for(c = 0; c != Ext.getBinSize; c++)  {  //setResult("R_4", c, 4);  //setResult("Radius_4", c, Ext.getXValue(c));  setResult("Intensity_4",c, Ext.getYValue(0,c));  }  updateResults();  selectWindow("Ready-to-Radius");  close();  selectWindow("Ready-to-Radius");  close();  selectWindow("Ready-to-Radius");  close();  selectWindow("Ready-to-Radius");  close();  roiManager("reset");  run("Close All");  saveAs("Results");  run("Clear Results");  updateResults();    //if choose No  } else if (choice == "Yes") {    run("Clear Results");  waitForUser("First, encircle CELL boundaries. Then, right-click and select Add to ROI Manager before processing!");  setTool("ellipse");  waitForUser("Second, encircle NUCLEUS boundaries. Then, right-click and select Add to ROI Manager before processing!");      while (true) {  count= roiManager("count");  if (count == 2) {  break;}  else {  Dialog.create("Boundary selection");  waitForUser("First, encircle CELL boundaries. Then, right-click and select Add to ROI Manager before processing!");  waitForUser("Second, encircle NUCLEUS boundaries. Then, right-click and select Add to ROI Manager before processing!");  setTool("ellipse");  continue  }  }  Dialog.create("Boundary selection");  Dialog.addCheckbox("First, encircle CELL boundaries. Then, right-click and select Add to ROI Manager before processing!", false);  Dialog.addCheckbox("Second, encircle NUCLEUS boundaries. Then, right-click and select Add to ROI Manager before processing!", false);  Dialog.show();  cb = Dialog.getCheckbox();  nb = Dialog.getCheckbox();  while (true) {  if (cb) {break;}  else {  Dialog.create("Boundary selection");  Dialog.addCheckbox("First, encircle CELL boundaries. Then, right-click and select Add to ROI Manager before processing!", false);  Dialog.show();  cb = Dialog.getCheckbox();  }  }    while (true) {  if (nb) {break;}  else {  Dialog.create("Boundary selection");  Dialog.addCheckbox("Second, encircle NUCLEUS boundaries. Then, right-click and select Add to ROI Manager before processing!", false);  Dialog.show();  setTool("ellipse");  nb = Dialog.getCheckbox();  }  }  selectWindow("composite.tif");  roiManager("Add");  roiManager("Select", 0);  roiManager("Show All with labels");  selectWindow("2a.tif");  roiManager("Select", 0);  run("Measure");  setResult("Label", 0, "Custom Area");  selectWindow("2a.tif");  roiManager("Select", 0);  run("Make Inverse");  setBackgroundColor(0, 0, 0);  run("Clear", "slice");  roiManager("Select", 0);  run("Duplicate...", "2a-1.tif");    selectWindow("composite.tif");  roiManager("Select", 1);  run("Add Selection...");  run("Flatten");  selectWindow("composite-1.tif");  roiManager("Select", 0);  run("Make Inverse");  setBackgroundColor(0, 0, 0);  run("Clear", "slice");  roiManager("Select", 0);  run("Duplicate...", "composite-1-1.tif");    selectWindow("composite.tif");  roiManager("Select", 1);  roiManager("Show All with labels");  selectWindow("2a.tif");  roiManager("Select", 1);  run("Measure");  // Set the base label names  baseLabelCeArea = "Cell";  baseLabelNuArea = "Nucleus";  // Loop over the desired number of measurements  numMeasurements = nResults/2; // Change this value as per your requirement  for (i = 1; i <= numMeasurements; i++) {  // Construct the label names with the numeric variable  labelCeArea = baseLabelCeArea + "_" + i;  labelNuArea = baseLabelNuArea + "_" + i;    // Change the label names in the results table  h= 2*(i-1);  k= h+1;  setResult("Label", h, labelCeArea);  setResult("Label", k, labelNuArea);  }    selectWindow("2a-1.tif");  run("Copy");  newImage("Ready-to-Radius.tif", "8-bit black", 600, 600, 1);  run("Paste");      selectWindow("composite-1-1.tif");  run("Copy");  newImage("Ready-to-Radius_composite.tif", "RGB black", 600, 600, 1);  run("Paste");  //run("Enhance Contrast...", "saturated=0.35");//    selectWindow("composite.tif");  close();  selectWindow("composite-1.tif");  close();  selectWindow("composite-1-1.tif");  close();  selectWindow("2a.tif");  close();  selectWindow("2a-1.tif");  close();      ////Radial angle profile  while (true) {  selectWindow("Ready-to-Radius_composite.tif");  setTool("point");  waitForUser("Select nucleus center on the image");  s = selectionType();  if (s == -1) {  //exit("There was no selection.");  waitForUser("There was no selection.");  continue;    } else if (s != 10) {  //exit("The selection wasn't a point selection.");  waitForUser("The selection wasn't a point selection.");  continue;  } else {  getSelectionCoordinates(xPoints, yPoints);  Coord_x = xPoints[0];  Coord_y = yPoints[0];  showMessage("Nucleus center location (" + Coord_x + "," + Coord_y + ")");  break;  }  while  }  roiManager("reset");  makePoint(Coord_x, Coord_y, "small yellow hybrid");  roiManager("Add");  List.setMeasurements;  print("Coord_x=" + List.getValue("X") + " Coord_y=" + List.getValue("Y"));    selectWindow("Ready-to-Radius_composite.tif");  close();      selectWindow("Ready-to-Radius.tif");  roiManager("Select", 0);  roiManager("Update");  run("Radial Profile Angle", "x_center=" + Coord_x + " y_center=" + Coord_y + " radius=" + 424.3 + " starting_angle=" + -135 + " integration_angle=" + 45 + " calculate_radial_profile_on_slack");  for(i = 0; i != Ext.getBinSize; i++)  {  //setResult("R_1", i, 1);  setResult("Radius", i, Ext.getXValue(i));  setResult("Intensity_1",i, Ext.getYValue(0,i));  }  updateResults();      selectWindow("Ready-to-Radius.tif");  roiManager("Select", 0);  roiManager("Update");  run("Radial Profile Angle", "x_center=" + Coord_x + " y_center=" + Coord_y + " radius=" + 424.3 + " starting_angle=" + -45 + " integration_angle=" + 45 + " calculate_radial_profile_on_slack");  for(a = 0; a != Ext.getBinSize; a++)  {  //setResult("R_2", a, 2);  //setResult("Radius_2", a, Ext.getXValue(a));  setResult("Intensity_2",a, Ext.getYValue(0,a));  }  updateResults();    selectWindow("Ready-to-Radius.tif");  roiManager("Select", 0);  roiManager("Update");  run("Radial Profile Angle", "x_center=" + Coord_x + " y_center=" + Coord_y + " radius=" + 424.3 + " starting_angle=" + 45 + " integration_angle=" + 45 + " calculate_radial_profile_on_slack");  for(b = 0; b != Ext.getBinSize; b++)  {  //setResult("R_3", b, 3);  //setResult("Radius_3", b, Ext.getXValue(b));  setResult("Intensity_3",b, Ext.getYValue(0,b));  }  updateResults();    selectWindow("Ready-to-Radius.tif");  roiManager("Select", 0);  roiManager("Update");  run("Radial Profile Angle", "x_center=" + Coord_x + " y_center=" + Coord_y + " radius=" + 424.3 + " starting_angle=" + 135 + " integration_angle=" + 45 + " calculate_radial_profile_on_slack");  for(c = 0; c != Ext.getBinSize; c++)  {  //setResult("R_4", c, 4);  //setResult("Radius_4", c, Ext.getXValue(c));  setResult("Intensity_4",c, Ext.getYValue(0,c));  }  updateResults();    selectWindow("Ready-to-Radius");  close();  selectWindow("Ready-to-Radius");  close();  selectWindow("Ready-to-Radius");  close();  selectWindow("Ready-to-Radius");  close();  roiManager("reset");  run("Close All");  saveAs("Results");  run("Clear Results");  updateResults();  }  } |
| --- |
