# Supplementary material for: Lysosomes Signal through the Epigenome to Regulate Longevity across Generations
Source: Science. Author manuscript; Available in PMC 2026 Jan 24. (PMC12831228; doi:10.1126/science.adn8754)
Supplement: Table S3_qPCR Ct values_20250204 [file NIHMS2127653-supplement-Table_S3_qPCR_Ct_values_20250204.pdf]

Table S3. Validation of intestine-specific expression of *his-71::3xflag* by qPCR.

Genotype	Tissue	Rep	Gene targets			Internal ctrl gene			Lab Code Strain Number
			name	CT value	mean ± s.e.	name	CT value	mean ± s.e.	
his-71::3xflag int-Tg	intestine	#1	*his-71::3xflag*	22.230	22.2 ± 0.119	*rpl-32*	22.052	21.8 ± 0.211	MCW1628
		#2		22.313			21.355		
		#3		21.921			21.900		
	germline	#1	*his-71::3xflag*	32.509	32.1 ± 0.234	*rpl-32*	17.512	17.8 ± 0.136	
		#2		31.934			17.802		
		#3		31.726			17.980		
