# Supplementary material for: Lysosomes Signal through the Epigenome to Regulate Longevity across Generations
Source: Science. Author manuscript; Available in PMC 2026 Jan 24. (PMC12831228; doi:10.1126/science.adn8754)
Supplement: adn8754_ReproducibilityChecklist_20250608 [file NIHMS2127653-supplement-adn8754_ReproducibilityChecklist_20250608.docx]

**Materials Design Analysis Reporting (MDAR)**

**Checklist for Authors**

The MDAR framework establishes a minimum set of requirements in transparent reporting applicable to studies in the life sciences (see Statement of Task: doi:10.31222/osf.io/9sm4x.). The MDAR checklist is a tool for authors, editors, and others seeking to adopt the MDAR framework for transparent reporting in manuscripts and other outputs. Please refer to the MDAR Elaboration Document for additional context for the MDAR framework.

**For all that apply, please note where in the manuscript the required information is provided.**

**Materials:**

| **Newly created materials** | **indicate where provided: page no/section/legend)** | **n/a** |
| --- | --- | --- |
| The manuscript includes a dedicated "materials availability statement" providing transparent disclosure about availability of newly created materials including details on how materials can be accessed and describing any restrictions on access. | The worm strains newly generated and utilized in this study are comprehensively detailed in Supplementary Table S6, titled “List of *C. elegans* Strains Used in This Study.” Each strain is assigned a unique laboratory code for clear identification. Researchers seeking to access these strains should contact the corresponding author. |  |
|  |  |  |
| **Antibodies** | **indicate where provided: page no/section/legend)** | **n/a** |
| For commercial reagents, provide supplier name, catalogue number and [RRID](https://scicrunch.org/resources), if available. | Antibody details, including supplier name and catalog numbers, are provided in the Materials and Methods section. Corresponding RRID information is listed below.   \| **Antibody Names** \| **Supplier #Catlog** \| **RRID** \| \| --- \| --- \| --- \| \| anti-H3K79me1 \| CST #12522 \| AB_2797942 \| \| anti-H3K79me2 \| CST #5427 \| AB_10693787 \| \| anti-H3K79me3 \| CST #4260 \| AB_10828006 \| \| anti-H3 \| CST #4499 \| AB_10544537 \| \| anti-β-actin \| Santa Cruz #sc-47778 \| AB_626632 \| \| anti-α-tubulin \| Abcam #ab52866 \| AB_869989 \| \| anti-rabbit IgG-HRP \| Santa Cruz #sc-2357 \| AB_628497 \| \| anti-mouse m-IgG Fc 90 BP-HRP \| Santa Cruz #sc-525409 \| AB_3101828 \| \| (ChIP-seq) anti-histone H3 \| CST #4620 \| AB_1904005 \| \| (ChIP-seq) anti-H3K79me2 \| CST #5427 \| AB_10693787 \| \| (ChIP-seq) anti-H3K79me3 \| Abcam #ab2621 \| AB_303215 \| \| anti-FLAG® M2 \| Sigma #F3165 \| AB_259529 \| |  |
|  |  |  |
| **DNA and RNA sequences** | **indicate where provided: page no/section/legend)** | **n/a** |
| **Short novel DNA or RNA including primers, probes:** Sequences should be included or deposited in a public repository. | The oligonucleotide sequences utilized in this study are comprehensively detailed in Supplementary Table S5, titled “List of Oligonucleotide Sequences.” |  |
|  |  |  |
| **Cell materials** | **indicate where provided: page no/section/legend** | **n/a** |
| **Cell lines:** Provide species information, strain. Provide accession number in repository **OR** supplier name, catalog number, clone number, **OR** RRID. |  | n/a |
| **Primary cultures:** Provide species, strain, sex of origin, genetic modification status. |  | n/a |
|  |  |  |
| **Experimental animals** | **indicate where provided: page no/section/legend)** | **n/a** |
| **Laboratory animals or Model organisms:** Provide species, strain, sex, age, genetic modification status. Provide accession number in repository **OR** supplier name, catalog number, clone number, **OR** RRID. | In this study, the model organism *Caenorhabditis elegans* was employed. The worm strains used are comprehensively detailed in Supplementary Table S6, titled "List of *C. elegans* Strains Used in This Study", with each strain assigned a unique laboratory code for identification. |  |
| **Animal observed in or captured from the field:** Provide species, sex, and age where possible. |  | n/a |
|  |  |  |
| **Plants and microbes** | **indicate where provided: page no/section/legend)** | **n/a** |
| **Plants:** provide species and strain, ecotype and cultivar where relevant, unique accession number if available, and source (including location for collected wild specimens). |  | n/a |
| **Microbes:** provide species and strain, unique accession number if available, and source. | In this study, we utilized the common laboratory bacterial strains *E. coli* OP50 (streptomycin-resistant) and *E. coli* HT115. Both strains are available from the Caenorhabditis Genetics Center (CGC). |  |
|  |  |  |
| **Human research participants** | **indicate where provided: page no/section/legend) or state if these demographics were not collected** | **n/a** |
| If collected and within the bounds of privacy constraints report on age, sex and gender or ethnicity for all study participants. |  | n/a |

**Design:**

| **Study protocol** | **indicate where provided: page no/section/legend)** | **n/a** |
| --- | --- | --- |
| If study protocol has been pre-registered, provide DOI. For clinical trials, provide the trial registration number **OR** cite DOI. |  | n/a |
|  |  |  |
| **Laboratory protocol** | **indicate where provided: page no/section/legend)** | **n/a** |
| Provide DOI **OR** other citation details if detailed step-by-step protocols are available. | All essential experimental procedures are comprehensively described in the Materials and Methods section of the manuscript, with protocols from previous studies also properly cited. |  |
|  |  |  |
| **Experimental study design (statistics details)** | | |
| **For in vivo studies:** State whether and how the following have been done | **indicate where provided: page no/section/legend. If it could have been done, but was not, write not done** | **n/a** |
| Sample size determination | In this study, worm sample size information is provided in Supplementary Tables S1, S2, and S4, as well as in the figure legends and the Materials and Methods section. |  |
| Randomisation |  | n/a |
| Blinding |  | n/a |
| Inclusion/exclusion criteria | Inclusion Criteria: Age-synchronized worm samples were used in this study; further details are provided in the figure legends, the Materials and Methods section, and the Supplementary Tables S1, S2, and S4.  Exclusion Criteria: no animals were excluded from analysis. |  |
|  |  |  |
| **Sample definition and in-laboratory replication** | **indicate where provided: page no/section/legend** | **n/a** |
| State number of times the experiment was replicated in laboratory. | Detailed information on experiment replication times is available in Supplementary Tables S1, S2, and S4, as well as in the figure legends and the Materials and Methods section. |  |
| Define whether data describe technical or biological replicates. | Detailed information about technical and biological replicates is provided in the figure legends, the Materials and Methods section, and the Supplementary Tables S1, S2, and S4. |  |
|  |  |  |
| **Ethics** | **indicate where provided: page no/section/legend** | **n/a** |
| **Studies involving human participants:** State details of authority granting ethics approval (IRB or equivalent committee(s), provide reference number for approval. |  | n/a |
| **Studies involving experimental animals:** State details of authority granting ethics approval (IRB or equivalent committee(s), provide reference number for approval. |  | n/a |
| **Studies involving specimen and field samples:** State if relevant permits obtained, provide details of authority approving study; if none were required, explain why. |  | n/a |
|  |  |  |
| **Dual Use Research of Concern (DURC)** | **indicate where provided: page no/section/legend** | **n/a** |
| If study is subject to dual use research of concern regulations, state the authority granting approval and reference number for the regulatory approval. |  | n/a |

**Analysis:**

| **Attrition** | **indicate where provided: page no/section/legend** | **n/a** |
| --- | --- | --- |
| Describe whether exclusion criteria were preestablished. Report if sample or data points were omitted from analysis. If yes report if this was due to attrition or intentional exclusion and provide justification. |  | n/a |
|  |  |  |
| **Statistics** | **indicate where provided: page no/section/legend** | **n/a** |
| Describe statistical tests used and justify choice of tests. | Detailed statistical information for the experiments is provided in the Materials and Methods section, the figure legends, and Supplementary Tables S1, S2, and S4. |  |
|  |  |  |
| **Data availability** | **indicate where provided: page no/section/legend** | **n/a** |
| For newly created and reused datasets, the manuscript includes a data availability statement that provides details for access or notes restrictions on access. | A statement regarding data availability is provided in the Acknowledgements section of the manuscript. |  |
| If newly created datasets are publicly available, provide accession number in repository **OR** DOI **OR** URL and licensing details where available. | The ChIP-seq data used in this study has been submitted to GEO database and the GEO accession numbers are GSE235724, GSE290404, GSE290488. |  |
| If reused data is publicly available provide accession number in repository **OR** DOI **OR** URL, **OR** citation. | The RNA-seq data used in this study can be found in the NCBI Sequence Read Archive (SRA) and the accession codes for each biological sample are SAMN25414087, SAMN25414088, SAMN25414089, SAMN25414090, SAMN25414091, SAMN25414092. |  |
|  |  |  |
| **Code availability** | **indicate where provided: page no/section/legend** | **n/a** |
| For all newly generated custom computer code/software/mathematical algorithm or re-used code essential for replicating the main findings of the study, the manuscript includes a data availability statement that provides details for access or notes restrictions. | All software and algorithms used in this study are detailed in the Materials and Methods section, with proper citations provided.  Newly generated custom computer code can be found in the supplementary file. |  |
| If newly generated code is publicly available, provide accession number in repository, **OR** DOI **OR** URL and licensing details where available. State any restrictions on code availability or accessibility. |  | n/a |
| If reused code is publicly available provide accession number in repository **OR** DOI **OR** URL, **OR** citation. | All software and algorithms used in this study are detailed in the Materials and Methods section, with proper citations provided. |  |

**Reporting**

MDAR framework recommends adoption of discipline-specific guidelines, established and endorsed through community initiatives. Journals have their own policy about requiring specific guidelines and recommendations to complement MDAR.

| **Adherence to community standards** | **indicate where provided: page no/section/legend** | **n/a** |
| --- | --- | --- |
| State if relevant guidelines (e.g., ICMJE, MIBBI, ARRIVE) have been followed, and whether a checklist (e.g., CONSORT, PRISMA, ARRIVE) is provided with the manuscript. |  | n/a |
